# The Dinuclear Zirconocene Complex [(Cp_2_Zr)_2_(*μ*‐Me)(*μ*‐C_2_Ph)] as a Platform for Small Molecule Activation

**DOI:** 10.1002/chem.202500857

**Published:** 2025-04-07

**Authors:** Hanan Al Hamwi, Mirko Rippke, Kevin Lindenau, Anke Spannenberg, Martin Lamac, Fabian Reiß, Torsten Beweries

**Affiliations:** ^1^ Leibniz Institute for Catalysis Albert‐Einstein‐Strasse 29a Rostock Germany; ^2^ J. Heyrovsky Institute of Physical Chemistry of the Czech Academy of Sciences Dolejškova 2155/3 Prague Czech Republic

**Keywords:** DFT, dinuclear complexes, hydrides, metallocenes, zirconium

## Abstract

The dinuclear title compound [(Cp_2_Zr)_2_(*μ*‐Me)(*μ*‐C_2_Ph)] **5** was prepared from a zirconocene alkynyl methyl complex and Rosenthal's zirconocene source [Cp_2_Zr(py)(*η* ^2^‐Me_3_SiC_2_SiMe_3_)] in a formal comproportionation reaction. This complex shows catalytic activity for the dehydrocoupling of amine boranes, with a dinuclear hydride‐bridged alkynyl complex **6** being formed as a catalytically relevant species. The structure of this complex was confirmed for the first time by single‐crystal X‐ray analysis. The reaction of complex **5** with hydrogen results in hydrogenation of the alkynyl ligand, yielding a highly labile trinuclear hydride‐bridged complex as a possible intermediate of zirconocene dihydride/ethylbenzene formation. This complex shows an unusual distorted planar tetracoordinate environment at the central carbon atom positioned between the three Zr centers. The reaction of complex **5** with 2‐cyanopyridine and acetonitrile is characterized by a reduction of the substrates. The herein reported reactivity of complex **5** demonstrates the remarkable potential of well‐established dinuclear zirconocenes to stabilize unusual bond situations, which were analyzed comprehensively using spectroscopic, structural, and computational methods.

## Graphical abstract

The reported dinuclear zirconocene alkynyl methyl complex represents a multifaceted platform for the activation of small molecules. While reduction reactions furnish unusual di‐ and trinuclear hydride complexes, reactions with nitriles result in coordination and reduction of the substrate. Detailed structural and computational analysis gives insights into the structure and bonding of these species.

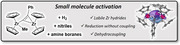

## 1 Introduction

The concept of multinuclear complexes is a well‐established but still intriguing field in organometallic chemistry and homogeneous catalysis. The idea of multinuclear complexes has gained renewed interest due to their potential to enhance orthogonal catalytic activity and selectivity in various reactions.^[^ ^1^ ^]^ For example, the development of polynuclear catalysts has greatly facilitated the production of high‐performance polyolefin materials.^[^ ^2^ ^]^ The cooperative effects of polynuclear catalysts composed of early transition metals compared to mononuclear catalysts affect catalytic performance and polymer structure through multiple factors, such as steric hindrance, electronic effects, heteroatom effects, hydrogen bonding, and the distance between metal centers in polynuclear metal catalysts.^[^ ^3^ ^]^ The fine‐tuning of these parameters allows for a wide range of variations in the future design of di‐ and polynuclear transition metal complexes, which will have customized properties that can be applied in catalysis and/or stoichiometric bond activation. The development of group 4 non‐metallocene multinuclear complexes is, in this context, an increasingly important area of current research.^[^ ^4^ ^]^ However, the metallocene‐based group 4 complexes still hold many interesting transformations of both catalytic and stoichiometric nature.

**Figure 1. a):**
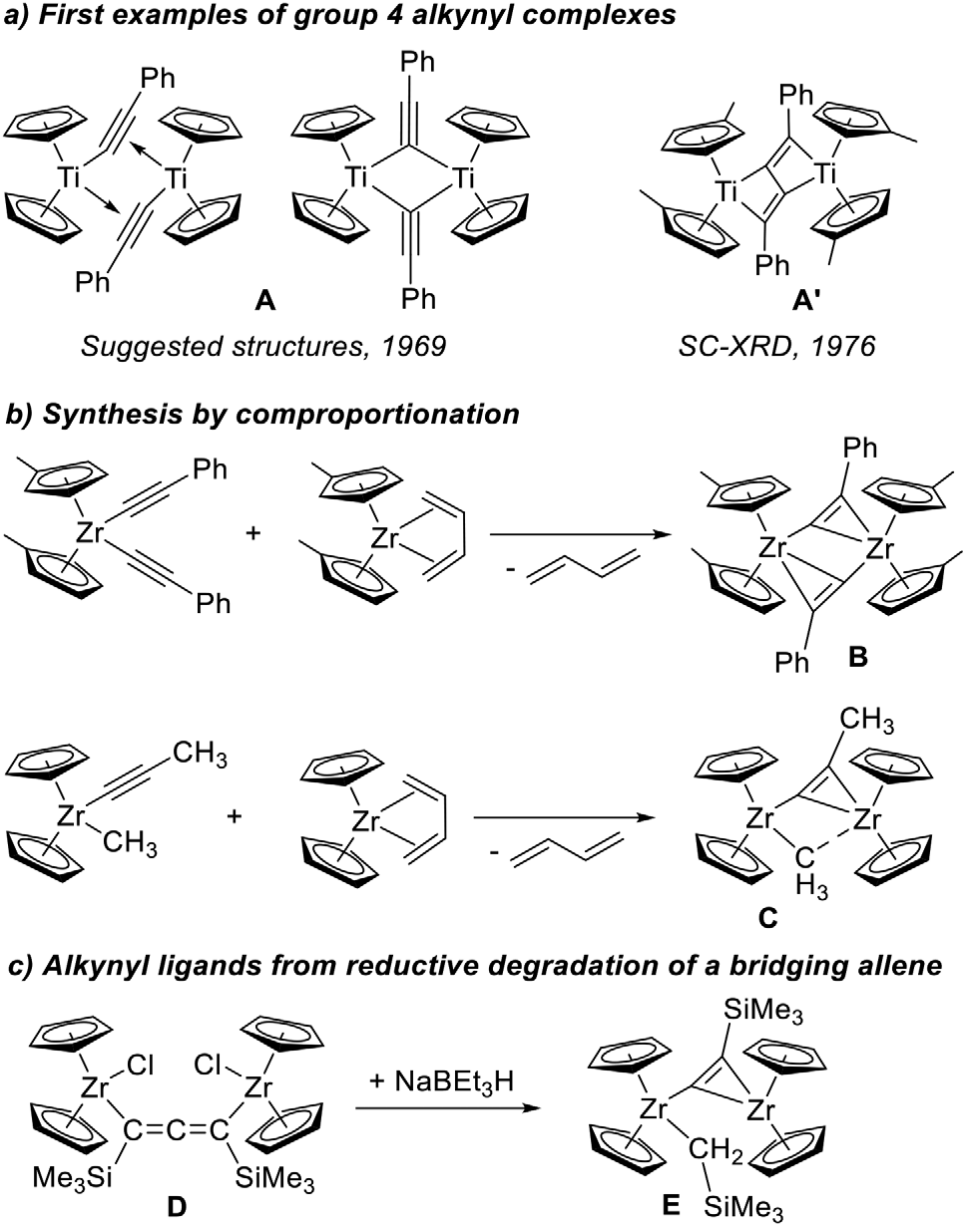
The alkynyl bridged titanocene complex **A** as two different suggested forms and the determined connectivity of a related complex **A’**; b) First example of a fully characterized alkynyl bridged zirconocene **B** and schematic representation of the comproportination approach to homo‐ and heterobimetallic alkynyl methyl bridged complexes, **C**; c) Reductive transformation of an allenediide ligand in **D**, yielding Zr alkynyl complex **E**.

The chemistry of alkynyl bridged group 4 metallocenes dates back to seminal work of Teuben and de Liefde Meijer from 1969 in which the alkynyl bridged titanocene complex (**A**, Figure 1a) was synthesized starting from the Ti(III) precursor [Cp_2_Ti(*μ*‐Cl)]_2_ (Cp = *η* ^5^‐cyclopentadienyl) and two equivalents of NaC_2_Ph.^[^ ^5^ ^]^ Mass spectrometric and IR spectroscopic analysis suggested the presence of a dinuclear complex with bridging alkynyl units. Later, unexpected C–C coupling of the alkynyl groups in **A’** was confirmed by single crystal X‐ray diffraction (SC‐XRD) analysis of the corresponding singly methylated Cp analogue.^[^ ^6^ ^]^ The first zirconocene analogue **B** was synthesized and characterized by Erker and co‐workers using a comproportionation reaction between a bis‐alkynyl zirconocene(IV) and the in situ generated zirconocene(II) (Figure 1b).^[^ ^7^ ^]^ Interestingly, this complex **B** shows no C–C coupling, but *σ*‐C*α*‐Zr binding and *π*‐coordination to the second zirconocene. Theoretical investigations were carried out to better understand the electronic situations in these and related systems.^[^ ^8^ ^]^ Later, the related dinuclear zirconocene trimethylsilylalkynyl complex [Cp_2_Zr(*μ*‐C_2_SiMe_3_)]_2_, was characterized and investigated by several research groups.^[^ ^9^ ^]^ Rosenthal and co‐workers extended the scope of these alkynyl complexes to di‐ and polynuclear Ti and Zr complexes bearing C–C activated and non‐activated butadiynes and octatetraynes and demonstrated that compounds of this type are of relevance for the single bond metathesis of 1,3‐diynes.^[^ ^10^ ^]^

Erker *et* al. further explored the concept of comproportionation reactions between bis‐alkynyl Zr(IV)/Hf(IV) and masked Zr(II)/Hf(II) butadiene metallocene complexes to prepare differently substituted complexes of the type [Cp_2_M(*μ*‐C_2_R)]_2_; M = Zr, Hf; R = H, CH_3_, Ph, C_2_H_5_, CH_2_Ph) (similar to **B**).^[^ ^11^ ^]^ This transformation can also be performed starting from metallocene alkynyl halide and alkynyl methyl complexes, yielding halide or methyl bridged dinuclear complexes (example **C**, Figure 1b), in which the alkynyl unit can oscillate between both metal centers. Recently, some of us have reported the formation of a related dinuclear alkynyl bridged zirconocene complex during catalytic dehydrocoupling of dimethylamine borane, H_3_B·NMe_2_H, using a dinuclear allenediide Zr chloride complex **D**, activated with MeLi,^[^ ^12^ ^]^ formally by cleavage of one of the C═C bonds of the C═C═C unit under reductive conditions. Targeted synthesis of this complex [(Cp_2_Zr)_2_(*μ*‐CH_2_SiMe_3_)(*μ*‐C_2_SiMe_3_)] (**E**) is possible from the Zr chloride precatalyst with NaBEt_3_H as a hydride source (Figure 1c).^[^ ^13^ ^]^ Complex **E** and related [(Cp_2_Zr)_2_(*μ*‐CH_3_)(*μ*‐C_2_SiMe_3_)] (**F**) can act as a precatalyst for the dehydrocoupling of amine boranes.

Inspired by the modular comproportionation approach for the synthesis of these dinuclear complexes that allows facile variations at both metal centers as well as at the alkynyl unit, we herein present the synthesis and characterization of the dinuclear phenylalkynyl bridged zirconocene complex [(Cp_2_Zr)_2_(*μ*‐CH_3_)(*μ*‐C_2_Ph)] along with an investigation of its reactivity toward small molecules. These studies reveal a variety of unexpected and hitherto unknown bond activation motifs. The chemically robust metallocene platform thus once again provides insights into the potential diversity of possible transformations with electropositive early transition metals and small molecules.

## 2 Results and Discussion

### 2.1 Synthesis of [(Cp_2_Zr)_2_(*μ*‐Me)(*μ*‐C_2_Ph)] (**5**)

**Scheme 1:**
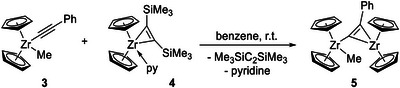
Synthesis of **5** using complex **3** and Rosenthal's reagent (**4**).

In previous studies, for example, in the synthesis of the allene bridged complex (**D**),^[^ ^14^ ^]^ trimethylsilyl‐substituted alkynyl bridged complex **F**,^[^ ^13^ ^]^ and highly strained metallacyclobutadiene complexes,^[^ ^15^ ^]^ it turned out to be crucial to isolate the organolithium precursor in pure form instead of using the commonly applied in situ lithiation protocol. Therefore, LiC_2_Ph (**1**) was prepared by slow addition of one equivalent of *n*‐BuLi to a cooled (−78°C) diethyl ether solution of freshly distilled phenylacetylene, followed by stirring overnight at ambient conditions and washing with *n*‐hexane. Reaction of **1** with [Cp_2_Zr(Me)Cl] (**2**) in toluene at −78°C produces the mononuclear complex **3** as a red oil in high yields of up to 93%. Of note, the resulting oily material contains minor amounts of unidentified Cp‐containing species (Figure S1), however, the described procedure is the best method out of the different approaches that were tested. Reaction of **3** with an equimolar amount of Rosenthal's zirconocene(II) source [Cp_2_Zr(py)(*η* ^2^‐Me_3_SiC_2_SiMe_3_)] (**4**, py = pyridine) in benzene solution at ambient temperature produces the dinuclear Zr alkynyl methyl complex **5**, which can be isolated as an orange solid material in yields of up to 77% (Scheme 1).

**Figure 2:**
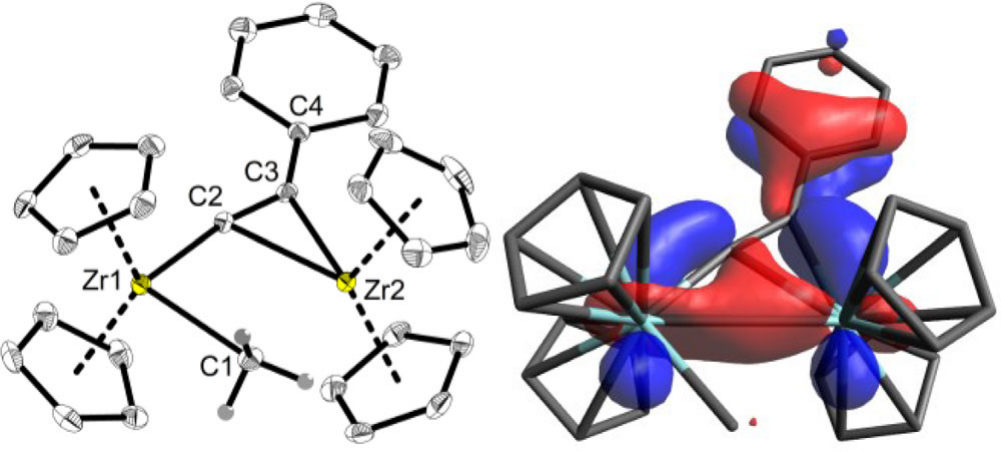
Left: Molecular structure of complex **5**. Thermal ellipsoids correspond to 30% probability at 170 K. Hydrogen atoms except at C1 are omitted for clarity. Selected bond lengths are given in Table 1. Right: NLMO38 of **5**. Analysis of the formal lone pair at C4 reveals a strong delocalization with only 14.6% of the original NBO lone pair. It shows bonding contributions between both Zr centers and anti‐bonding features of the alkynyl unit. It can be interpreted as an alkynyl‐assisted Zr–Zr binding orbital.

^1^H NMR spectrum of **5** in benzene‐*d*_6_ shows two resonances for cyclopentadienyl ligands at 5.28 and 5.27 ppm. The resonance of the methyl group is found at −2.60 ppm, thus exhibiting a remarkable shift to a higher field compared to the value of δ 0.26 ppm in **3**, highlighting the different electronic situation imposed by the second Zr center in **5**. These data suggest a slightly more deshielded situation due to the phenyl substituent compared to the trimethylsilyl substituted complex **F** (*cf*. ^1^H NMR in benzene‐*d*_6_: δ 5.19, 5.17 (Cp), −2.92 ppm (Me)). Crystals of **5** suitable for SC‐XRD analysis were obtained by slow cooling of a toluene/pentane solution from room temperature to −70°C (Figure 2, discussion of structural data).

### 2.2 Reactivity of Complex **5**

#### 2.2.1 Reactivity Toward Amine Boranes and Hydrogen

**Scheme 2:**
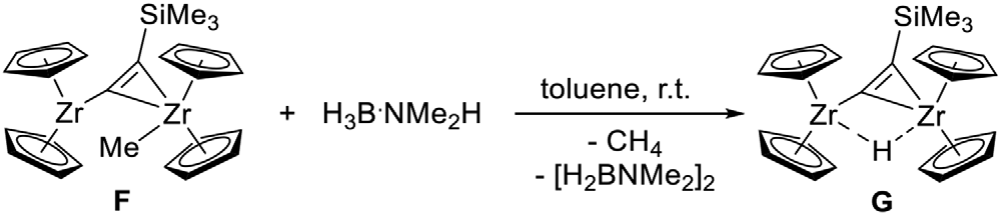
Exemplary reaction of compound **F** (showing Zr(III)/Zr(III) connectivity found by SC‐XRD analysis) with H_3_B·NMe_2_H, producing the dinuclear Zr hydride **G**.^[^ ^13^ ^].^

Trimethylsilyl alkynyl complexes **E** and **F** react with an excess of H_3_B·NMe_2_H by protonation of the CH_2_R (R = SiMe_3_ (**E**), H (**F**)) moiety and formation of SiMe_4_ or CH_4_, respectively, and cyclodiborazane [H_2_BNMe_2_]_2_ (Scheme 2). Hydride transfer leads to the labile hydride‐bridged analogue [(Cp_2_Zr)_2_(*μ*‐H)(*μ*‐C_2_SiMe_3_)] (**G**), a catalytically relevant species for the dehydrocoupling of amine boranes.^[^ ^16^ ^]^ Experiments with partially B‐ and N‐deuterated amine boranes demonstrated that the formation of the Zr hydride predominantly occurs via a B‐H hydride transfer.^[^ ^13^ ^]^ To compare the influence of the alkynyl moiety on the reactivity, the same reaction was performed with complex **5**.

Initial experiments were carried out in Young NMR tubes under strict exclusion of air and moisture. When adding a tenfold excess of H_3_B·NMe_2_H to an orange benzene solution of **5** an intensification of color to red was observed, along with gas evolution, likely hydrogen. Gas release was allowed for 12 h and ^1^H and ^11^B NMR spectra showed, as expected, the formation of cyclodiborazane [H_2_B‐NMe_2_]_2_, a new metallocene species (^1^H NMR in benzene‐*d*_6_: δ 5.84, 5.78 ppm (Cp) and the presence of the starting compound **5**. The addition of further three equivalents of H_3_B·NMe_2_H reduced the amount of **5** and produced a hydride signal at *δ* −5.81 ppm, which is associated with the aforementioned new Cp resonances. These three resonances are in line with those of the trimethylsilyl substituted derivative **G** and reveal a greater deshielding of the hydride in the new complex **6** (cf. species **G**: ^1^H NMR in benzene‐*d*_6_: *δ* 5.77, 5.76 (Cp), −7.22 ppm (*μ*‐H)). The color of the reaction solution changed to turquoise, but all further handling of this reaction mixture, such as drying in vacuo, filtration, or even storage, led to decomposition, indicated by a color change to yellow‐brownish. In all further attempts to repeat this reaction on a larger scale, it was not possible to isolate the turquoise/blue species. Nevertheless, these experiments suggest that complex **5** acts as a precatalyst in the dehydrocoupling of H_3_B∙NMe_2_H, undergoing the same conversion as its trimethylsilyl‐substituted analogue.^[^ ^13^ ^]^

**Figure 3:**
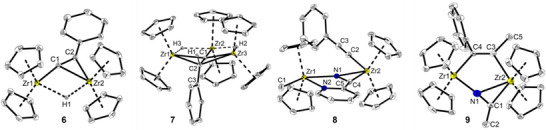
Molecular structures of complexes **6**–**9**. Thermal ellipsoids correspond to 30% probability at 150 K. Hydrogen atoms, except H1 in **6** and H1‐H3 in **7**, as well as the minor occupied disordered Cp orientation in **6** and the solvent molecules (C_6_H_6_) in **7** were omitted for clarity. Selected structural data are depicted in Table 1.

**Scheme 3:**
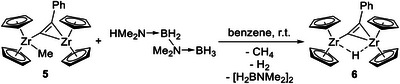
Synthesis of **6** using complex **5** and diborazane H_3_B‐NMe_2_‐BH_2_‐NMe_2_H.

This motivated us to further evaluate methods for isolating this catalytically relevant zirconocene hydride species. In particular, the true nature of these complexes has not yet been determined, although various Lewis structures of related group 4 metallocene were postulated as early as 1997 by Shur and co‐workers.^[^ ^17^ ^]^ The high sensitivity of the postulated zirconocene hydride species **6** requires all experiments to be conducted in sealed NMR tubes. To replace H_3_B∙NMe_2_H as the hydrogen source, hydrogen gas was employed directly, with experiments conducted at different reaction times and temperatures to form the desired hydride species in the highest possible concentration. Using a reaction time of 48 h at 80°C, blue crystals were obtained, which were unfortunately unsuitable for SC‐XRD analysis. However, CI‐MS analysis shows a signal at *m*/*z* 542, clearly identifying this blue species as the hydride [(Cp_2_Zr)_2_(*μ*‐H)(*μ*‐C_2_Ph)] (**6**). Finally, we used linear diborazane H_3_B‐NMe_2_‐BH_2_‐NMe_2_H as the hydrogen source and observed a color change from reddish‐brown to dark blue within 24 h (Scheme 3). SC‐XRD analysis of blue crystals formed from this solution clearly confirmed the structure of dinuclear complex **6** with the assumed bridging hydride ligand (Figure 3, discussion of structural data, see below).

**Scheme 4:**
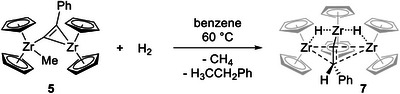
Hydrogenation of complex **5** and formation of **7**.

In all previous experiments, poorly selective reactions were observed, indicated by the presence of several Cp‐containing species in reaction solutions. In particular, reactions with molecular hydrogen showed a set of ^1^H NMR signals that could be assigned to a polynuclear zirconocene hydride species: four low‐field signals with an integral ratio of 5:5:10:10 at *δ* 6.20, 6.02, 5.82 and 5.40 ppm, in line with Cp ligands, as well as a signal at higher field (*δ* −1.45 ppm) with an integral of 2, corresponding to a bridging Zr dihydride. Formation of ethylbenzene, likely by hydrogenation of the bridging phenylalkynyl unit in **5** is corroborated by characteristic ^1^H NMR signals at *δ* 2.44 (*q*, 2H, ^3^ *J* = 7.6 Hz) and 1.08 ppm (*t*, 3H, ^3^ *J* = 7.6 Hz). An additional set of ^1^H NMR signals indicates formation of the known zirconocene hydride dimer [Cp_2_Zr(H)(*μ*‐H)]_2_ (*δ* 5.60 (Cp), 3.70 (*m*, Cp_2_ZrH), −3.60 ppm (*m*, *μ*‐H)).^[^ ^18^ ^]^ Optimization of the reaction conditions, reducing the reaction temperature to 60°C and extending the reaction time to two weeks allowed for the enrichment of the postulated Zr dihydride species (Scheme 4).

Single crystals suitable for SC‐XRD analysis could be obtained from deep purple reaction solutions after slow cooling to ambient temperature (Figure 3, discussion of structural data, see below). The molecular structure confirms the new species **7** as a trinuclear complex in which a formal PhC(H)C unit is positioned between three zirconocene centers that are additionally connected by two Zr‐H bridges. This unexpected structural motif is in excellent agreement with the above described unusual ^1^H NMR pattern and can be understood as an intermediate of complete hydrogenation of the phenylalkynyl group to ethylbenzene. Selective zirconocene hydride‐catalyzed semi‐hydrogenation of terminal alkynes has recently been investigated by the Bayeh–Romero group, using *i*‐PrOH and silanes as proton and hydride source, respectively, instead of molecular hydrogen.^[^ ^19^ ^]^ In this context it might be worth investigating hydrogenation reactions using a broader set of zirconocene hydride species and hydrogen in the future. Furthermore, activation of other poorly‐reactive small molecules like N_2_^[^ ^20^ ^]^ or CO_2_^[^ ^21^ ^]^ will be studied using these and related oligonuclear Zr hydride complexes.

Hydride complexes **6** and **7** are intensely colored, however, due to their high sensitivity it was not possible to record UV–vis spectra of these complexes. Thus, time‐dependent DFT (TD‐DFT) analysis on the PBE0^[^ ^22^ ^]^‐D3^[^ ^23^ ^]^/def2‐TZVP^[^ ^24^ ^]^ level of theory, based on optimized geometries derived from experimental data, was performed to identify the key charge distributions and transfers that are responsible for the intense color of **6** (Tables S16 and S17) and **7** (Tables S18 and S19). For the title compound **5**, an intense absorption is computed at 433 nm which can be interpreted as ligand‐to‐metal charge transfer (LMCT). This mainly involves the donation of electron density from the alkynyl unit to a d orbital at Zr2. The analogous LMCT bands in the hydride complexes show a substantial bathochromic shift to 519 and 514 nm in **6** and 498 nm in **7**, respectively. It can thus be assumed that the presence of small *μ*‐hydride ligands indirectly influences the color of the complexes, without contributing significantly to the HOMO/LUMO responsible for the color. More specifically, a decrease of the Zr–Zr distance from complex **5** to **6** results in more efficient orbital overlap, leading to a reduction of the HOMO‐LUMO gap from 3.68 eV in **5** to 3.35 and 3.23 eV in **6** and **7**, respectively.

#### 2.2.2 Reactivity Toward 2‐Cyanopyridine and Acetonitrile

**Scheme 5:**
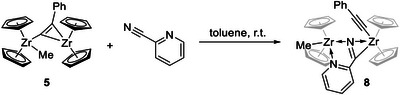
Reaction of complex **5** with 2‐cyanopyridine, producing **8**.

Zirconocene complexes are known to activate small unsaturated molecules such as nitriles and isocyanides in diverse and mostly unpredictable ways.^[^ ^25^ ^]^ Reductive nitrile–nitrile^[^ ^26^ ^]^ C–C coupling or Zr–C insertion and nitrile‐substrate C–C coupling^[^ ^27^ ^]^ reactions are well studied, mostly for group 4 metallocene alkyne complexes. In complex **5**, both the alkyne moiety and the bridging methyl unit could react with nitriles. This species was thus reacted with 2‐cyanopyridine and acetonitrile, two nitriles that showed C–C coupling in previous studies, at the NMR scale in benzene‐*d*_6_. The reaction of **5** with 2‐cyanopyridine at room temperature produced a new zirconocene species, showing two ^1^H NMR signals at *δ* 5.78 and 5.80 ppm, whereas a similar reaction with acetonitrile gave a complex reaction mixture. In both experiments, a considerable amount of precipitate was formed. Although the NMR scale reaction showed promising conversion of **5** and 2‐cyanopyridine, only low yields of 14% of the newly formed compound **8** (Scheme 5) could be isolated on a small preparative scale. Recrystallization of the brown solid from hexane/pentane mixture at low temperatures produced crystals of **8** suitable for SC‐XRD analysis (Figure 3, discussion of structural data, see below). The molecular structure shows a dinuclear zirconocene complex in which the 2‐cyanopyridine connects both Zr centers. The methyl ligand of the starting complex is found on Zr1 and the alkynyl ligand on Zr2, without insertion or coupling to the nitrile substrate. The formation of this complex supports the assumption of a flexible methyl bridging unit in **5**, which can shift between both Zr centers.

**Scheme 6:**
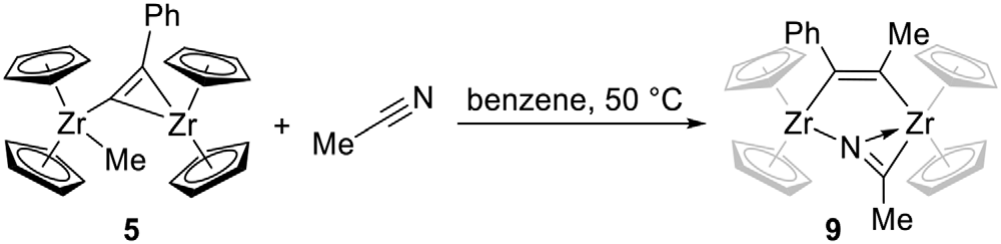
Reaction of complex **5** and acetonitrile, producing **9**.

A slightly better yield could be achieved by reacting two equivalents of acetonitrile and **5** in benzene solution at 50°C for 12 h (Scheme 6). The resulting yellow solid was washed with fresh benzene and isolated, resulting in a yield of 39%. SC‐XRD analysis of yellow single crystals that were isolated from the yellow wash solution reveals complex **9** as a dinuclear complex with bridging acetonitrile. However, in this case, C–C coupling between the alkynyl and methyl ligands produced a dianionic alkenyldiide as a second bridging unit (Figure 3, discussion of structural data, see below). Complex **9** is poorly soluble in most non‐polar solvents, decomposed in acetonitrile and DMSO, and showed moderate solubility in THF that led to slow decomposition (Figure S10).

### 2.3 Discussion of Structure and Bonding Features

#### 2.3.1 General Discussion

Based on the experimental SC‐XRD data of complexes **5**–**9**, characteristic structural changes can be identified. In particular, the C*_α_*–C*_β_* bond and the C*_α_*–C*_β_*–C_Ph_ angle of the (transformed) alkynyl unit (Figure 3; Table 1, columns 1 and 2), which are present in all complexes, undergo the most significant changes as a result of the described reactions.

**Table 1:** Selected experimental and DFT‐optimized (italics) bond lengths, angles, dihedral angles and Zr∙∙∙Zr distances of complexes **5**–**9**.

Complex	C*_α_*‐C*_β_* [Å]^[a]^	C*_α_*‐C*_β_*‐C_Ph_ [°]	Zr‐C*_α_*‐C*_β_*‐Zr [°]^[c]^	Zr∙∙∙Zr [Å]^[b]^
**5**	1.298(6) *1.292*	135.4(4) *137.3*	2.3(15) *12.7*	3.380 *3.390*
**6**	1.297(3) *1.297*	136.7(2) *137.0*	2.8(6) *2.0*	3.323 *3.288*
**7**	1.468(2) *1.457*	130.37(15) *129.7*	158.16(7) *159.4*	3.332 *3.303*
**8**	1.214(2) *1.221*	177.7(2) *174.3*	−43.1 *2.6*	4.499 4.474
**9**	1.334(5)^[d]^ *1.344*	124.2(3) *123.0*	25.5(3) *6.0*	3.583 *3.647*

^[a]^ C–C distance of the alkynyl/former alkynyl unit.

^[b]^ Distances were measured with the programme Diamond. For complex **7** the shortest distance Zr1‐Zr2 is given.

^[c]^ For complex **7** Zr3‐C1‐C2‐Zr1 is depicted.

^[d]^ In complex **9** C*_α_* represents C3 and C*_β_* represents C4 (Figure 3).

The C*_α_*–C*_β_* distance of this unit in **8** is shortest at 1.214(2) Å and can be described as a triple bond (*cf*. d(C≡C) = 1.20 Å^[^ ^28^ ^]^). The nearly linear C*_α_*–C*_β_*–C_Ph_ unit (C*_α_*–C*_β_*–C_Ph_ 177.7(2)°) also indicates that there is almost no *π* interaction between the alkyne unit and the second zirconocene center in **8**. As soon as this unit bridges two Zr centers as found in **5** and **6**, the C*_α_*–C*_β_* distance increases and the C*_α_*–C*_β_*–C_Ph_ angle decreases significantly (Table 1). In **5** and **6** this bond is best described as a shortened double bond (cf. *d*(C─C) = 1.34 Å),^[^ ^28^ ^]^ whereas in complex **7** the long C*_α_*–C*_β_* distance of 1.468(2) Å is close to a single bond (cf. *d*(C─C) = 1.50 Å),^[^ ^28^ ^]^ reflecting the partial hydrogenation of the former alkynyl unit and pointing to a highly unusual coordination environment of C*_α_*.

In this unit of complex **9**, the two carbon atoms C*_α_* and C*_β_* (Figure 3, C3–C4) bridge the two zirconocene centres via Zr–C, *σ* bonds, which is consistent with the double bond‐like C3–C4 distance. In complexes **8** and **9** an uncommon bridging motif is found in which the N of the nitrile connects both Zr centers. The C atom of the nitrile fragments is connected to one Zr as well; this formal (*μ*‐*π*‐N≡CR) coordination leads to a bending of the nitrile unit (**8**: N1‐C4‐C5 119.5(2)°; **9**: N1‐C1‐C2 126.5(2)°) and elongation of the N–C distance (**8**: N1‐C4 1.263(2); **9**: N1‐C1 1.242(5) Å; cf. *d*(N≡C) = 1.14, *d*(N═C) = 1.27 Å).^[^ ^28^ ^]^ These values are well in line with related aryl and alkyl nitrile bridged dizirconium complexes, for example, *d*(N─C) = 1.255(9) Å and N─C─C = 127.4(7)° in [Zr_2_Cl_6_py_2_(*μ*‐NC‐*t*‐Bu)]^[^ ^29^ ^]^ or *d*(N─C) = 1.23(2) Å and N─C─C = 130(2)° in [(Cp_2_Zr)_2_(*μ*‐C_2_Me)(*μ*‐NC‐Tol)].^[^ ^30^ ^]^

In **5** and **6** the Zr centers and the alkynyl unit are found in almost the same plane, reflected by the small Zr‐C*_α_*‐C*_β_*‐Zr dihedral angles. The distance between the Zr atoms in all discussed complexes ranges between 3.323 and 4.499 Å, with the largest distance found in complex **8** and the second largest in **7** for the two opposing Zr atoms (*d*(Zr1∙∙∙Zr3) = 4.329 Å). In all other cases, this distance is shorter than 3.6 Å, in the range of values found in other Zr(III)–Zr(III) complexes such as Zr_2_Cl_6_(dppe)_2_ (3.099 Å^[^ ^31^ ^]^) or (Cp_2_ZrI)_2_ (3.699 Å^[^ ^32^ ^]^). Of note, this is in the range of so called “superlong” Zr─Zr bonds.^[^ ^33^ ^]^ The diamagnetism of these dinuclear compounds was explained using ab initio computations.^[^ ^33^ ^]^ From a synthetic point of view, complex **5** was synthesized by reacting the Zr(IV) complex **3** with a Zr(II) source, which could lead to a similar electronic situation if comproportionation to a Zr(III)‐Zr(III) system takes place. In fact, this was observed for complex **F**.^[^ ^13^ ^]^ However, a closer examination of the Zr–C1 distances in **5** reveals two significantly different bond lengths (Zr1–C1 2.453(5), Zr2–C1 2.580(5) Å), both of which are longer than the reference Zr‐C single bond value (c.f. *d*(Zr–C) = 2.29 Å)^[^ ^28^ ^]^ and the Zr1‐C2 distance of the *σ*‐bonded alkynyl unit (**5**: Zr1‐C2 2.110(4) Å). These data suggest the same mixed‐valent Zr(IV)–Zr(II) electronic situation in the solid state as observed before for **E** (Figure 1c).^[^ ^13^ ^]^

#### 2.3.2 Structural Analysis of Complexes 5 and 6

**Figure 4:**
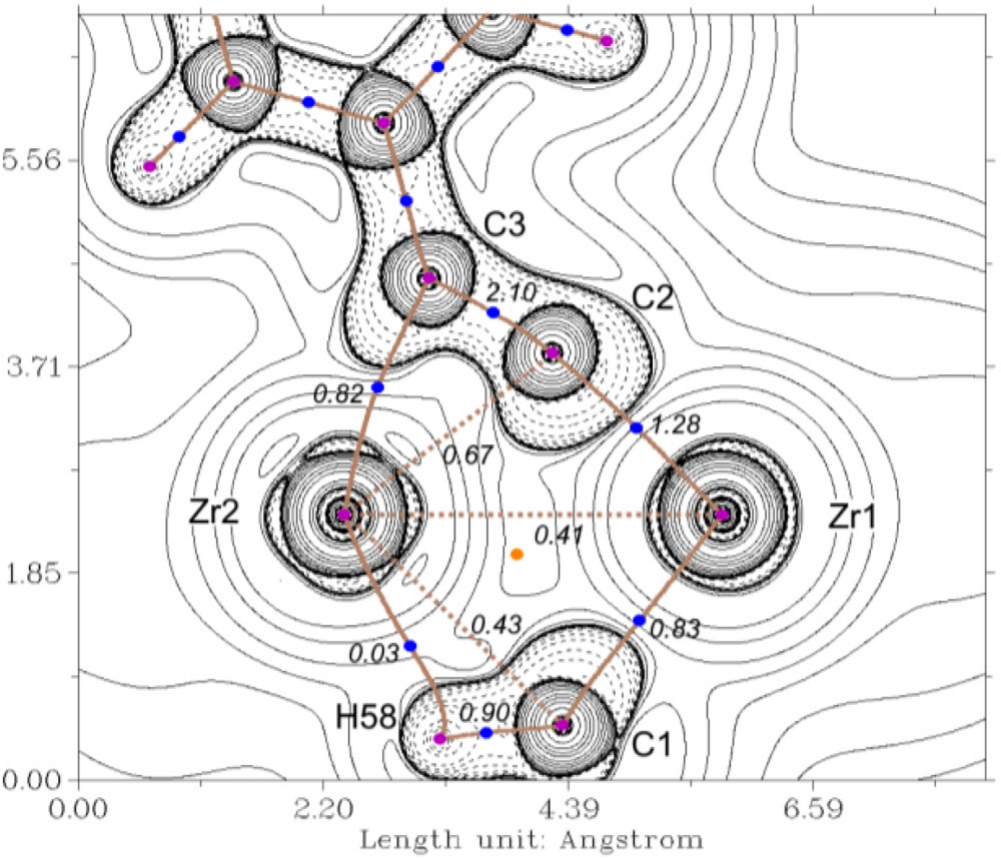
Contour plot of the Laplacian of the electron density *∇* ^2^ *r* of complex **5** in the Zr1‐Zr2‐C3 plane. Dashed lines indicate negative (local charge concentration), solid lines indicate positive values (local charge depletion). The Laplacian plot is overlaid with the molecular graph from QT‐AIM analysis and Wiberg bond indices (small italic numbers). Brown lines indicate bond paths, brown dashed lines are hypothetical bonds, blue dots correspond to bond critical points, and light brown dots indicate ring critical points. Density from B3LYP‐D3/def2‐TZVP calculation.

To further analyze the electronic situations in complexes **5**–**9**, a set of bond analyses, based on gas phase structures on the B3LYP^[^ ^34^ ^]^‐D3^[^ ^23^ ^]^/def2‐TZVP^[^ ^24^ ^]^ level of theory (with Gaussian16^[^ ^35^ ^]^) was performed. A comparison of experimental and calculated structural parameters (Table 1) shows good agreement and reliable structures for further bond analyses. Next, a quantum theory of atoms in molecules (QT‐AIM)^[^ ^36^ ^]^ and natural bond orbital (NBO)^[^ ^37^ ^]^ analysis, as well as inspection of the Wiberg bond indices (WBI) was done. For complex **5**, a combination of the contour plot of the Laplacian of the electron density *∇* ^2^ *r* with the QT‐AIM and WBI analysis (Figure 4) provides insight into the bond situation along the Zr1–C1–Zr2 unit in this complex and shows two distinct bonds between the Zr centers and the C atom of the methyl unit.

While a bond path with a bond critical point and a WBI of 0.83 to Zr1 can be identified, both criteria are missing between Zr2 and C1. The WBI of 0.43 supports the weak interaction in this case. The *η* ^2^‐coordination of the alkynyl unit is represented by the electron density pointing from C2/C3 in the direction of Zr2 even though no bond critical point and path was found between Zr2 and C2 in QT‐AIM. The longer C2‐C3 distance compared to PhC_2_H (Figure S25, C–C 1.202 Å, WBI 2.90) or to **8** (WBI 2.74) is well reflected by the smaller WBI of 2.1 in this unit of complex **5**. A comparison of the shape of the Laplacians of electron density at Zr1 and Zr2 reveals no significant differences, in agreement with its identical calculated natural charges (q_nat.NBO_(Zr1/Zr2) = 1.52). The sum of the natural charges of the zirconocene units (q_nat.NBO_(Cp_2_Zr1/Cp_2_Zr2) = 0.74/0.73) shows only minor differences and the charges of the negatively charged bridging substituents are summed up to q_nat.NBO_(alkynyl) = −1.01 and q_nat.NBO_(CH_3_) = −0.46. These data suggest a well‐balanced Zr(III)‐Zr(III) electronic situation instead of a mixed‐valent Zr(IV)–Zr(II) complex.

As the compound **5** could be unambiguously identified by NMR spectroscopy, the presence of a “superlong” Zr─Zr bond would explain the diamagnetism in this case. An inspection of the natural localized molecular orbitals (NLMOs) shows that NLMO38 can be interpreted as an alkynyl assisted Zr─Zr bond orbital (Figure 2, right) with major contributions from in‐plane d orbitals at Zr1 (14%) and Zr2 (21%) and almost pure p orbitals along the C2‐C3‐C4_Ph_ unit (13%/15%/15%) with a node in the plane between C2 and C3 (Table S4). The Zr–C1 interaction is best described by NLMO44 (Table S4) as a strongly polarized orbital in which the sp^2^ hybrid orbital at C1 has a major contribution (77%) to the Zr1‐methyl *σ* bond, along with minor portions of the Zr2‐methyl interaction (sd^4^‐Zr1: 16, sd^6^‐Zr2: 6%). Weak interactions between the two Zr atoms as well as between C1 and Zr2 are also reflected by lower WBI of 0.41/0.43 (Figure 4) and the absence of bond critical points and paths in QT‐AIM. Alternatively, the connectivity determined by SC‐XRD analysis suggests a Zr(IV)–Zr(II) electronic situation, which seems to be different in solution. For the analogous trimethylsilyl‐alkynyl complex **F** a low‐lying barrier was found for the methyl group shift between the Zr centers (Δ*G* ^#^ = 5.4 kJ/mol). In this case, the Zr(III)–Zr(III) geometry is slightly preferred (*Δ*_R_ *G^ϴ^* = −0.6 kJ/mol) and found by SC‐XRD analysis as well. In the phenyl derivative **5** also the thermodynamically preferred Zr(IV)–Zr(II) isomer (Δ_R_ *G^ϴ^* = 1.6 kJ/mol) is obtained in the solid‐state structure. A barrier of Δ*G* ^#^ = 5.2 kJ/mol suggests facile conversion into the Zr(III)/Zr(III) isomer in solution. These calculated values are significantly lower than the experimentally determined barriers of the alkynyl group shift, referred to as automerization, in **B** and related complexes, which range between 52.7 and 61.9 kJ/mol.^[^ ^11a^ ^]^ The low barriers for the methyl group shift reflect its reactivity, explaining the facile transformation of this unit in the reactions shown here. We suggest that—supported by DFT computations—the thermodynamically favored electronic situation can be tuned by the choice of the substituent at the bridging alkynyl unit.

Based on these considerations, it can be assumed that similar dynamics play a role in complexes **6**–**9**, which were synthesized from **5**. Indeed, ^1^H and ^13^C NMR signals of the phenyl groups indicate free rotation of these groups in complexes **6**–**9**. For simplicity, the following bond analyses were conducted only on the isomers that were determined experimentally by SC‐XRD. In complex **6**, the QT‐AIM parameters are mostly in line with those of **5** (Figure S15). The hydride ligand shows a natural charge of −0.3 and is computed to be positioned almost equidistantly to the Zr centers (Zr1‐H1 2.017, Zr2‐H1 2.043 Å), supported by the WBIs of the Zr─H bonds of 0.45 and 0.47, respectively. NLMO96 reveals almost equal contributions of both Zr atom orbitals (Table S6, NLMO96: 19% Zr1 sd^5^ hybrid; Zr2 17% sd^6^ hybrid; 63% H1 s orbital), allowing for a description as a classical three centre‐two electron [Zr–H–Zr] bridging unit (Figures S15 and S28). It is also noteworthy that the WBI of the Zr–Zr interaction is 0.63, corresponding to the shortened Zr–Zr distance observed in this compound.

#### 2.3.3 Structural Analysis of the Hydrogenation Product 7

The bond analysis of the two bridging hydrides in the trinuclear complex **7** is very similar to that in **6**, showing natural charges of −0.3. Bridging [Zr–H–Zr] units are, in this case, slightly polarized with the zirconocenes coordinated side‐on to the C_2_ fragment contributing slightly more (Table S8, NLMO150, 151). The charge distributions of the intrinsic bond orbitals (IBOs)^[^ ^38^ ^]^ for these two bonds show a similar picture (Table S8, comparison of NLMOs and IBOs). Of note, the central carbon atom C1 is only located 0.352 Å (computed: *0.328* Å) out of the plane defined by the three Zr atoms, close to planar coordinated with an angle between the planes C1‐Zr1‐Zr2 and C1‐Zr3‐Zr2 of 21.8° (computed: *20.2*°). This C atom is significantly closer to the end‐on coordinated Zr2 atom than to the two side‐on coordinated metals Zr1 and Zr3 (C1‐Zr2 2.144(2)/*2.134*; C1‐Zr1 2.235(2)/*2.219*; C1‐Zr3 2.232(2)/*2.220* Å). C2 is found at a significantly greater distance from the side‐on Zr atoms but remains within the range of metal‐carbon bonds (C2‐Zr1 2.464(2)/*2.466*; C2‐Zr3 2.471(2)/*2.471* Å) that is also found in complex **5** (Figure 2, Zr1‐C1 2.453(5), Zr2‐C1 2.580(5) Å). However, for C2‐Zr1 and C2‐Zr3 no bond paths or bond critical points could be found in QT‐AIM analysis, although the WBI is 0.5 for both contacts, similar to Zr‐H hydride bonds (Figure S17). When evaluating this unusual structural feature using an interaction region indicator^[^ ^39^ ^]^ (IRI) analysis (Table S9) a clear binding interaction is found between C2, Zr1, and Zr3. Furthermore, this analysis shows large areas of attractive van der Waals interactions between the Cp ligands and additionally between Cp and the phenyl groups, thus stabilizing this structural feature inside the pocket formed by the three zirconocenes. Planar tetra‐coordinate carbon environments have fascinated molecular chemists for decades.^[^ ^40^ ^]^ While this structural feature is rather unusual, its stabilization in the coordination sphere of transition metals has been reported on several occasions.^[^ ^41^ ^]^

**Figure 5:**
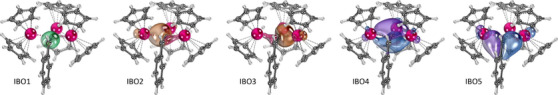
Plot of selected IBOs, describing the bond situation in the trinuclear complex **7**. Atoms/charges: IBO1: C1/0.946, C2/0.993, Zr1/0.027, Zr3/0.028; IBO2: C1/1.764, Zr1/0.090, Zr2/0.090; IBO3: C1/1.763, Zr3/0.090, Zr2/0.089; IBO4: C1/1.746, Zr2/0.057, C2/0.037, Zr1/0.036, Zr3/0.035, H1/0.021; IBO5: C2/1.599, C3/0.133, Zr3/0.077, Zr1/0.075.

Although it does not provide a formal hybridization composition, the IBO analysis gives a more balanced description for this fragment than the NBO/NLMO analysis (Table S8 and Figure 5). IBO1 represents a classical C–C *σ* bond orbital, likely a sp^2^ hybrid. IBO2 and IBO3 can be interpreted as three center‐two electron bond orbitals in the plane connecting sp^2^ hybrid orbitals at C1 with all three Zr centers with strong polarization to C1, reflected by the high charges at C1 (Figure 5) that agree with the corresponding NLMOs (Table S8). IBO4 is orthogonal to IBOs 2 and 3, mainly consisting of a p orbital at C1, which connects all three metal centers and C2 in *π* geometry, only showing minor contributions/charges. This IBO4 can formally be interpreted as a five center‐two electron bond interaction and, due to its *π* type C*_α_*–C*_β_* bond contributions, provides an explanation for the slightly shortened single bond, supported by a WBI of 1.36 for this bond. IBO5 is mainly composed of a p orbital at C2 that lies in the plane defined by the three Zr atoms and connects both side‐on coordinated Zr centers. In contrast to IBO analysis, the NBO analysis found a triply bonded C1 atom to Zr2 with occupancies below 1.6 electrons in two of these NBOs. This again points to a delocalized electronic system, which is well reflected in the IBO analysis. The large natural charge *q*_nat.NBO_(C*_α_*–C*_β_*) of −1.57 allows for an interpretation of this fragment as a tetraanionic ligand, which is in line with the five IBOs and NLMOs describing the C1–C2 unit and its binding contributions to the Zr centers (Figure 5 and Table S8). Assuming that **7** is composed of three Zr(IV) centers with six monoanionic Cp and two hydride ligands, formally, four negative charges should be located on the hydrocarbon bridging unit. In line with this the side‐on coordinated zirconocene fragments show higher natural charges q_nat.NBO_(Cp_2_Zr1 0.77; Cp_2_Zr3 0.76) compared to the metallocene *σ* coordinated to the terminal C atom (Cp_2_Zr2 0.64), compensated by the negative charges of its hydride ligands.

#### 2.3.4 Structural Analysis of Nitrile Insertion Products 8 and 9

**Figure 6:**
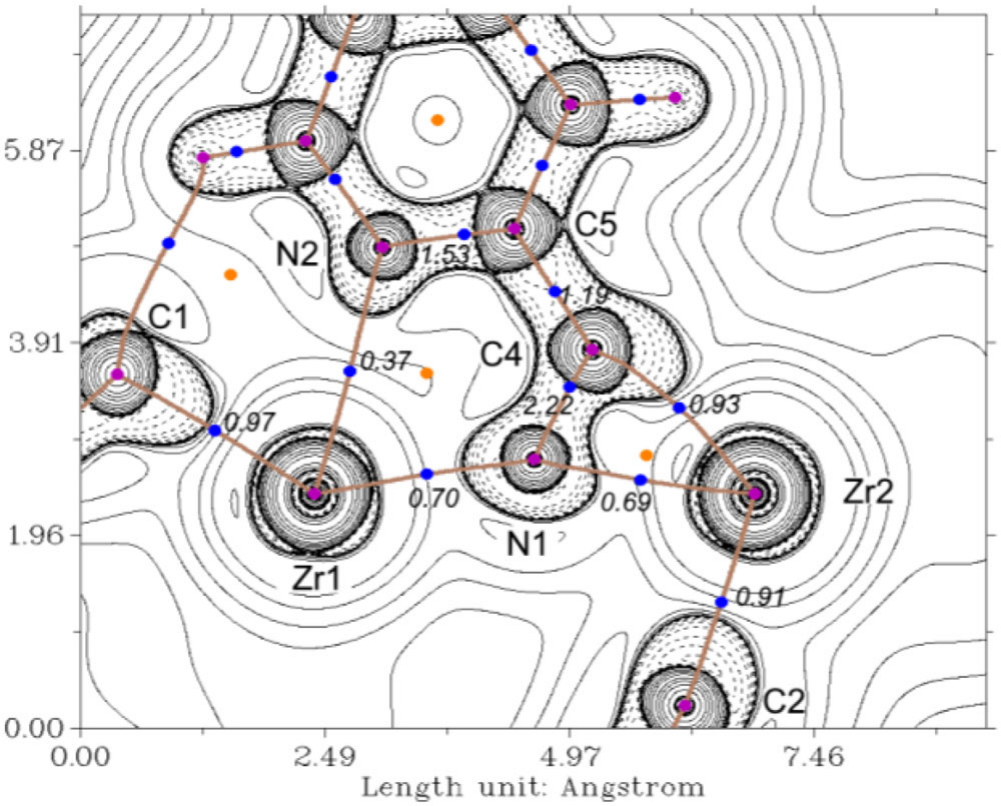
Contour plot of the Laplacian of the electron density ∇^2^ *r* of complex **8** in the Zr1‐Zr2‐N2 plane. Dashed lines indicate negative (local charge concentration) and solid lines indicate positive values (local charge depletion). The Laplacian plot is overlaid with the molecular graph from QT‐AIM analysis and Wiberg bond indices (small italic numbers). Brown lines indicate bond paths, blue dots correspond to bond critical points, and light brown dots indicate ring critical points. Density from B3LYP‐D3/def2‐TZVP calculation.

The 2‐cyanopyridine complex **8** shows the former methyl and alkynyl units of the starting complex **5**, now coordinated to a different Zr center. The 2‐cyanopyridine substrate is positioned between both metal centers and forms three‐ (Zr2‐N1‐C4) and five‐membered ring systems (Zr1‐N1‐C4‐C5‐N2) that show an angle of about 6° between the two planes (Figure 6). A QT‐AIM analysis reveals the presence of ring critical points at the centers of these metallacycles (Figure 6). Coordination of 2‐cyanopyridine is associated with a formal reduction, as evidenced by the significant bending of the nitrile unit and a computed natural charge of ‐0.81. The N atom of the nitrile unit formally acts as a bridging element between the two Zr centers. Both the contour plot of the Laplacian of the electron density (Figure 6) and the NBO analysis indicate two lone pairs on the central N1 atom (Table S11, NLMO52 and NLMO53). Elongation of the former nitrile group is evidenced by its experimental bond length (C4‐N1 1.263(2) Å), which is in line with a C═N double bond and supported by the WBI of 2.2. NLMO analysis shows that this bond can be described as a classical C–N *π* bond which shows only minor contributions of Zr2 and C5 orbitals (NLMO65). The interaction of the former nitrile carbon C4 and Zr2 can be understood as a polarized *σ* bond with major contributions from the C atom (Table S11, NLMO62 73% C4 sp^2^ hybrid; 22% Zr1 sd^14^ hybrid).

Finally, in the acetonitrile complex **9** formation of a new C─C bond between the alkynyl and methyl ligands present in the precursor **5** yields the formally dianionic bridging alkenyldiide unit. This description is well in line with the experimentally observed C3–C4 double bond, its WBI of 1.92 and the Laplacians of the electron density pointing from both C to the Zr center (Figure S24). NLMO analysis shows contributions of classical *σ* and *π* C─C bond orbitals as well as two polarized Zr─C *σ* bonds (Table S13, NLMO55, NLMO56; NLMO60; and NLMO62). Similarly as in **8**, the bridging acetonitrile is reduced, evidenced by its bending, a *q*_nat.NBO_ value of −0.8, and the five NLMOs that describe the bond situation in the CN unit and its connectivity to both Zr centers (Table S13).

## 3 Conclusion

This study highlights the potential of a formally mixed‐valent dinuclear zirconocene complex that can be generated from a Zr(IV) alkynyl complex and a Zr(II) source for a variety of bond activation reactions. Detailed experimental and computational studies reveal unique structural and bonding features in the title compound [(Cp_2_Zr)_2_(*μ*‐Me)(*μ*‐C_2_Ph)] with a highly fluxional bridging methyl group that allows for a wide range of unusual reactivity. Hydrogenation of this species using amine boranes or molecular hydrogen furnishes rare examples of highly sensitive di‐ and trinuclear zirconocene hydride complexes that are relevant in stoichiometric and catalytic (de)hydrogenation reactions. The complex formed in the latter case possesses the unusual structural feature of a planar tetracoordinate carbon atom, surrounded by three Zr centers that are connected through three‐center two‐electron‐bonded hydrides and a side‐on interaction with the formally tetraanionic former alkynyl unit. In reactions with nitriles, the formation of rare examples of nitrile coordination to the Zr center and reduction of the substrate without further coupling can be observed.

The modular protocol used for the preparation of the title compound suggests the facile extension of this structural motif to other, related complexes that allows for a more detailed study of structure‐activity relationships. Thus, the experimental and theoretical results presented herein form the basis for further systematic applications of such mixed‐valent homo‐ and heterobimetallic early transition metal complexes for stoichiometric and catalytic bond activation reactions.

## Supporting Information

The authors have cited additional references within the Supporting Information.^[^ ^42^ ^]^

## Supporting information



Supporting Information
