# Supplementary material for: The Dinuclear Zirconocene Complex [(Cp2Zr)2(μ‐Me)(μ‐C2Ph)] as a Platform for Small Molecule Activation
Source: Chemistry. 2025 Apr 7;31(26):e202500857. doi: 10.1002/chem.202500857 (PMC12063046; doi:10.1002/chem.202500857)
Supplement: Supplementary file 1 — Supporting Information [file CHEM-31-e202500857-s001.docx]

Supporting Information

The Dinuclear Zirconocene Complex
(Cp_2_Zr)_2_(µ-Me)(µ-C≡CPh) as a Platform for
Small Molecule Activation

Hanan Al Hamwi^a^, Mirko Rippke^a^, Kevin Lindenau^a^, Anke Spannenberg^a^, Martin Lamač,^b^ Fabian Reiß*^a^ and Torsten Beweries*^a^

^a^ Leibniz-Institut für Katalyse e.V., Albert-Einstein-Str. 29a, 18059 Rostock, Germany

Email: [fabian.reiss@catalysis.de](mailto:fabian.reiss@catalysis.de), torsten.beweries@catalysis.de

^b^ J. Heyrovsky Institute of Physicalm Chemistry of the Czech Academy of Sciences, Dolejškova 2155/3, 182 23 Prague 8, Czech Republic


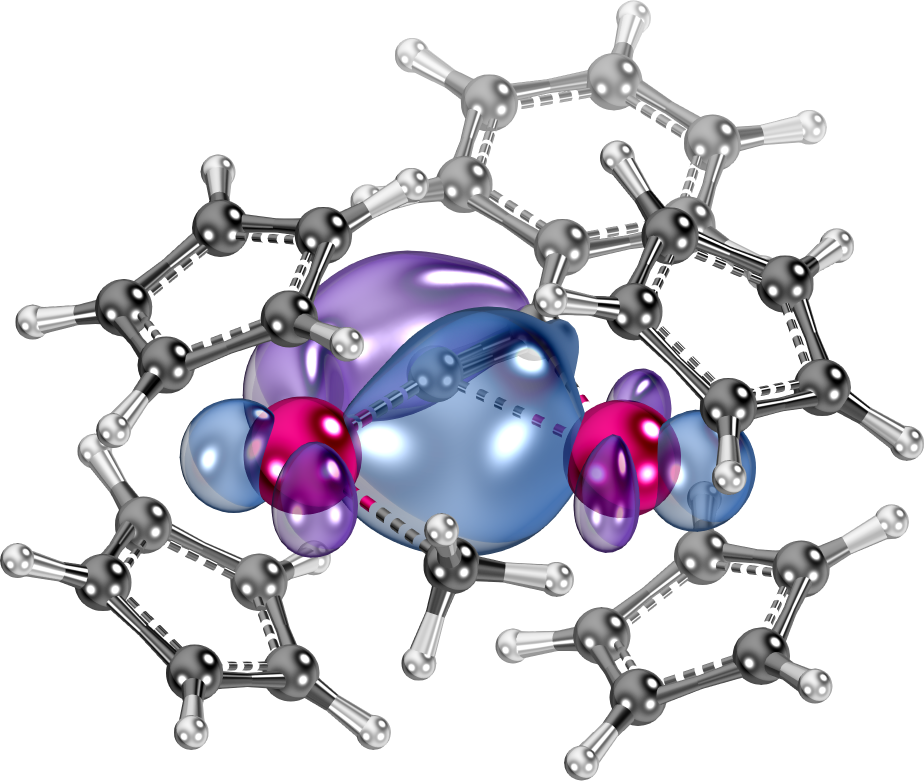


**Table of Contents**

[1 Experimental details 2](#_Toc184375757)

[2 Structure elucidation 3](#_Toc184375758)

[3 Syntheses of compounds 5](#_Toc184375759)

[4 Computational Details 19](#_Toc184375760)

[5 References 79](#_Toc184375761)

# Experimental details

## General information

All manipulations were carried out in an oxygen- and moisture-free argon atmosphere using standard Schlenk and drybox techniques. The solvents were purified with the Grubbs-type column system “Pure Solv MD-5” and dispensed into thick-walled glass Schlenk bombs equipped with Young-type Teflon valve stopcocks. Cp_2_ZrCl_2_ (Aldrich, 98%) was stored under argon and used as received. Cp_2_ZrMe_2_^[[1]](#endnote-1)^, Cp_2_ZrClMe^[[2]](#endnote-2)^ and Cp_2_Zr(py)(η^2^-Me_3_SiC_2_SiMe_3_)^[[3]](#endnote-3)^ were prepared according to literature procedures. *n*-BuLi was used as a 2.5 M solution in *n*-hexane (Sigma-Aldrich). Acetonitrile (Roth, 99.8%) was stored after drying under argon with molecular sieves. The gases argon (Ar) (Linde, 99.999%) and hydrogen (H_2_) (Alphagaz, 99.999%) were used without further purification or drying. Phenylacetylene (PhC_2_H) (Sigma-Aldrich, 98%) and 2-cyanopyridine (Sigma-Aldrich, 98%) were distilled under reduced pressure, and stored under Ar over molecular sieves prior to use.

**NMR spectra** were determined on Bruker AV300 and AV400 instruments. ^1^H and ^13^C{^1^H} chemical shifts were referenced to the solvent signal: benzene-*d*_6_ (*δ*_H_ 7.16 ppm, *δ*_C_ 128.06 ppm).^[[4]](#endnote-4)^

**IR spectra** were recorded on a Bruker Alpha FT-IR ATR spectrometer, placed in a glove box under Ar atmosphere. Spectra are not corrected.

**MS analysis** was done using a Finnigan MAT 95-XP instrument (Thermo-Electron) in CI^+^/CI^-^ mode (isobutene).

**Melting points** are uncorrected and were determined in sealed capillaries under Ar atmosphere using a Mettler-Toledo MP 70.

**X-Ray diffraction data** were collected on a STOE IPDS II (**8**) and a Bruker Kappa APEX II Duo diffractometer (**5, 6, 7** and **9**), respectively. The structures were solved by direct methods (SHELXS-97)^[[5]](#endnote-5)^ and refined by full-matrix least-squares procedures on *F*^2^ (SHELXL-2018).^[[6]](#endnote-6)^ Diamond^[[7]](#endnote-7)^ was used for graphical representations.

**CHN analysis** was using a Leco TruSpec elemental analyser. At this point it should be pointed out that we could not obtain satisfactory elemental analysis in most cases. Despite repeated recrystallisation, repeated measurements with and without oxidiser V_2_O_5_ and modified furnace temperature, we observed up to 20% less carbon content than calculated/expected. This behaviour might be explained by formation of mixed zirconium-silicon-carbides (ceramics) in the furnace and therefore the carbon content dramatically decreases.^[[8]](#endnote-8)^

**All DFT calculations** were carried out with the Gaussian 16^[[9]](#endnote-9)^ package of molecular orbital programmes. Further details are given in the computational details section.

# Crystallographic details

**Table S1.** Crystallographic details of complexes **5**, **6** and **7**.

| Compound | **5** | **6** | **7** |
| --- | --- | --- | --- |
| Chem. Formula | C_29_H_28_Zr_2_ | C_28_H_26_Zr_2_ | C_47_H_47_Zr_3_ |
| Formula weight [g/mol] | 558.95 | 544.93 | 885.50 |
| Colour | yellow | brown | violet |
| Crystal system | monoclinic | monoclinic | monoclinic |
| Space group | *P*2_1_ | *C*2/*c* | *P*2_1_/*n* |
| *a* [Å] | 8.1794(1) | 28.1589(17) | 15.3428(6) |
| *b* [Å] | 8.5244(1) | 8.0876(5) | 10.6889(4) |
| *c* [Å] | 16.3403(2) | 19.5397(11) | 23.8412(10) |
| *α* [°] | 90 | 90 | 90 |
| *β* [°] | 94.5563(6) | 92.8725(11) | 104.2478(6) |
| *γ* [°] | 90 | 90 | 90 |
| *V* [Å^3^] | 1135.72(2) | 4444.3(5) | 3789.6(3) |
| Z | 2 | 8 | 4 |
| *ρ*_calcd._ [g/cm^3^] | 1.634 | 1.629 | 1.552 |
| *μ* [mm^−1^] | 7.612 | 0.950 | 0.842 |
| *T* [K] | 170(2) | 150(2) | 150(2) |
| Radiation type | CuKα | MoKα | MoKα |
| Measured reflections | 13232 | 43962 | 42764 |
| Independent reflections | 4001 | 6031 | 8282 |
| Reflections with *I* > 2*σ*(*I*) | 3938 | 5227 | 7354 |
| *R*_int_ | 0.0289 | 0.0345 | 0.0319 |
| *F*(000) | 564 | 2192 | 1796 |
| *R*_1_(*R*[*F*^2^>2*σ*(*F*^2^)]) | 0.0209 | 0.0267 | 0.0223 |
| w*R*_2_(*F*^2^) | 0.0528 | 0.0639 | 0.0550 |
| GooF | 1.044 | 1.028 | 1.088 |
| No. of Parameters | 292 | 297 | 463 |
| CCDC | 2402406 | 2402407 | 2402408 |

**Table S2.** Crystallographic details of complexes **8** and **9**.

| Compound | **8** | **9** |
| --- | --- | --- |
| Chem. Formula | C_35_H_32_N_2_Zr_2_ | C_31_H_31_NZr_2_ |
| Formula weight [g/mol] | 663.06 | 600.01 |
| Colour | orange | yellow |
| Crystal system | monoclinic | orthorhombic |
| Space group | *P*2_1_/*c* | *Pna*2_1_ |
| *a* [Å] | 8.2341(2) | 15.6156(11) |
| *b* [Å] | 17.6022(3) | 9.1576(7) |
| *c* [Å] | 20.0651(5) | 17.7148(13) |
| *α* [°] | 90 | 90 |
| *β* [°] | 97.865(2) | 90 |
| *γ* [°] | 90 | 90 |
| *V* [Å^3^] | 2880(11) | 2533.2(3) |
| Z | 4 | 4 |
| *ρ*_calcd._ [g/cm^3^] | 1.529 | 1.573 |
| *μ* [mm^−1^] | 0.750 | 0.842 |
| *T* [K] | 150(2) | 150(2) |
| Radiation type | MoKα | MoKα |
| Measured reflections | 44164 | 49783 |
| Independent reflections | 6942 | 6747 |
| Reflections with *I* > 2*σ*(*I*) | 6284 | 6438 |
| *R*_int_ | 0.0142 | 0.0337 |
| *F*(000) | 1344 | 1216 |
| *R*_1_(*R*[*F*^2^>2*σ*(*F*^2^)]) | 0.0214 | 0.0230 |
| w*R*_2_(*F*^2^) | 0.0600 | 0.0533 |
| GooF | 1.037 | 1.045 |
| No. of Parameters | 353 | 309 |
| CCDC # | 2402409 | 2402410 |

# Synthesis of compounds

## Synthesis of PhC_2_Li (1)

A clear solution of HC≡CPh (500 mg, 4.90 mmol) in 100 mL diethyl ether was cooled to -78°C. Then *n*-butyllithium (2.0 mL, 2.5 M in *n*-hexane, 4.90 mmol) was added dropwise over ten minutes. The solution was stirred in a cold bath for one hour and then brought to room temperature overnight. All volatile components were removed under vacuum and the remaining solid was washed with *n*-hexane. After removal of all volatile components under vacuum, the product PhC_2_Li was obtained as a white solid (343 mg, 65 %).

**^1^H NMR** (300.13 Hz, THF-*d*_8_, 297 K): δ 7.26 (d, 2 H, *o*-Ph), 7.08 (t, 2 H, *m*-Ph), 6.99 ppm (t, 1 H, *p*-Ph).

## Synthesis of Cp_2_Zr(Me)C_2_Ph (3)

The synthesis was performed according to a modified procedure reported by ERKER *et al*.^[^^[[10]](#endnote-10)]^ Cp_2_Zr(Me)Cl (**2**) (0.806 g, 2.960 mmol) and PhC_2_Li (**1**) (0.320 g, 2.960 mmol) were dissolved in 100 mL of toluene. The white suspension was stirred and allowed to warm from -78 °C to room temperature within 24 hours. The suspension was canula-filtered, and all volatile components were removed from the filtrate under vacuum, yielding in an orange oil. The orange oil was dissolved in 30 mL *n*-hexane at room temperature and the solution was placed in an ethanol bath at -78 °C until precipitation occurred. The supernatant was removed using a cannula filtration, and the solid obtained was washed with cold *n*-hexane. Removal of all volatile components under vacuum (4·10^-2^ mbar) yielded a red oil.

*Caution: After a series of attempts to purify complex* ***3*** *using different procedures, this procedure turned out to yield the purest fractions. However, we note that we were not able to avoid smaller impurities of so far unidentified Cp-containing compounds and therefore abstain from CHN analysis of this complex (see* ***Figure S1****).*

**Yield**: 0.898 g (2.661 mmol, 93%).

**^1^H NMR** (400.13 Hz, C_6_D_6_, 297 K): δ 7.52 (m, 2 H, *o*-Ph), 7.02 (m, 2 H, *m-*Ph), 6.96 (m, 1 H, *p*-Ph), 5.93 (s, 10 H, Cp), 0.26 ppm (s, 3 H, Me). **^13^C{^1^H} NMR** (75.48 Hz, C_6_D_6_, 297 K): δ 139.4 (Zr*C*CPh), 131.5 (*o*-Ph), 128.4 (*m*-Ph), 127.1 (*p*-Ph), 125.9 (*i*-Ph), 121.8 (ZrC*C*Ph), 110.8 (*Cp*), 37.2 ppm (*Me*). **IR** (ATR, neat, cm^-1^): 3938 (w), 3074 (w), 2918 (w), 2863 (w), 2777 (w), 2724 (w), 2428 (w), 2279 (w), 2073 (w), 1951 (w), 1808 (w), 1718 (w), 1592 (w), 1568 (w), 1483 (m), 1439 (m), 1405 (w), 1363 (w), 1235 (w), 1202 (m), 1174 (w), 1156 (w), 1124 (w), 1067 (w), 1013 (s), 911 (w), 794 (s), 754 (s), 691 (s), 660 (m), 614 (m), 539 (s), 490 (m), 445 (m). **MS** (CI^+^, *isobutane*): *m*/*z* (%) 235 (100) [Cp_2_ZrMe]^+^, 337 (5.68) [M]^+^, 557 (34) [(Cp_2_Zr)_2_CCPhMe].

**Figure S1.** ^1^H NMR spectrum of the isolated complex **3** (400.13 MHz, C_6_D_6_, 297 K). The asterisk marks unidentified Cp containing impurities.

**Figure S2.** ^13^C{^1^H} NMR spectrum of the isolated complex **3** (75.48 MHz, C_6_D_6_, 297 K). The asterisk marks unidentified Cp containing impurities.

## Synthesis of (Cp_2_Zr)_2_(µ-Me)(µ-C_2_Ph) (5)

Cp_2_Zr(py)(η^2^-Me_3_SiC_2_SiMe_3_) (**4**) (0.422 g, 0.913 mmol) and Cp_2_Zr(Me)(C_2_Ph) (**3**) (0.308 g, 0.913 mmol) were dissolved in 10 mL of benzene and the reaction mixture was stirred for 24 h at 60°C. All volatile components were removed under vacuum and the remaining solid was washed with small amounts of diethyl ether (3×5 mL). Removal of all volatile components under vacuum yielded complex **5** as an orange solid. Crystals suitable for single-crystal X-ray diffraction analysis were obtained by slow cooling of a toluene/pentane solution from room temperature to -78°C.

**Yield**: 0.390 g (0.638 mmol, 77%).

**^1^H** **NMR** (300.20 MHz, C_6_D_6_, 297 K): δ 7.77-7.73 (m, 2 H, *o*-Ph), 7.44 (m, 2 H, *m*-Ph), 7.22 (m, 1 H, *p*-Ph), 5.28 (s, 10 H, Cp), 5.27 (s, 10 H, Cp), −2.60 ppm (s, 3 H, Me). **^13^C{^1^H} NMR** (100.63 Hz, C_6_D_6_, 297 K): δ 247.2 (Zr*C*CPh), 186.5 (ZrC*C*Ph), 130.9 (Ph), 128.8 (Ph), 126.6 (Ph), 105.4 (Cp), 103.9 (Cp), 15.6 ppm (Me) **MS** (CI^+^, isobutane): *m*/*z* (%) 558 (100) [Cp_2_Zr(CCPh)Cp_2_ZrMe]^+^, 337 (21)[M−Cp_2_Zr]^+^, 321 (14) [Cp_2_ZrCCPh]^+^, 239 (59) [Cp_2_ZrMe]^+^, 102 (6) [CCPh]^+^, 67 (43) [Cp]^+^. **Mp**.: 230 °C (decomp.). **CHN analysis** calc. for C_34_H_47_Zr_2_: C, 63.99; H, 7.42. Found: C, 54.17; H, 3.494. **IR** (ATR, neat, cm^-1^): 3072 (w), 2914 (w), 2085 (w), 1833 (w), 1739 (w), 1689 (w), 1589 (w), 1570 (w), 1481 (w), 1438 (w), 1363 (w), 1306 (w), 1242 (w), 1206 (w), 1155 (w), 1124 (w), 1068 (w), 1013 (m), 843 (w), 788 (s), 754 (s), 693 (m), 659 (m), 613 (w), 541 (w), 474 (w), 419 (w).

**Figure S3.** ^1^H NMR spectrum of complex **5** (300.2 MHz, C_6_D_6_, 297 K).

**Figure S4.** ^13^C{^1^H} NMR spectrum of complex **5** (100.63 MHz, C_6_D_6_, 297 K).

## Synthesis of (Cp_2_Zr)_2_(µ-H)(µ-C_2_Ph) (6)

**Procedure 1:**

(Cp_2_Zr)_2_(µ-Me)(µ-C_2_Ph) (**5**) (25 mg, NMR scale) were dissolved in 0.6 mL of benzene and degassed three times using the freeze-pump-thaw procedure. The NMR tube was filled with H_2_ and the reaction mixture was kept in an oil bath at 80°C for 48 h. Blue crystals were obtained. These were removed from the tube in the glove box without drying steps and directly placed in a vial for CI-MS analysis.

**Procedure 2:**

(Cp_2_Zr)_2_(µ-Me)(µ-C_2_Ph) (**5**) (20 mg, 0.035 mmol, NMR scale) and diborazane H_3_B-NMe_2_-BH_2_-NMe_2_H (25 mg, 0.215 mmol) were dissolved in 0.6 mL of benzene. The NMR tube was stored without cap in the glove box for 4 h. After 24 h the colour of the solution changed from red brown to dark blue. After 48 h blue crystals of complex **6** suitable for single crystal X-ray diffraction analysis were obtained.

*Notes: The same reactivity was investigated using dimethylamine borane (H_3_B·NMe_2_H) instead of the linear diborazane. Similar reactivity was observed, but this reaction did not lead into the deposition of suitable single crystals.*

*The blue crystals decompose when dried in high vacuum or in an Ar stream and decolourise to yield a yellow solid which is poorly soluble and whose identity has unfortunately not yet been clarified.*

**^1^H NMR** (400.13 MHz, C_6_D_6_, 297 K): δ 7.28-7.24 (m, 2 H, *o*-Ph), 7.12-7.05 (m, 2 H, *m*-Ph), 7.06 (m, 1 H, *p*-Ph), 5.84 (s, 10 H, Cp), 5.78 (s, 10 H, Cp), −5.81 ppm (s, 1 H, H). **^13^C{^1^H}** **NMR** (100.63 Hz, C_6_D_6_, 297 K): δ 274.65 (Zr-*C*CPh), 195.55 (Zr-C*C*Ph), 142.06 (Ph), 130.26 (Ph), 126.47 (Ph), 105.29 (Cp), 105.25 (Cp). **MS** (CI^+^, isobutane): *m*/*z* (%) 542 (100) [Cp_2_Zr(C_2_Ph)Cp_2_ZrH]. **IR** (ATR, neat, cm^-1^): 3081 (w), 2919 (w), 2849 (w), 2073 (w), 1832 (w), 1665 (w), 1589 (w), 1568 (w), 1479 (w), 1435 (w), 1360 (w), 1304 (w), 1234 (w), 1151 (w), 1122 (w), 1055 (w), 1012 (w), 983 (w), 922 (w), 899 (w), 823 (w), 783 (m), 754 (m), 690 (w), 678 (w), 654 (w), 612 (w), 547 (w), 463 (w), 414 (w).

**Figure S5.** ^1^H NMR spectrum of complex **6** (400.13 MHz, C_6_D_6_, 297 K). Identified impurities: # complex **5**, + [Me_2_NBH_2_]_2_, * grease.

**Figure S6.** ^13^C{^1^H} NMR spectrum of complex **6** (100.63 MHz, C_6_D_6_, 297 K). Identified impurities: # complex **5**, + [Me_2_NBH_2_]_2_.

## Synthesis of (Cp_2_Zr)_3_(µ-H) _2_(σ-µ-C=CHPh) (7)

(Cp_2_Zr)_2_(µ-Me)(µ-C_2_Ph) (**5**) (25 mg, NMR scale) was dissolved in 0.6 mL of benzene and degassed three times using the freeze-pump-thaw procedure. The NMR tube was filled with H_2_ and the reaction mixture was kept in an oil bath at 60°C for two weeks. Purple crystals suitable for single-crystal X-ray diffraction analysis were obtained while slowly cooling this solution to ambient temperature and storing for prolonged time.

*Note: The purple crystals decompose during drying in high vacuum or with an Ar stream and decolourise to yield a yellow solid. Due to this high instability of the compound, the best NMR results could be obtained directly from the reaction mixture. This also allowed us to identify further reaction products. The reported NMR resonances were assigned using ^1^H-^1^H NOESY, ^1^H-^13^C HSQC and ^1^H-^13^C HMBC spectra and support the geometry of complex* ***7*** *determined by SC-XRD analysis.*

**^1^H NMR** (400.13 MHz, C_6_D_6_, 297 K): δ 7.11-7.09 (m, 2 H, *m*-Ph), 6.69-7.67 (m, 1 H, *p*-Ph), 6.20 (s, 5 H, Cp), 6.02 (s, 5 H, Cp), 5.82 (s, 10 H, Cp), 5.40 (s, 10 H, Cp), 4.66 (s, 1 H, CC(*H*)Ph), −1.45 ppm (s, 2 H, Zr*H*Zr). The signal of the *o*-Ph proton could not be located due to the presence of ethyl benzene. **^13^C{^1^H} NMR** (100.63 Hz,100 C_6_D_6_, 297 K): δ 230.6 (Zr*C*C(H)Ph), 157.0 (*i*-C), 123.1 (Ph), 121.5 (Ph), 120.2 (Ph), 109.1 (Cp), 108.9 (Cp), 108.2 (Cp), 106.2 (Cp), 64.4 ppm (ZrC*C*(H)Ph*)*. **MS** (CI^+^, isobutane): *m*/*z* (%) 103 (10) [C=CPhH]^+^, 107 (20) [CCPhH_2_]^+^, 321 (12) [Cp2ZrCCPh]^+^, 239 (78) [Cp_2_ZrMe]^+^, 456 (45) [(Cp_2_Zr)_2_Me]^+^, 542 (84) [Cp_2_Zr(CCPh)Cp_2_ZrMe]^+^. **CHN analysis** calc. for C_38_H_39_Zr_3_: C, 59.32; H, 5.11. Found: C, 50.01; H, 4.096. **IR** (ATR, neat, cm^-1^): 3086 (w), 2277 (w), 1834 (w), 1585 (w), 1478 (w), 1438 (w), 1396 (w), 1326 (w), 1212 (w), 1168 (w), 1124 (w), 1065 (w), 1013 (m), 981 (w), 786 (s), 747 (s), 716 (m), 701 (s), 670 (s), 628 (m), 601 (m), 585 (s), 540 (m), 496 (s).

**Figure S7.** ^1^H NMR spectrum of complex **7** recorded after two weeks storing at 60 °C in a J Young tube (400.13 MHz, C_6_D_6_, 297 K). Further identified reaction products: PhCH_2_CH_3_ (red arrows), (Cp_2_Zr(H)(µ-H_2_)(H)ZrCp_2_) (blue arrows), **6** (green arrows).

## Synthesis of (Cp_2_Zr)_2_(µ-CN-Py)(σ-C_2_Ph) (8)

(Cp_2_Zr)_2_(µ-Me)(µ-C_2_Ph) (**5**) (0.165 g, 0.296 mmol) and 2-cyanopyridine (0.54 mL, excess) were dissolved in 6 mL of benzene and the reaction mixture was allowed to stand at room temperature for 24 h without stirring. All volatiles were removed under vacuum and the remaining 2-cyanopyridine was distilled under vacuum at 60°C. The obtained intense orange to brown solid (**8**) was dissolved in toluene at 50°C, filtered, and concentrated yielding a brownish solid material. Brown needle shaped crystals of (**8**) were obtained from a 2:1 toluene:heptane solution at 10°C.

**Yield:** 0.30 g (0.043 mmol; 14%).

**^1^H NMR** (400.13 MHz, C_6_D_6_, 297 K): δ 8.40-8.39 (d, 1 H, Py), 7.89-7.86 (m, 2 H, *o*-Ph), 7.47-7.45 (m, 1 H, Py), 7.28-7.20 (m, 2 H, *m*-Ph), 7.24 – 7.20 (m, 1 H, Py), 7.11-7.07 (m, 1 H, *p*-Ph), 6.52-6.48 (m, 1 H, py), 5.82 (s, 10 H, Cp), 5.80 (s, 10 H, Cp), 0.10 (s, 3 H, Me). **^13^C{^1^H} NMR** (100.63 Hz, C_6_D_6_, 297 K): δ 233.5 (py*C*N), 157.7 (py), 151.4 (py), 140.2 (py), 139.2 (Zr*C*CPh), 131.3 (*o*-Ph), 129.5 (*i*-Ph), 128.6 (*m*-Ph), 127.1 (Py), 125.4 (*p*-Ph), 123.5 (py), 119.68 (ZrC*C*Ph), 111.1 (Cp), 105.5 (Cp), 30.2 (Zr*C*H_3_). **MS** (CI^+^, isobutane): *m*/*z* (%) 298 (100) [Cp_2_Zr-NCCCH_2_CH_2_CH_2_]^+^, 235 (6) [Cp_2_ZrMe]^+^. **Mp.**: 170°C (decomp.). **CHN analysis** calc. for C_36_H_36_N_2_Zr_2_: C, 63.67; H, 5.34; N, 4.12. Found: C, 57.93; H, 5.55; N, 3.12. **IR** (ATR, neat, cm^-1^): 3081 (w), 2930 (w), 2816 (w), 2077 (w), 1798 (w), 1596 (w), 1581 (m), 1559 (w), 1481 (w), 1466 (w), 1434 (m), 1363 (w), 1288 (w), 1216 (w), 1203 (w), 1171 (w), 1151 (w), 1133 (w), 1101 (w), 1055 (w), 1011 (m), 933 (w), 902 (w), 824 (m), 799 (s), 783 (s), 774 (s), 752 (s), 691 (s), 634 (m), 618 (w), 601 (w), 559 (w), 535 (s), 526 (s), 493 (m), 424 (m).

**Figure S8.** ^1^H NMR spectrum of complex **8** (400.13 MHz, C_6_D_6_, 297 K).

**Figure S9.** ^13^C{^1^H} NMR spectrum of complex **8** (100.63 MHz, C_6_D_6_, 297 K).

## Synthesis of (Cp_2_Zr)_2_(µ-NCCH_3_)(κ^2^-H_3_CC_2_Ph) (9)

(Cp_2_Zr)_2_(µ-Me)(µ-C_2_Ph) (**5**) (0.200 g, 0.358 mmol) was dissolved in 15 mL of benzene and acetonitrile (0.030 g, 0.7168 mmol) was added to the solution. The reaction mixture was stirred at 50°C overnight. The suspension was filtered, the remaining yellow solid (**9**) was washed with benzene and dried in vacuo for two hours.

**Yield**: 0.85 g (0.141mmol, 39%).

*Notes: Single crystals of the complex could be obtained from the benzene wash solution. Further analysis of this wash solution showed that it contained a mixture of several species. Further experiments with different stoichiometric ratios of* ***5*** *and nitrile did not optimise this synthesis.* *The yellow solid is poorly soluble in most non-polar solvents, decomposed in acetonitrile and DMSO, and showed moderate solubility in THF that led to slow decomposition.*

**^1^H NMR** (300.20 MHz, THF-*d*_8_, 297 K): δ 7.24 (m, 2 H, *o*-Ph), 6.96 (m, 2 H, *m*-Ph), 6.90 (m, 1 H, *p*-Ph), 5.72 (s, 10 H, Cp), 5.46 (s, 10 H, Cp), 2.79 (s, 3 H, Me), 2.01 (s, 3 H, Me). **^13^C{^1^H}** **NMR** (75.49 Hz, THF-*d*_8_, 297 K): δ 108.8 (Cp), 105.0 (Cp). **MS** (CI^+^, isobutane): *m*/*z* (%) 644 (100) [(Cp_2_Zr)_2_(NCCH_3_)(CCCH_3_Ph)+(CNCH_3)_]^+^, 236 (10) [Cp_2_ZrMe]^+^, 102 (15) [CCPh]^+^, 67 (12) [Cp]^+^. **CHN analysis** calc. for C_31_H_34_NZr_2_: C, 61.74; H, 5.68; N, 2.32. Found: C, 55.03; H, 3.158; N, 2.413. **IR:** (ATR, neat, cm^-1^): 2916 (w), 1835 (w), 1608 (w), 1563 (w), 1504 (w), 1479 (w), 1438 (w), 1355 (w), 1337 (w), 1263 (w), 1183 (w), 1131 (w), 1071 (w), 1011 (w), 981 (w), 846 (w), 793 (m), 748 (w), 730 (w), 705 (w), 686 (m), 661 (w), 638 (w), 603 (w), 574 (w), 543 (w), 491 (w), 453 (w), 437 (w).

**Figure S10.** ^1^H NMR spectrum of complex **9** (300.20 MHz, THF-*d*_8_, 297 K). Due to poor solubility in other solvents or decomposition of the compound, NMR data were recorded in THF, although the compound decomposes slowly in this solvent. It should be noted that even prolonged drying in vacuum does not reduce the amount of benzene labelled with #.

**Figure S11.** ^13^C{^1^H} NMR spectrum of complex **9** (75.49 MHz, THF-*d*_8_, 297 K). Due to the poor solubility only the Cp signals were detected. Impurities: # benzene.

# Computational details

Computational studies were carried out using *Gaussian16^10^* and the standalone version of *NBO 6.0*.^[[11]](#endnote-11)^ In this section, the main and secondary findings of a detailed DFT study are compiled to support the main findings discussed in the manuscript as well as to provide further detailed information. The real-size molecules were optimised using the hybrid density functional method B3LYP,^[[12]](#endnote-12),^^[[13]](#endnote-13)^ in combination with the basis set def2-TZVP,^[[14]](#endnote-14)^ and Grimmes empirical dispersion correction D3^[[15]](#endnote-15)^ (notation: B3LYP-D3/def2-TZVP). Vibrational frequencies were computed, to include zero-point vibrational energies in thermodynamic parameters and to characterise all structures as minima on the potential energy surface. Using these geometries a series of binding analyses like Natural Bonding Orbital (NBO), Natural Localised Molecular Orbital (NLMO), Quantum-Theory of Atoms in Molecules (QT-AIM) and Wiberg bond index calculations/visualisation was performed. The latter two were performed using MultiWfn 3.5^[[16]](#endnote-16)^, employing *Gaussian16* formatted checkpoint files. For the selected example of complex **7** an intrinsic bond orbital (IBO) and interaction region indicator (IRI) analysis was conducted.^[[17]](#endnote-17),^^[[18]](#endnote-18)^ In addition, a TD-DFT study was performed using the optimised geometries using PBE0^[[19]](#endnote-19)^-D3/def2-TZVP level of theory to investigate the charge transfers responsible for the observed colours of the complexes. The cube files of the Charge Density Difference (CDD)^[[20]](#endnote-20)^ of the excited stated were generated with MultiWfn 3.5.

For visualisation of 3D-quantum chemical results GaussView 6.1.1^[[21]](#endnote-21)^ or the Open-Source program Avogadro 1.2.0^[[22]](#endnote-22)^ were used. In addition to these Supporting Information, a multi-structure xyz file with all calculated molecules is provided. For a better understanding and a more intuitive view of the calculated 3D structures, use of this file, e.g., with the free program MERCURY is recommended.^[[23]](#endnote-23)^

*Please note that all computations were carried out for single, isolated molecules in the gas phase (ideal gas approximation). There may well be significant differences between gas phase and condensed phase.*

## Bond analysis of isolated complexes

### **Bond analysis of (Cp_2_Zr)_2_(µ-Me)(µ-C_2_Ph) (5)**


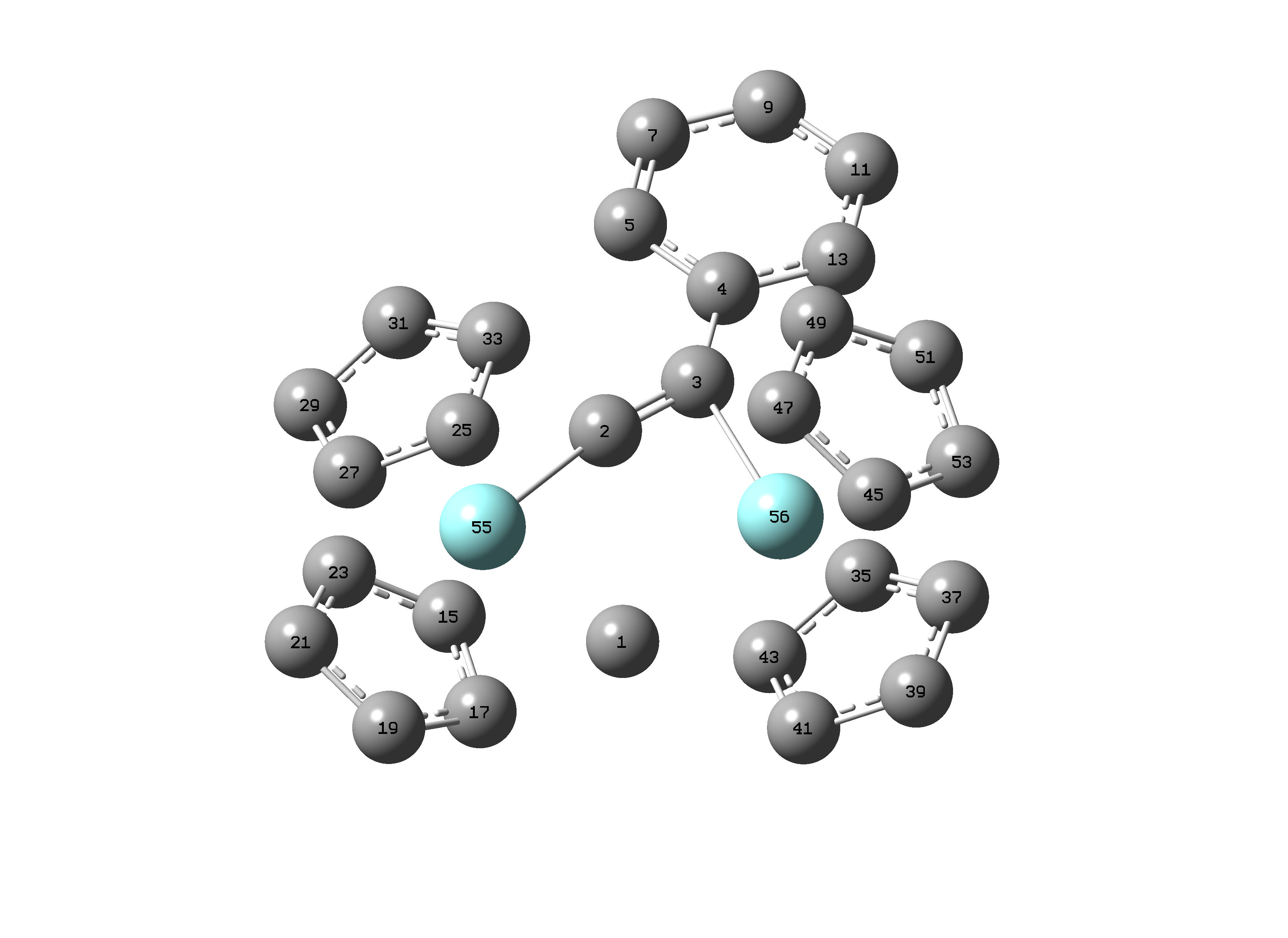


**Figure S12.** Labelling scheme for the bond analysis of complex **5**. Hydrogen atoms are omitted for clarity.

#### NBO/NLMO analysis of (Cp_2_Zr)_2_(µ-Me)(µ-C_2_Ph) (**5**)

For this purpose, the molecule was divided into four logical units and the natural charges of the atoms of these were summed up.

**Table S3.** Summary of selected NBO analysis of **5**.

| 44. (1.85782) BD ( 1) C 1-Zr 55  ( 82.75%) 0.9097* C 1 s( 37.11%)p 1.69( 62.87%)d 0.00( 0.01%) f 0.00( 0.01%)  ( 17.25%) 0.4153*Zr 55 s( 20.97%)p 0.00( 0.02%)d 3.77( 78.94%) f 0.00( 0.07%) | 48. (1.96193) BD ( 1) C 2- C 3  ( 47.31%) 0.6878* C 2 s( 39.52%)p 1.52( 60.14%)d 0.01( 0.29%) f 0.00( 0.04%)  ( 52.69%) 0.7259* C 3 s( 36.89%)p 1.71( 62.93%)d 0.00( 0.10%) f 0.00( 0.08%) |
| --- | --- |
| 49. (1.78203) BD ( 2) C 2- C 3  ( 46.30%) 0.6804* C 2 s( 0.24%)p99.99( 99.52%)d 0.87( 0.21%) f 0.14( 0.03%)  ( 53.70%) 0.7328* C 3 s( 7.70%)p11.97( 92.16%)d 0.01( 0.08%) f 0.01( 0.06%) | 50. (1.77113) BD ( 3) C 2- C 3  ( 43.82%) 0.6619* C 2 s( 0.21%)p99.99( 99.57%)d 0.96( 0.20%) f 0.15( 0.03%)  56.18%) 0.7496* C 3 s( 12.81%)p 6.80( 87.06%)d 0.01( 0.08%) f 0.00( 0.05%) |
| 51. (1.86848) BD ( 1) C 2-Zr 55  ( 80.25%) 0.8958* C 2 s( 59.78%)p 0.67( 40.15%)d 0.00( 0.05%) f 0.00( 0.02%)  ( 19.75%) 0.4444*Zr 55 s( 13.39%)p 0.01( 0.16%)d 6.45( 86.40%) f 0.00( 0.05%) | 38. (0.99511) LP ( 1) C 4  s( 0.00%)p 1.00( 99.95%)d 0.00( 0.01%) f 0.00( 0.04%) |

**Table S4.** Summary of selected NLMOs of **5** (iso0.04).

| 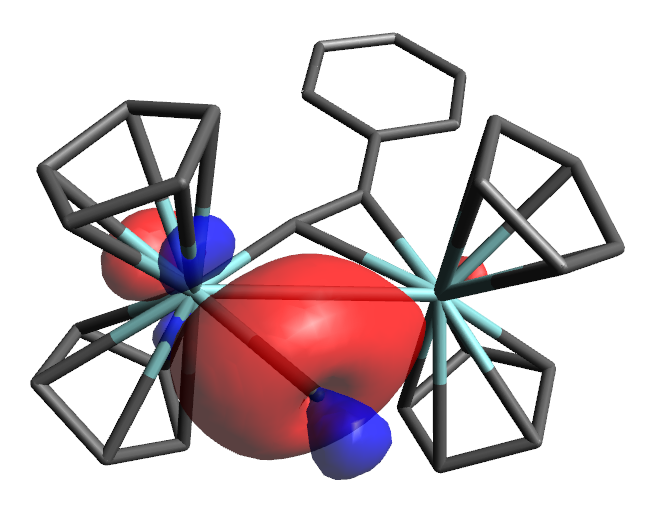 | 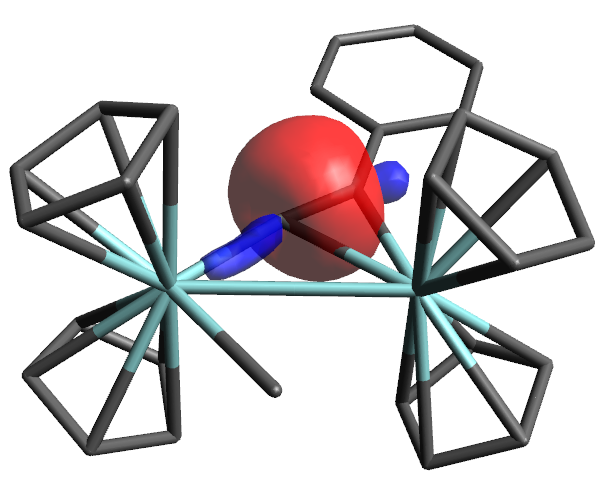 |
| --- | --- |
| Shortened NLMO 44 Analysis of C(1)-Zr(55) σ-bond as well as smaller C(1)-Zr(56) binding contribution (threshold > 1.3%)  44. (2.00000) 92.2110% BD ( 1) C 1-Zr 55  76.924% C 1 s( 33.03%)p 2.03( 66.95%)d 0.00( 0.01%) f 0.00( 0.01%)  15.576% Zr 55 s( 19.71%)p 0.00( 0.02%)d 4.07( 80.21%) f 0.00( 0.06%)  6.325% Zr 56 s( 13.70%)p 0.02( 0.26%)d 6.27( 85.93%)  f 0.01( 0.10%) | Shortened NLMO 48 Analysis of C(2)-C(3) σ-bond (threshold > 1.3%)  48. (2.00000) 98.0783% BD ( 1) C 2- C 3  46.417% C 2 s( 37.06%)p 1.69( 62.60%)d 0.01( 0.29%) f 0.00( 0.04%)  51.817% C 3 s( 32.37%)p 2.08( 67.45%)d 0.00( 0.10%) f 0.00( 0.08%) |
| 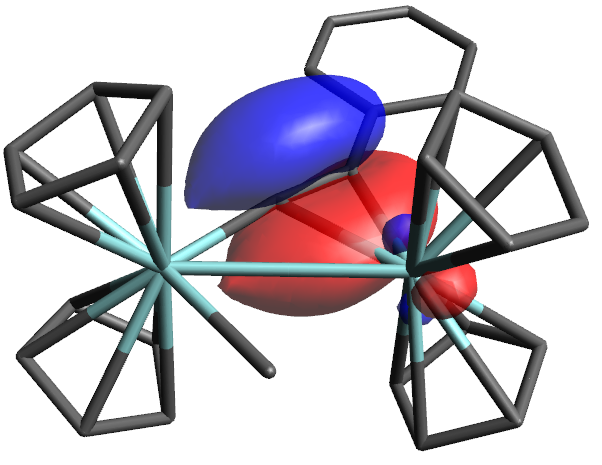 | 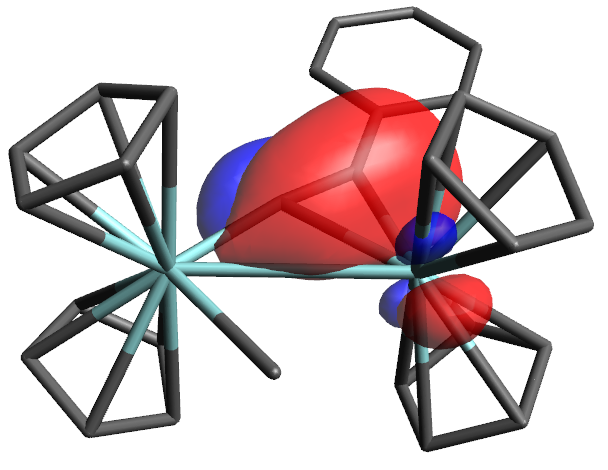 |
| Shortened NLMO 49 Analysis of C(2)-C(3) π-bond indicates also small contributions by both Zr atoms (threshold > 1.3%)  49. (2.00000) 87.9625% BD ( 2) C 2- C 3  43.352% C 2 s( 0.14%)p99.99( 99.61%)d 1.53( 0.21%) f 0.27( 0.04%)  45.050% C 3 s( 5.79%)p16.24( 94.06%)d 0.01( 0.09%) f 0.01( 0.06%)  2.939% Zr 55 s( 0.49%)p 1.08( 0.53%)d99.99( 98.70%) f 0.60( 0.29%)  5.069% Zr 56 s( 8.91%)p 0.03( 0.23%)d10.16( 90.52%) f 0.04( 0.33%) | Shortened NLMO 50 Analysis of C(2)-C(3) π-bond indicates also small contributions by both Zr atoms (threshold > 1.3%)  50. (2.00000) 87.4365% BD ( 3) C 2- C 3  38.532% C 2 s( 0.42%)p99.99( 99.35%)d 0.48( 0.20%) f 0.08( 0.03%)  49.304% C 3 s( 13.35%)p 6.48( 86.54%)d 0.01( 0.07%) f 0.00( 0.04%)  3.079% Zr 55 s( 1.46%)p 0.32( 0.47%)d67.03( 97.89%) f 0.13( 0.19%)  6.128% Zr 56 s( 19.60%)p 0.01( 0.16%)d 4.08( 80.05%) f 0.01( 0.19%) |
| 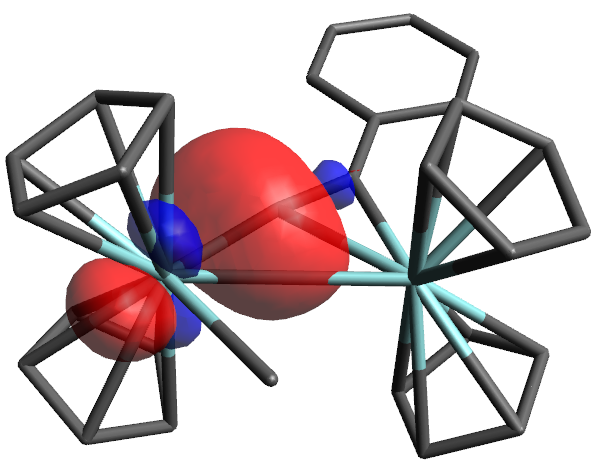 | 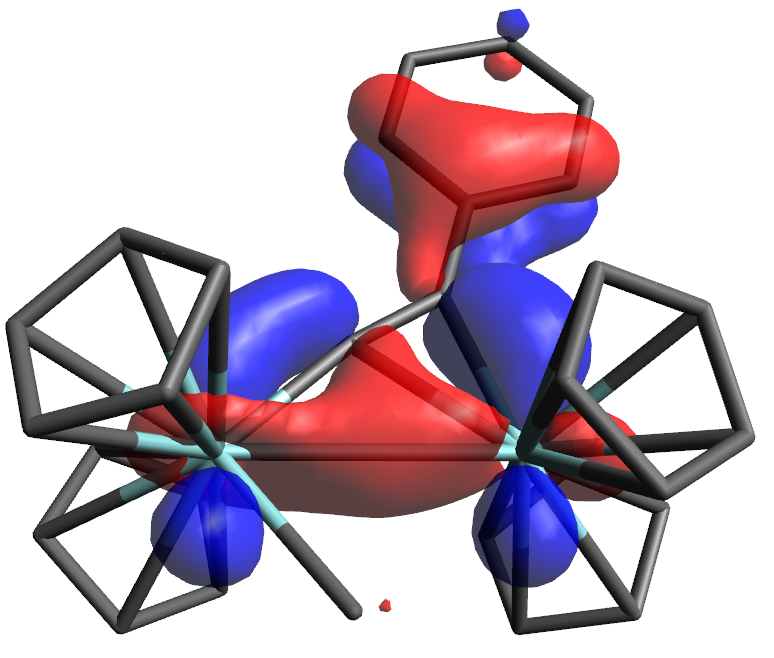 |
| Shortened NLMO 51 Analysis of C(1)-Zr(55) σ-bond as well as smaller C(1)-Zr(56) binding contribution (threshold > 1.3%)  51. (2.00000) 92.2619% BD ( 1) C 2-Zr 55  76.456% C 2 s( 54.66%)p 0.83( 45.28%)d 0.00( 0.04%) f 0.00( 0.02%)  17.688% Zr 55 s( 16.47%)p 0.01( 0.16%)d 5.06( 83.32%) f 0.00( 0.05%)  4.172% Zr 56 s( 28.12%)p 0.00( 0.09%)d 2.55( 71.67%) f 0.00( 0.12%) | Shortened NLMO 38 Analysis of formal LP C(4) reveal a strong delocalization with only 14.6% of the origin LP, it shows binding contributions between both Zr centres and anti-binding features of the alkynyl unit (threshold > 1.3%)  38. (2.00000) 14.5844% LP ( 1) C 4  1.610% C 1 s( 8.27%)p11.05( 91.34%)d 0.04( 0.35%) f 0.01( 0.04%)  12.904% C 2 s( 0.11%)p99.99( 99.17%)d 5.83( 0.65%) f 0.65( 0.07%)  14.887% C 3 s( 22.05%)p 3.53( 77.77%)d 0.01( 0.16%) f 0.00( 0.02%)  15.057% C 4 s( 1.49%)p66.08( 98.41%)d 0.04( 0.05%) f 0.03( 0.05%)  6.393% C 5 s( 0.45%)p99.99( 99.42%)d 0.19( 0.08%) f 0.10( 0.05%)  1.684% C 9 s( 0.25%)p99.99( 99.56%)d 0.49( 0.12%) f 0.29( 0.07%)  6.219% C 13 s( 0.41%)p99.99( 99.47%)d 0.20( 0.08%)f 0.11( 0.04%)  14.139% Zr 55 s( 0.09%)p 1.42( 0.13%)d99.99( 99.74%)f 0.42( 0.04%)  21.185% Zr 56 s( 0.43%)p 0.30( 0.13%)d99.99( 99.43%)f 0.03( 0.01%) |

#### QT-AIM analysis of (Cp_2_Zr)_2_(µ-Me)(µ-C_2_Ph) (**5**)


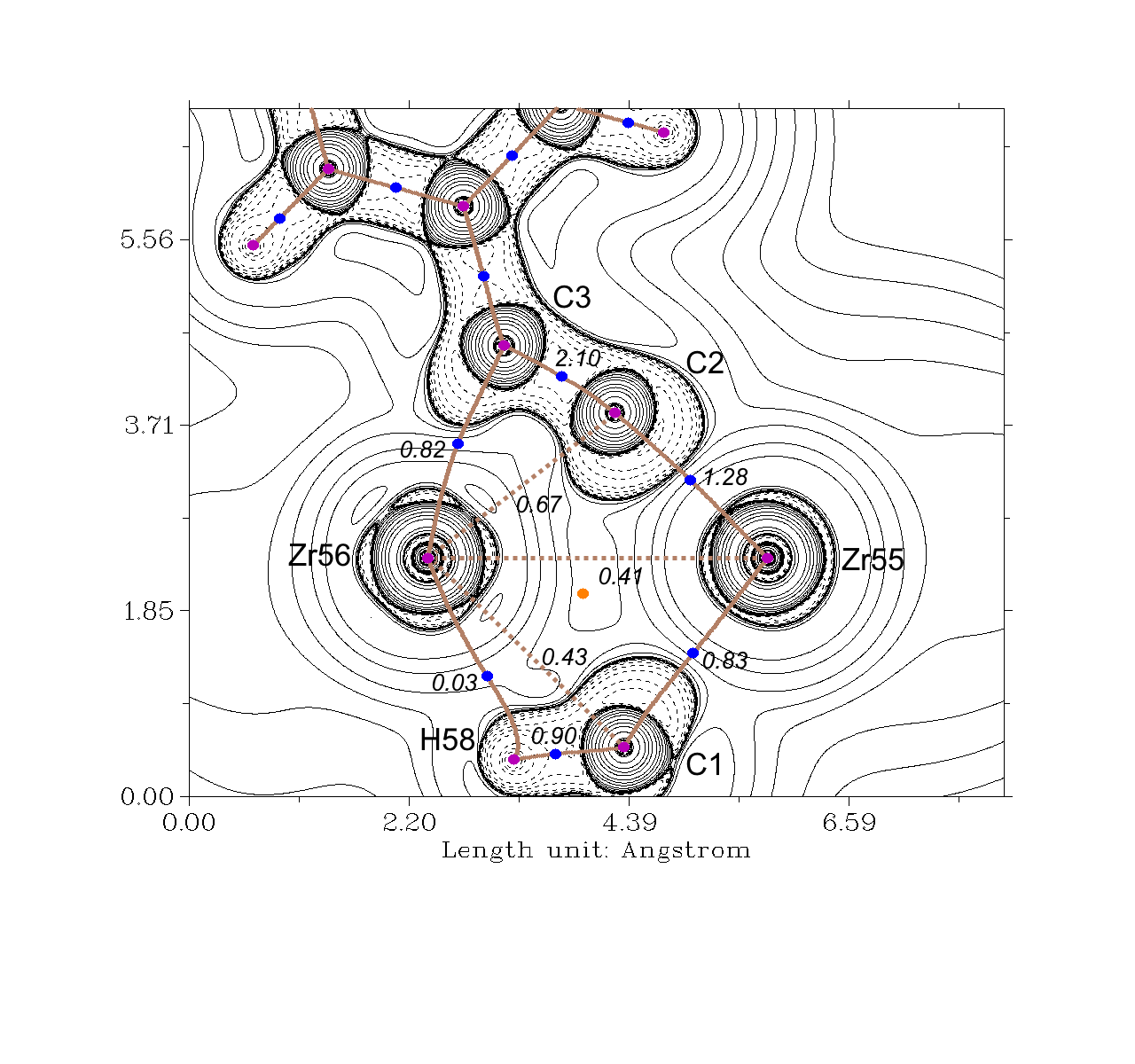


**Figure S13.** Contour plot of the Laplacian of the electron density ∇^2^*r* of complex **5** in the Zr56-Zr55-C3 plane. Dashed lines indicate negative (local charge concentration), solid lines indicate positive values (local charge depletion). The Laplacian plot is overlaid with the molecular graph from QT-AIM analysis and Wiberg bond indices (italic small numbers). Brown lines indicate bond paths, brown dashed lines are hypothetical bonds, blue dots correspond to bond critical points, light brown dots indicate ring critical points. Density from B3LYP-D3/def2-TZVP calculation.

### **Bond analysis of (Cp_2_Zr)_2_(µ-H)(µ-C_2_Ph) (6)**


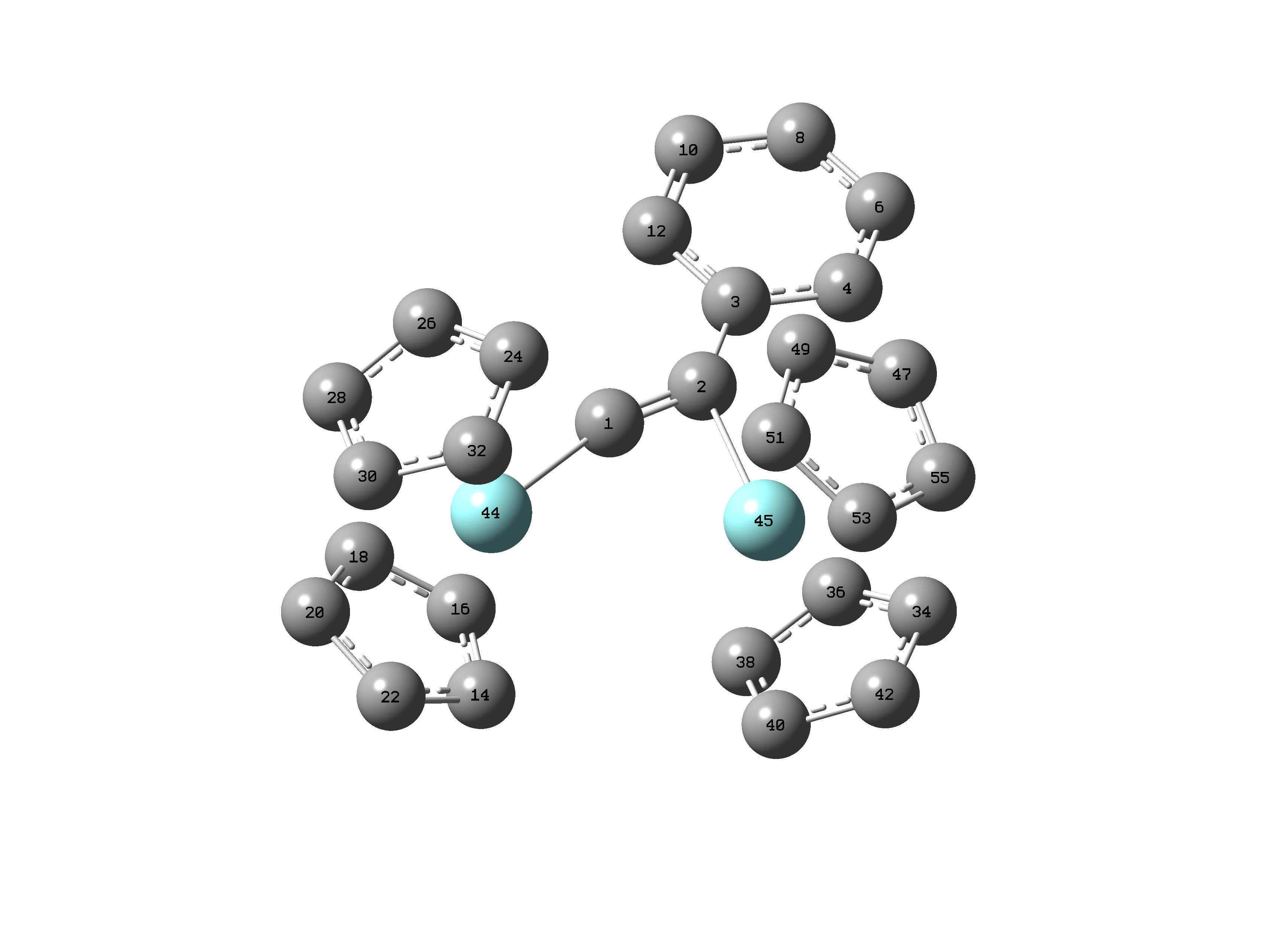


**Figure S14.** Labelling scheme for the bond analysis of complex **6**. Hydrogen atoms are omitted for clarity. H46 is the bridging hydride.

#### NBO/NLMO analysis of (Cp_2_Zr)_2_(µ-H)(µ-C_2_Ph) (**6**)

For this purpose, the molecule was divided into four logical units and the natural charges of the atoms of these were summed up.

**Table S5.** Summary of selected NBO analysis of **6**.

| 41. (1.96257) BD ( 1) C 1- C 2  ( 47.20%) 0.6870* C 1 s( 44.18%)p 1.26( 55.52%)d 0.01( 0.27%) f 0.00( 0.04%)  ( 52.80%) 0.7267* C 2 s( 36.23%)p 1.75( 63.59%)d 0.00( 0.10%) f 0.00( 0.08%) | 42. (1.77739) BD ( 2) C 1- C 2  ( 48.43%) 0.6959* C 1 s( 0.37%) p99.99 (99.40%)d 0.52( 0.19%) f 0.09( 0.03%)  ( 51.57%) 0.7181* C 2 s( 3.79%)p25.38( 96.06%)d 0.02( 0.09%) f 0.02( 0.07%) |
| --- | --- |
| 43. (1.76477) BD ( 3) C 1- C 2  ( 42.98%) 0.6556* C 1 s( 2.28%)p42.87( 97.53%)d 0.08( 0.18%) f 0.01( 0.02%)  ( 57.02%) 0.7551* C 2 s( 17.65%)p 4.66( 82.24%)d 0.00( 0.08%) f 0.00( 0.04%) | 44. (1.63518) BD ( 1) C 1-Zr 45  ( 86.23%) 0.9286* C 1 s( 52.79%)p 0.89( 47.11%)d 0.00( 0.08%) f 0.00( 0.02%)  ( 13.77%) 0.3710*Zr 45 s( 12.36%)p 0.00( 0.04%)d 7.08( 87.52%) f 0.01( 0.07%) |
| 96. (1.65749) BD ( 1)Zr 44- H 46  ( 23.71%) 0.4869*Zr 44 s( 15.68%)p 0.01( 0.13%)d 5.37( 84.11%) f 0.01( 0.08%)  ( 76.29%) 0.8734* H 46 s( 99.91%)p 0.00( 0.09%) | 109. (0.68490) BD*( 3) C 1- C 2  ( 57.02%) 0.7551* C 1 s( 2.28%)p42.87( 97.53%)d 0.08( 0.18%) f 0.01( 0.02%)  ( 42.98%) -0.6556* C 2 s( 17.65%)p 4.66( 82.24%)d 0.00( 0.08%) f 0.00( 0.04%) |

**Table S6.** Summary of selected NLMOs of **6** (iso0.04).

| 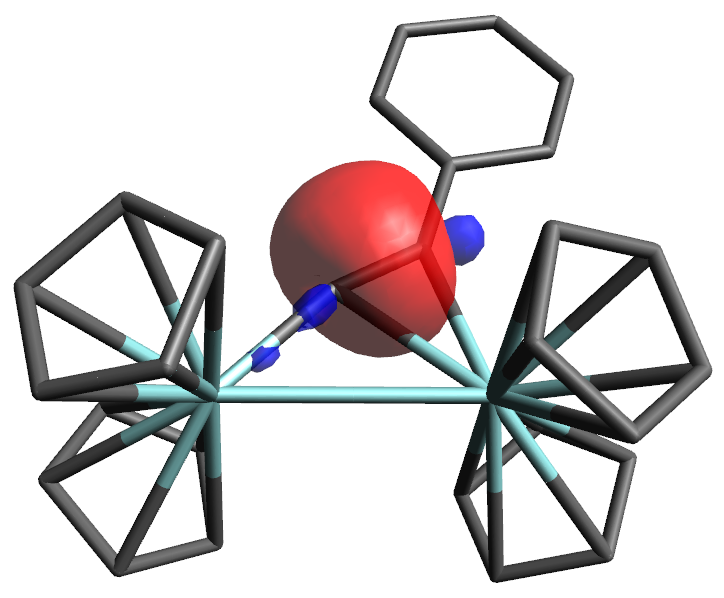 | 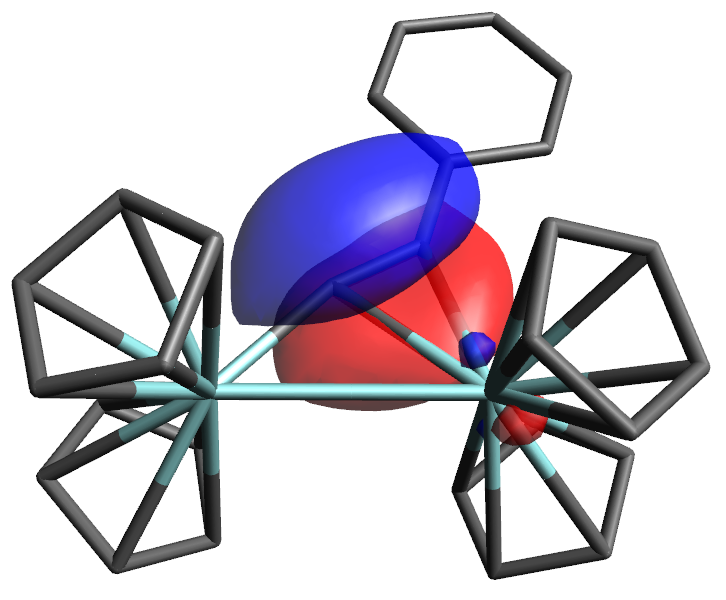 |
| --- | --- |
| Shortened NLMO 41 Analysis of C(1)-C(2) σ-bond (threshold > 1.3%)  41. (2.00000) 98.1098% BD ( 1) C 1- C 2  46.381% C 1 s( 40.28%)p 1.48( 59.42%)d 0.01( 0.27%) f 0.00( 0.04%)  51.901% C 2 s( 32.42%)p 2.08( 67.40%)d 0.00( 0.10%) f 0.00( 0.08%) | Shortened NLMO 42 Analysis of C(1)-C(2) π-bond indicates also small contributions by both Zr atoms (threshold > 1.3%)  42. (2.00000) 88.3585% BD ( 2) C 1- C 2  43.341% C 1 s( 0.36%)p99.99( 99.41%)d 0.55( 0.20%) f 0.10( 0.04%)  45.272% C 2 s( 2.92%)p33.19( 96.92%)d 0.03( 0.09%) f 0.02( 0.07%)  2.757% Zr 44 s( 0.22%)p 2.49( 0.54%)d99.99( 98.88%) f 1.60( 0.35%)  4.802% Zr 45 s( 2.57%)p 0.12( 0.32%)d37.66( 96.69%) f 0.17( 0.43%) |
| 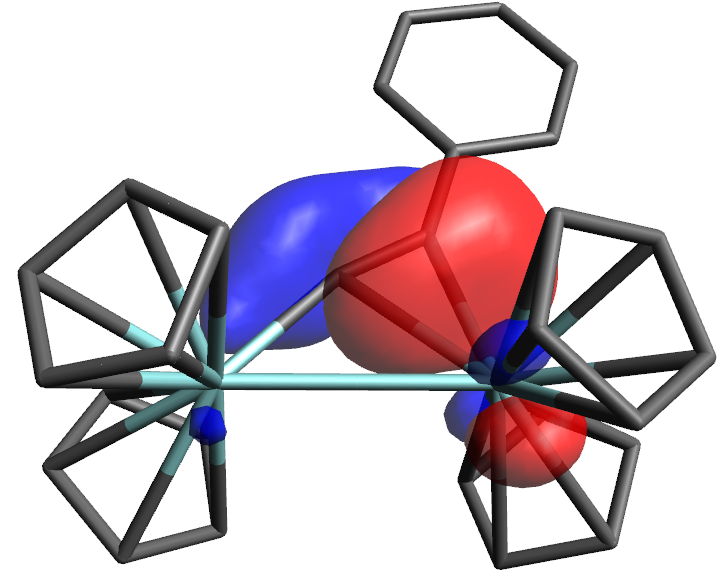 | 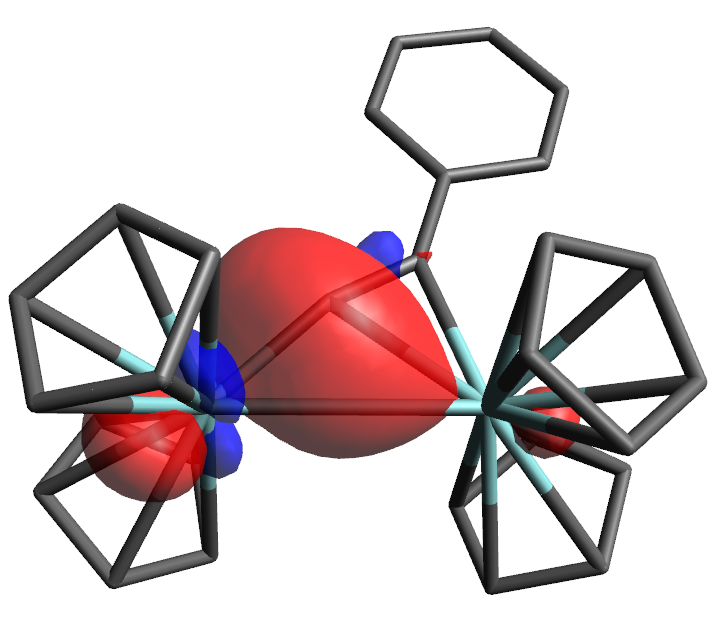 |
| Shortened NLMO 43 Analysis of C(1)-C(2) π-bond indicates also small contributions by both Zr atoms (threshold > 1.3%)  43. (2.00000) 87.5696% BD ( 3) C 1- C 2  38.151% C 1 s( 2.55%)p38.19( 97.26%)d 0.07( 0.17%) f 0.01( 0.02%)  49.719% C 2 s( 16.92%)p 4.90( 82.97%)d 0.00( 0.07%) f 0.00( 0.04%)  3.471% Zr 44 s( 1.10%)p 0.36( 0.39%)d89.71( 98.35%) f 0.15( 0.16%)  6.895% Zr 45 s( 11.51%)p 0.01( 0.12%)d 7.67( 88.24%) f 0.01( 0.13%) | Shortened NLMO 44 Analysis of C(1)-Zr(45) σ-bond indicates also larger contributions by the Zr44 atom (threshold > 1.3%)  44. (2.00000) 79.8335% BD ( 1) C 1-Zr 45  69.731% C 1 s( 50.48%)p 0.98( 49.42%)d 0.00( 0.08%) f 0.00( 0.02%)  17.694% Zr 44 s( 11.27%)p 0.01( 0.09%)d 7.86( 88.58%) f 0.00( 0.05%)  10.246% Zr 45 s( 11.08%)p 0.00( 0.03%)d 8.02( 88.83%) f 0.01( 0.07%) |
| 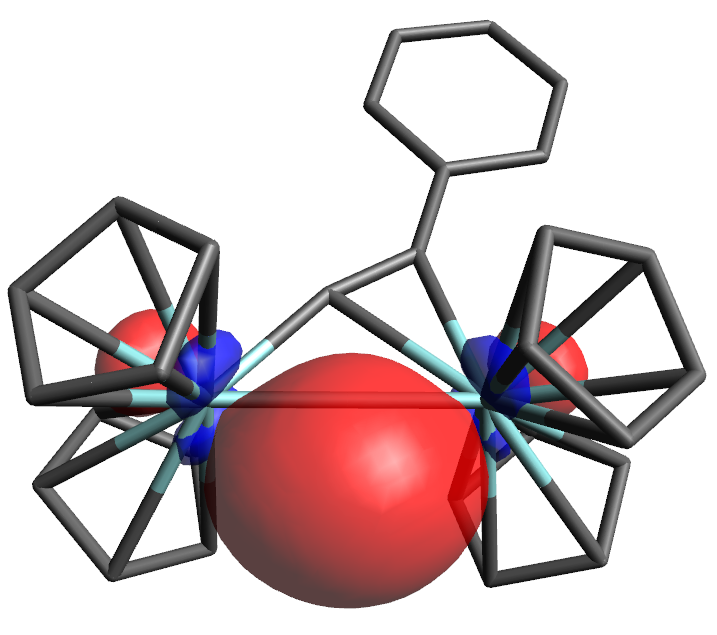 | 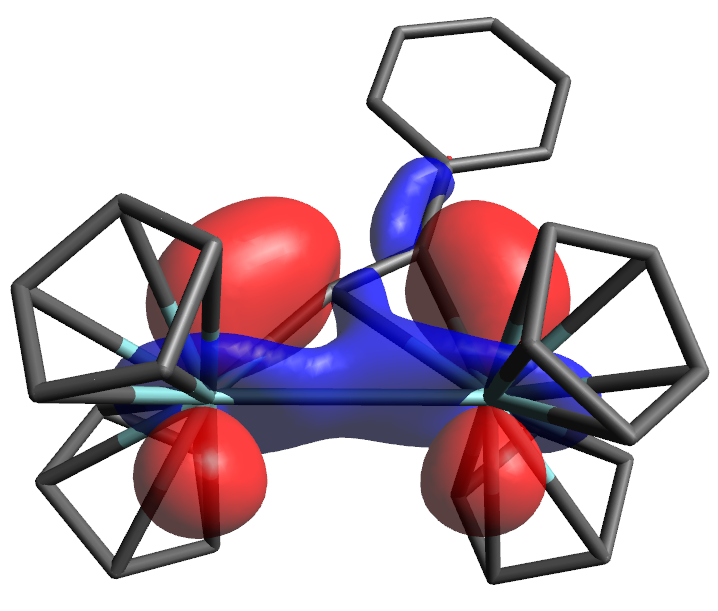 |
| Shortened NLMO 96 Analysis of H(46)-Zr(44) σ-bond reveal almost equal contributions of both Zr atoms (threshold > 1.3%)  96. (2.00000) 81.5783% BD ( 1)Zr 44- H 46  18.741% Zr 44 s( 15.68%)p 0.01( 0.14%)d 5.36( 84.10%) f 0.01( 0.09%)  16.977% Zr 45 s( 14.93%)p 0.01( 0.20%)d 5.68( 84.78%) f 0.01( 0.09%)  62.856% H 46 s( 99.91%)p 0.00( 0.09%) | Shortened NLMO 109 Analysis of C(1)-C(2) π*-bond reveal strong almost equal contributions of both Zr atoms (threshold > 1.3%)  109. (2.00000) 31.9692% BD*( 3) C 1- C 2  23.239% C 1 s( 15.28%)p 5.52( 84.33%)d 0.02( 0.35%) f 0.00( 0.05%)  17.921% C 2 s( 25.02%)p 2.99( 74.87%)d 0.00( 0.09%) f 0.00( 0.02%)  25.549% Zr 44 s( 3.53%)p 0.02( 0.09%)d27.25( 96.34%) f 0.01( 0.04%)  26.374% Zr 45 s( 3.33%)p 0.04( 0.12%)d28.99( 96.53%) f 0.01( 0.02%) |
|  |  |

#### QT-AIM analysis of (Cp_2_Zr)_2_(µ-H)(µ-C_2_Ph) (**6**)


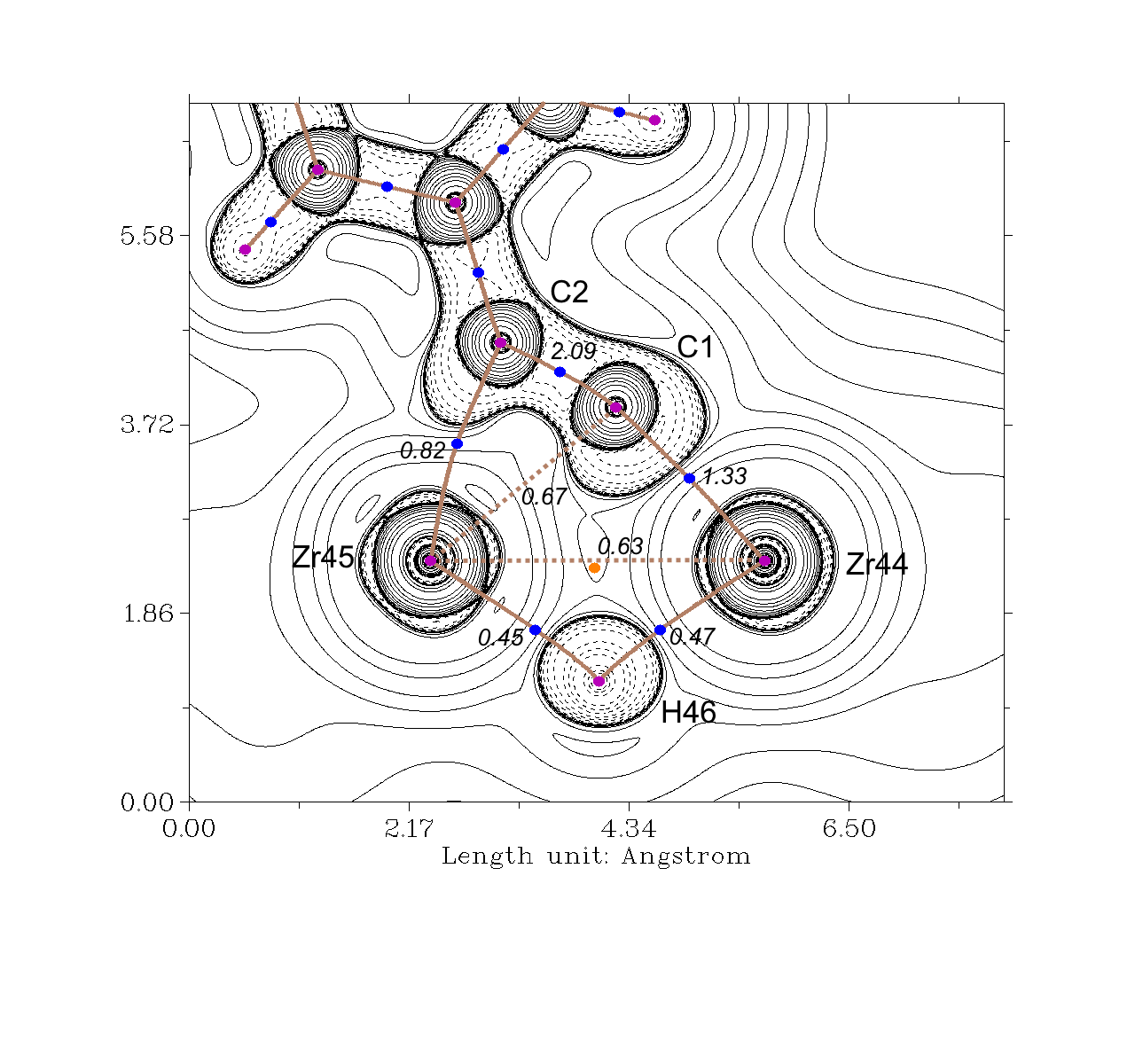


**Figure S15.** Contour plot of the Laplacian of the electron density ∇^2^*r* of complex **6** in the Zr45-Zr44-C2 plane. Dashed lines indicate negative (local charge concentration), solid lines indicate positive values (local charge depletion). The Laplacian plot is overlaid with the molecular graph from QT-AIM analysis and Wiberg bond indices (italic small numbers). Brown lines indicate bond paths, brown dashed lines are hypothetical bonds, blue dots correspond to bond critical points, light brown dots indicate ring critical points. Density from B3LYP-D3/def2-TZVP calculation.

### **Bond analysis of (Cp_2_Zr)_3_(µ-H)_2_(σ-µ C=CHPh) (7)**


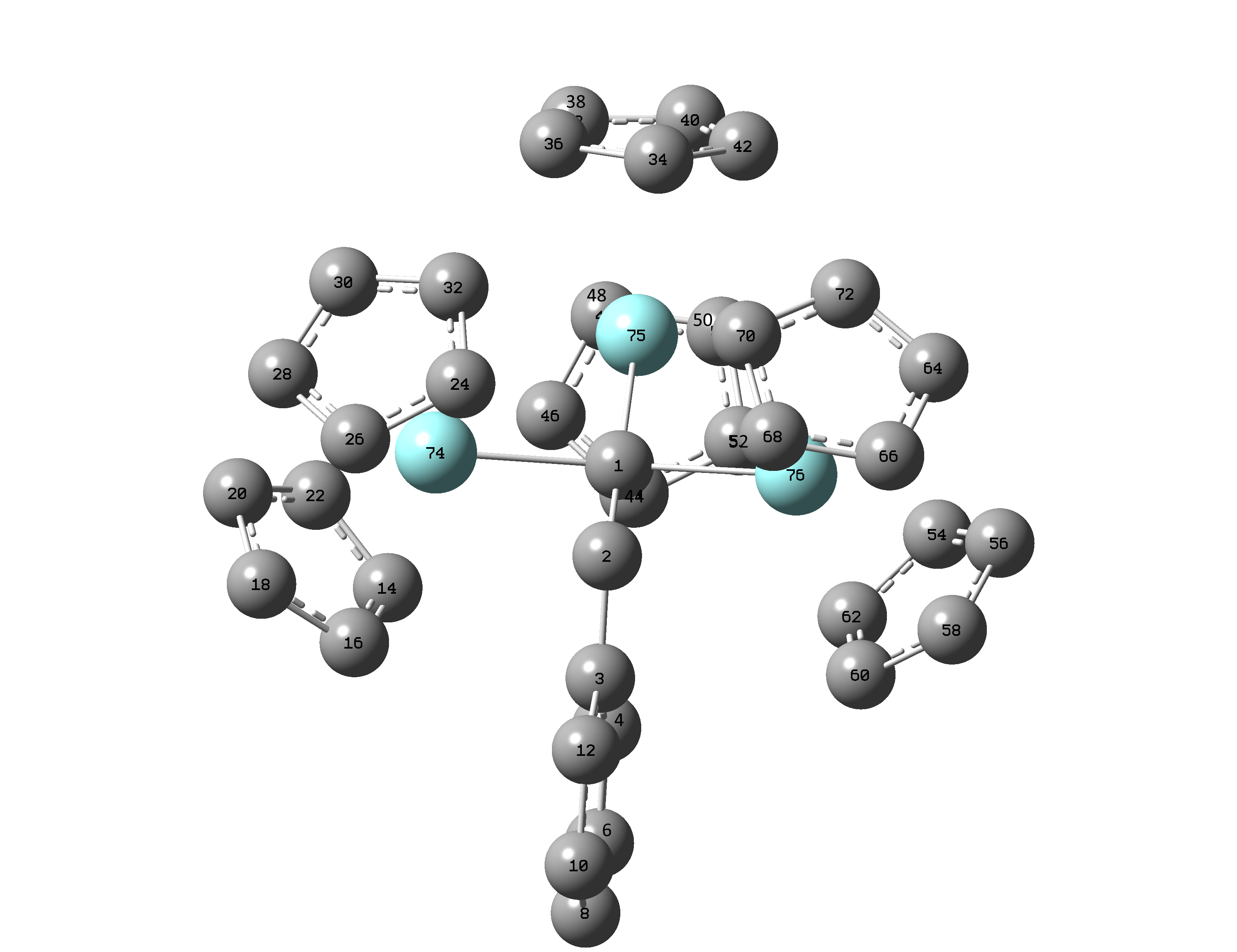
 **Figure S16.** Labelling scheme for the bond analysis of complex **7**. Hydrogen atoms are omitted for clarity. H77 is located on C2, H78 and H79 are the bridging hydrides.

#### NBO/NLMO and IBO analysis of (Cp_2_Zr)_3_(µ-H_2_)(σ-µ-C=CHPh) (**7**)

For this purpose, the molecule was divided into five logical units and the natural charges of the atoms of these were summed up.

**Table S7.** Summary of selected NBO analysis of **7**.

| 58. (1.89215) BD ( 1) C 1- C 2  ( 46.88%) 0.6847* C 1 s( 31.12%)p 2.21( 68.69%)d 0.00( 0.15%) f 0.00( 0.03%)  ( 53.12%) 0.7288* C 2 s( 39.25%)p 1.54( 60.58%)d 0.00( 0.12%) f 0.00( 0.05%) | 59. (1.75855) BD ( 1) C 1-Zr 75  ( 79.72%) 0.8929* C 1 s( 61.52%)p 0.62( 38.38%)d 0.00( 0.09%) f 0.00( 0.01%)  ( 20.28%) 0.4503*Zr 75 s( 8.18%)p 0.02( 0.17%)d11.19( 91.59%) f 0.01( 0.05%) |
| --- | --- |
| 60. (1.57140) BD ( 2) C 1-Zr 75  ( 81.98%) 0.9054* C 1 s( 5.38%)p17.59( 94.58%)d 0.00( 0.01%) f 0.01( 0.03%)  ( 18.02%) 0.4245*Zr 75 s( 1.25%)p 0.07( 0.09%)d78.55( 98.55%) f 0.08( 0.10%) | 61. (1.56523) BD ( 3) C 1-Zr 75  ( 84.61%) 0.9198* C 1 s( 1.69%)p58.06( 98.26%)d 0.01( 0.02%) f 0.02( 0.03%)  ( 15.39%) 0.3923*Zr 75 s( 0.40%)p 0.15( 0.06%)d99.99( 99.44%) f 0.26( 0.11%) |
| 63. (1.59697) BD ( 1) C 2-Zr 76  ( 85.76%) 0.9261* C 2 s( 0.57%)p99.99( 99.33%)d 0.14( 0.08%) f 0.03( 0.02%)  ( 14.24%) 0.3773*Zr 76 s( 3.52%)p 0.06( 0.23%)d27.30( 96.20%) f 0.01( 0.05%) | 64. (1.90276) BD ( 1) C 2- H 77  ( 61.73%) 0.7857* C 2 s( 26.34%)p 2.79( 73.60%)d 0.00( 0.05%) f 0.00( 0.01%)  ( 38.27%) 0.6186* H 77 s( 99.89%)p 0.00( 0.11%) |
| 150. (1.67500) BD ( 1)Zr 74- H 79  ( 24.81%) 0.4981*Zr 74 s( 12.00%)p 0.02( 0.29%)d 7.30( 87.62%) f 0.01( 0.08%)  ( 75.19%) 0.8671* H 79 s( 99.90%)p 0.00( 0.10%) | 151. (1.67263) BD ( 1)Zr 76- H 78  ( 24.71%) 0.4971*Zr 76 s( 10.70%)p 0.03( 0.29%)d 8.31( 88.93%) f 0.01( 0.08%)  ( 75.29%) 0.8677* H 78 s( 99.90%)p 0.00( 0.10%) |

***Table S8*.** Summary of selected NLMOs of **6** (iso0.04) and direct comparison with related IBOs.

| 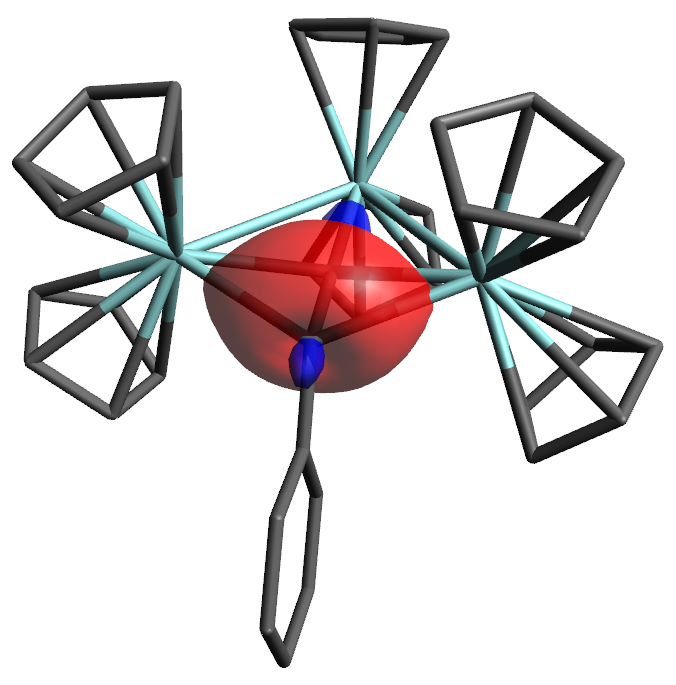 | 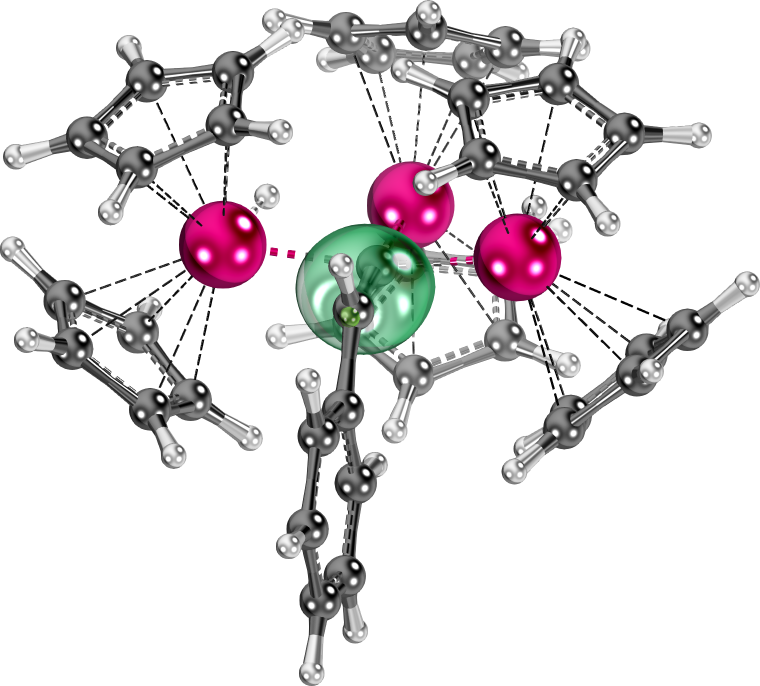 |
| --- | --- |
| Shortened NLMO 58 Analysis of C(1)-C(2) σ-bond indicates also small contributions by the bridged Zr(74,76) atoms (threshold > 1.3%)  58. (2.00000) 94.3830% BD ( 1) C 1- C 2  44.384% C 1 s( 27.26%)p 2.66( 72.56%)d 0.01( 0.14%) f 0.00( 0.03%)  50.167% C 2 s( 35.83%)p 1.79( 64.00%)d 0.00( 0.13%) f 0.00( 0.04%)  2.347% Zr 74 s( 8.48%)p 0.10( 0.83%)d10.66( 90.43%) f 0.03( 0.26%)  2.006% Zr 76 s( 7.63%)p 0.12( 0.90%)d11.94( 91.19%)f 0.04( 0.28%) | IBO of C(1)-C(2) σ-bond indicates also small contributions by the bridged Zr(74,76) atoms  Sel. Desc.  0.993 52.1 [E = -0.6812 O =AB]  Centres/Charges  C 1 0.946  C 2 0.993  ZR 74 0.027  ZR 76 0.028 |
| 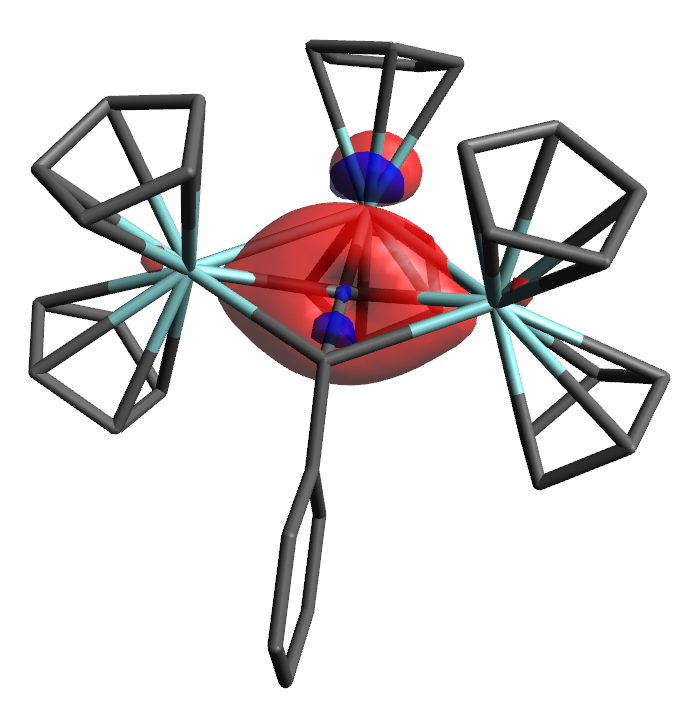 | 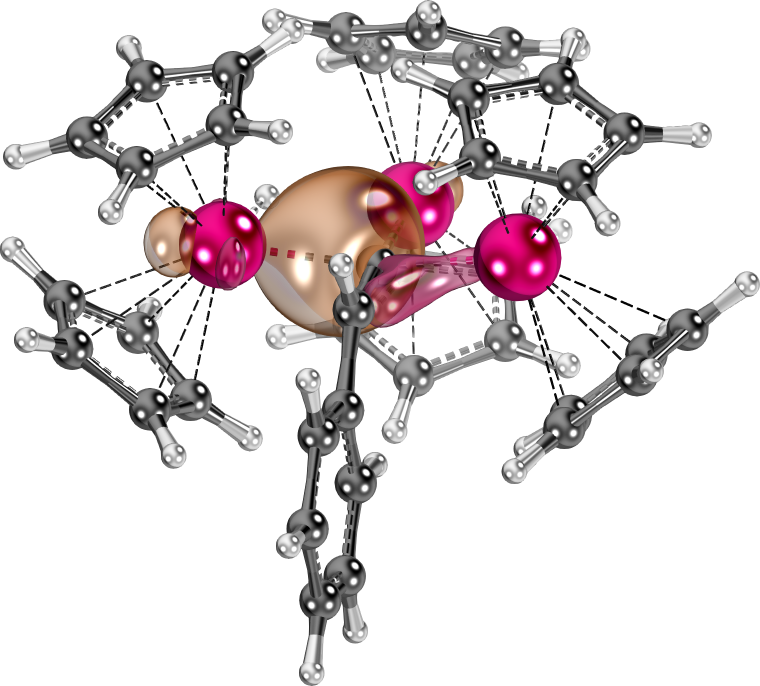 |
| Shortened NLMO 59 Analysis of C(1)-Zr(75) σ-bond indicates a strong polarization to the carbon atom as well as contributions by all three Zr atoms (threshold > 1.3%)  59. (2.00000) 87.1251% BD ( 1) C 1-Zr 75  70.428% C 1 s( 57.95%)p 0.72( 41.95%)d 0.00( 0.08%) f 0.00( 0.01%)  5.757% Zr 74 s( 14.94%)p 0.02( 0.26%)d 5.66( 84.64%) f 0.01( 0.15%)  16.964% Zr 75 s( 8.91%)p 0.02( 0.17%)d10.19( 90.85%) f 0.01( 0.06%)  5.048% Zr 76 s( 16.04%)p 0.02( 0.30%)d 5.21( 83.49%) f 0.01( 0.16%) | sigma bond ZrC1Zr first  Sel. Desc.  1.764* 125.1 [E = -0.4577 O =AB]  Centres/Charges  C 1 1.764  ZR 75 0.090  ZR 74 0.090 (other: 0.056) |
| 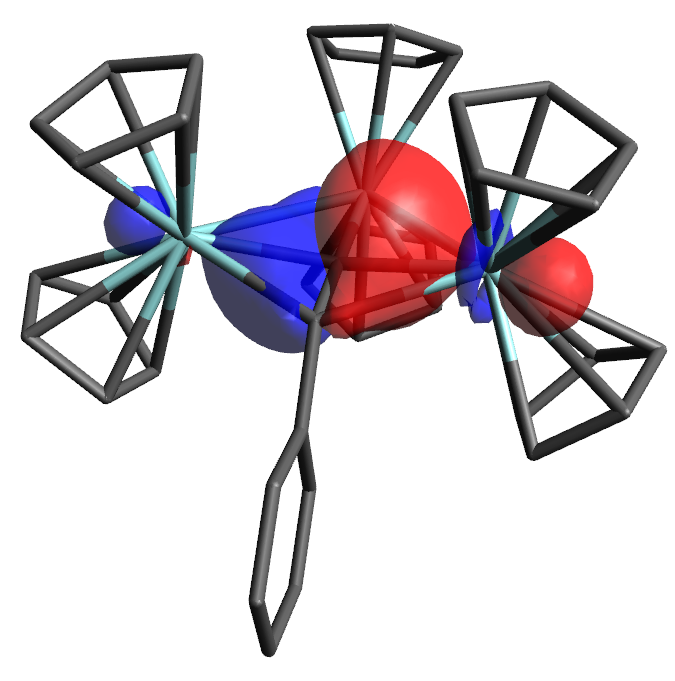 | 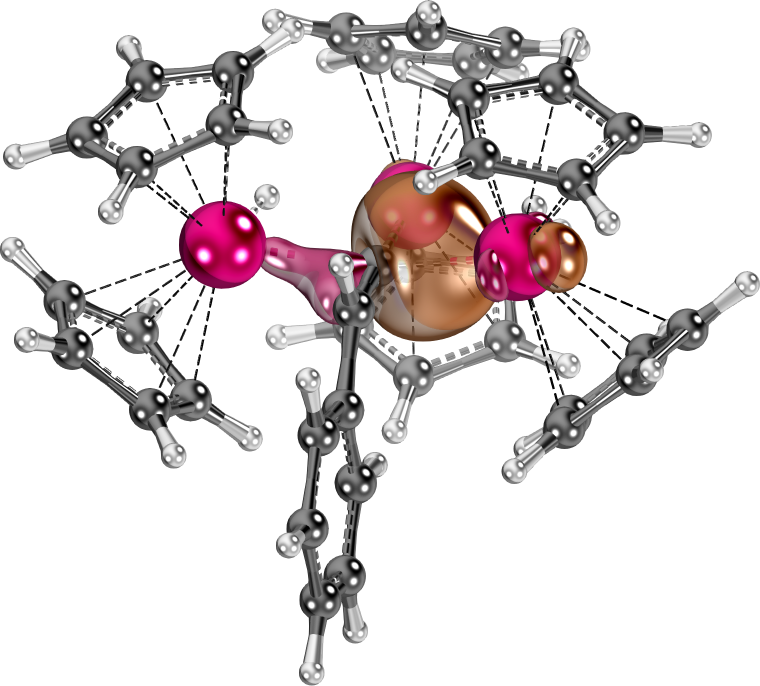 |
| Shortened NLMO 61 Analysis of C(1)-Zr(75) ) π-bond indicates also contributions by the bridged Zr(74,76) atoms and small contributions by C2 (threshold > 1.3%)  61. (2.00000) 76.4115% BD ( 3) C 1-Zr 75  66.555% C 1 s( 2.72%)p35.76( 97.24%)d 0.01( 0.02%) f 0.01( 0.03%)  2.316% C 2 s( 0.10%)p99.99( 99.12%)d 7.69( 0.75%) f 0.36( 0.03%)  5.366% Zr 74 s( 0.52%)p 0.38( 0.20%)d99.99( 99.11%) f 0.33( 0.17%)  10.170% Zr 75 s( 0.69%)p 0.10( 0.07%)d99.99( 99.10%) f 0.20( 0.14%)  12.618% Zr 76 s( 1.38%)p 0.08( 0.12%)d71.41( 98.44%) f 0.05( 0.06%) | sigma bond ZrC1Zr second  Sel. Desc.  1.763* 126.1 [E = -0.4540 O =AB]  Centres/Charges  C 1 1.763  ZR 76 0.090  ZR 75 0.089 (other: 0.057) |
| 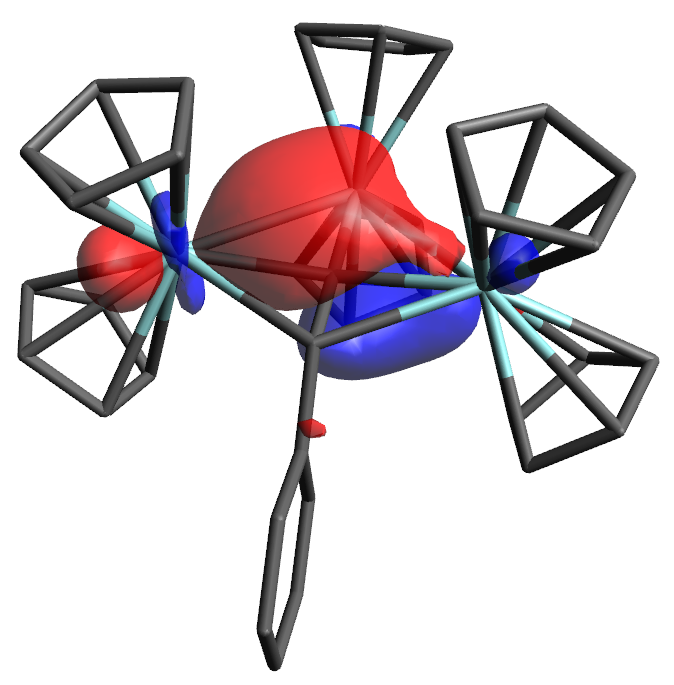 | 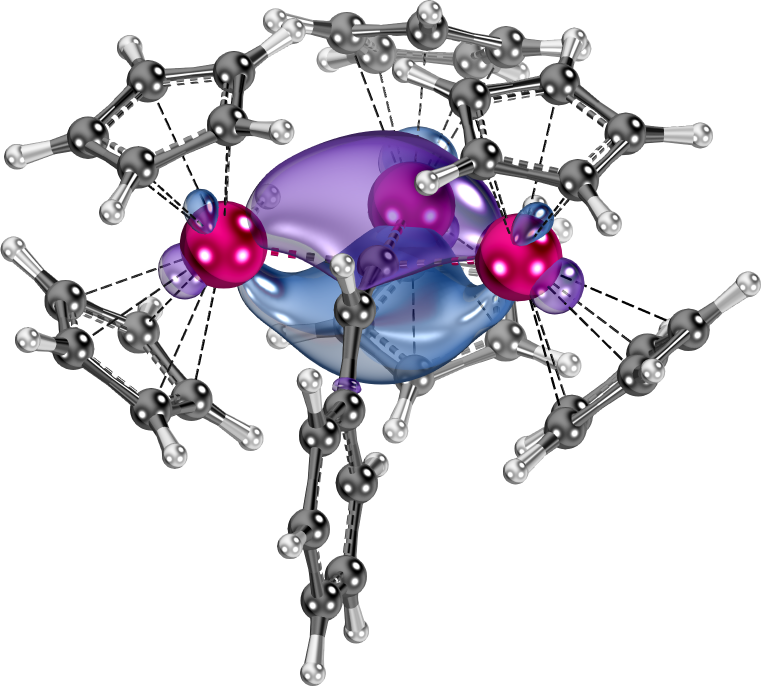 |
| Shortened NLMO 60 Analysis of C(1)-Zr(75) π-bond indicates also contributions by the bridged Zr(74,76) atoms (threshold > 1.3%)  60. (2.00000) 76.0335% BD ( 2) C 1-Zr 75  63.363% C 1 s( 5.50%)p17.19( 94.46%)d 0.00( 0.01%) f 0.01( 0.03%)  13.156% Zr 74 s( 0.86%)p 0.17( 0.14%)d99.99( 98.89%) f 0.13( 0.11%)  12.805% Zr 75 s( 1.23%)p 0.09( 0.11%)d80.08( 98.54%) f 0.10( 0.13%)  5.897% Zr 76 s( 0.61%)p 0.43( 0.26%)d99.99( 98.86%) | pi out off plane bond  Sel. Desc.  0.037 151.1 [E = -0.2177 O =AB]  Centres/Charges  C 1 1.746  ZR 75 0.057  C 2 0.037  ZR 74 0.036  ZR 76 0.035  H 77 0.021 (other: 0.068) |
| 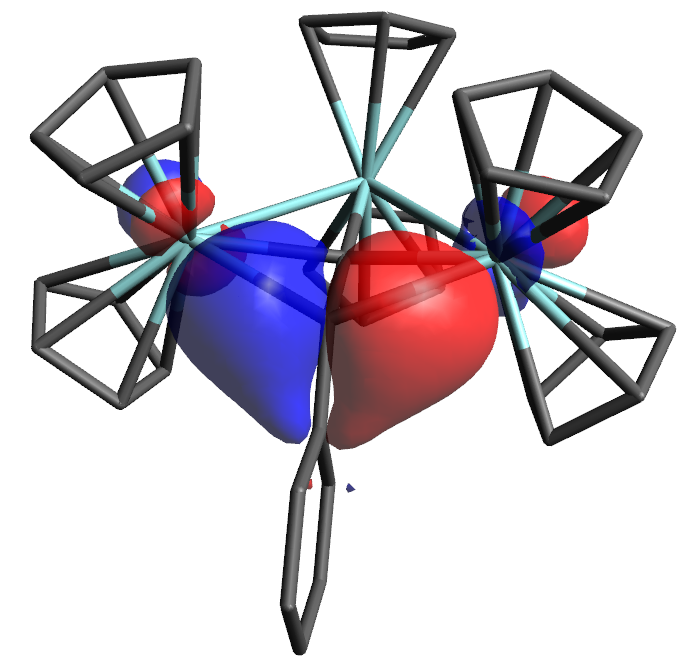 | 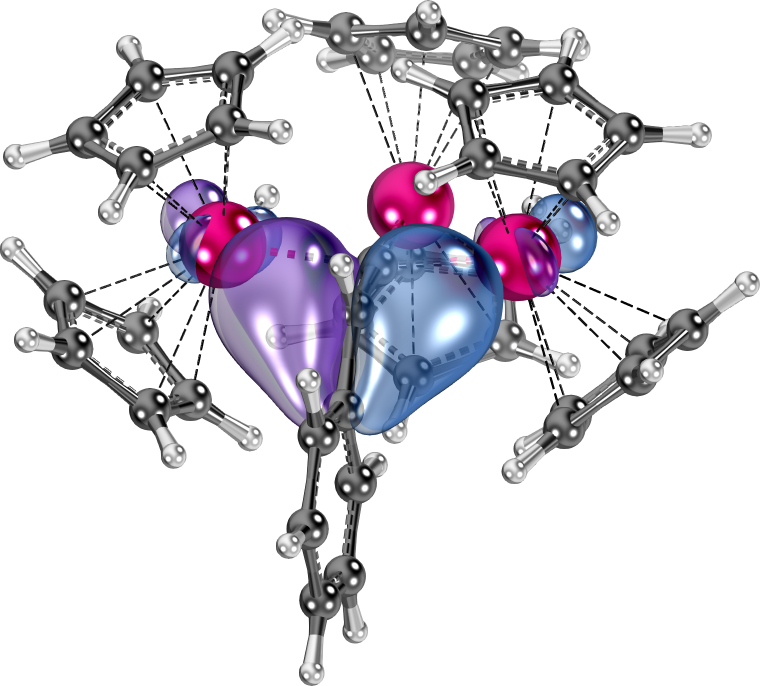 |
| Shortened NLMO 63 Analysis of C(2)-Zr(76) σ-bond reveal almost equal contributions of both bridged Zr(74,76) atoms (threshold > 1.3%)  63. (2.00000) 78.4299% BD ( 1) C 2-Zr 76  1.544% C 1 s( 0.00%)p 1.00( 95.72%)d 0.04( 4.26%) f 0.00( 0.02%)  67.212% C 2 s( 0.23%)p99.99( 99.67%)d 0.36( 0.08%) f 0.07( 0.02%)  3.355% C 3 s( 0.01%)p 1.00( 99.40%)d 0.01( 0.57%)f 0.00( 0.03%)  9.742% Zr 74 s( 4.02%)p 0.06( 0.24%)d23.78( 95.70%) f 0.01( 0.05%)  1.282% Zr 75 s( 0.04%)p24.56( 0.98%)d99.99( 98.91%)f 1.53( 0.06%)  11.564% Zr 76 s( 4.77%)p 0.05( 0.22%)d19.91( 94.96%) f 0.01( 0.05%) | Sel. Desc.  1.599* 149.1 [E = -0.2814 O =AB]  Centres/Charges  C 2 1.599  C 3 0.133  ZR 76 0.077  ZR 74 0.075 (other: 0.116) |
| 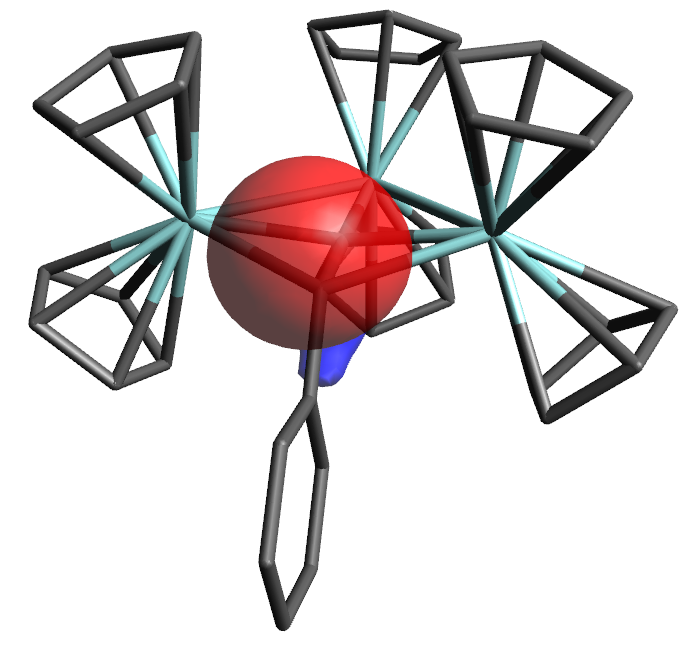 | 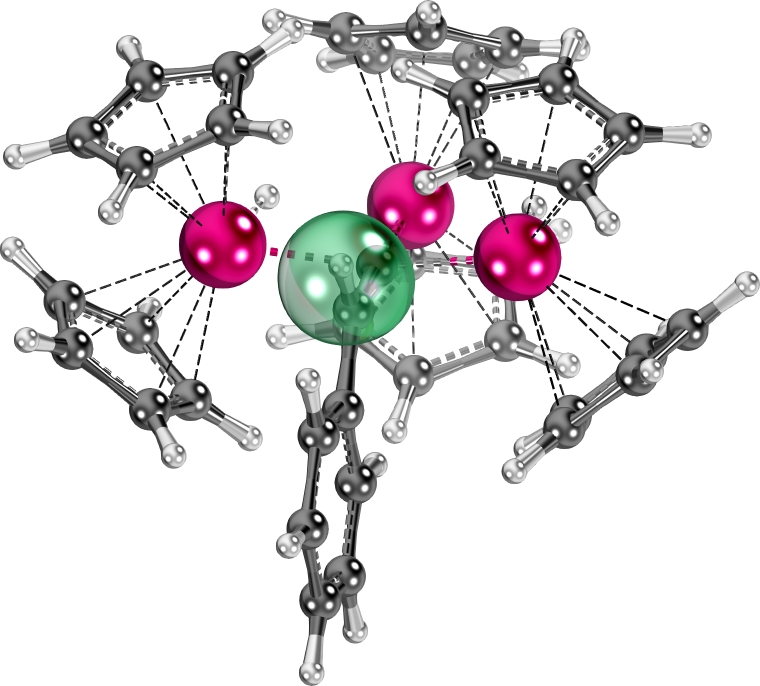 |
| Shortened NLMO 64 Analysis of C(2)-H(77) σ-bond show nearly negliglible contributions by the bridges Zr(74,76) atoms (threshold > 1.3%)  64. (2.00000) 95.0120% BD ( 1) C 2- H 77  58.713% C 2 s( 24.11%)p 3.14( 75.83%)d 0.00( 0.05%)f 0.00( 0.01%)  1.854% Zr 74 s( 15.99%)p 0.02( 0.29%)d 5.22( 83.51%)f 0.01( 0.21%)  1.335% Zr 76 s( 16.25%)p 0.02( 0.35%)d 5.12( 83.11%)f 0.02( 0.29%)  36.378% H 77 s( 99.89%)p 0.00( 0.11%) | Sel. Desc.  1.093 119.1 [E = -0.5312 O =AB]  Centres/Charges  C 2 1.093  H 77 0.872 (other: 0.035) |
| 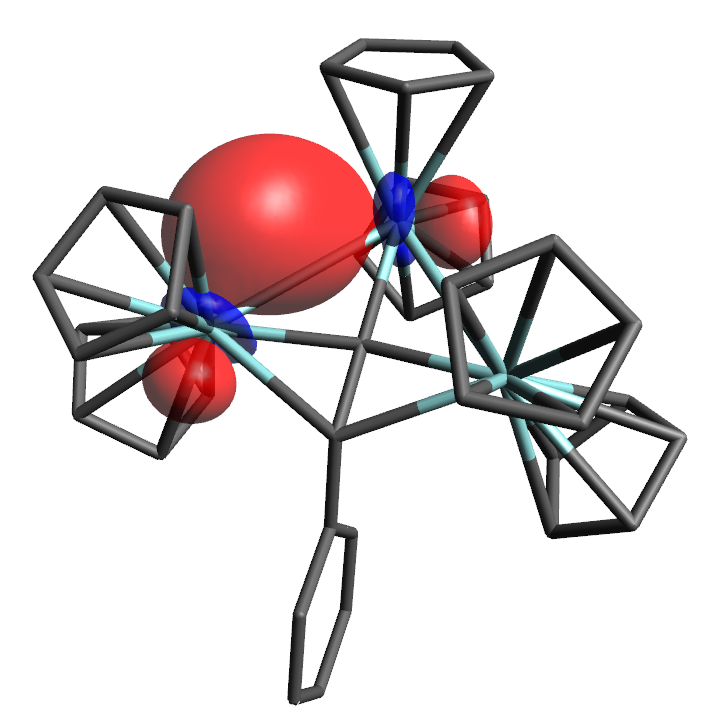 | 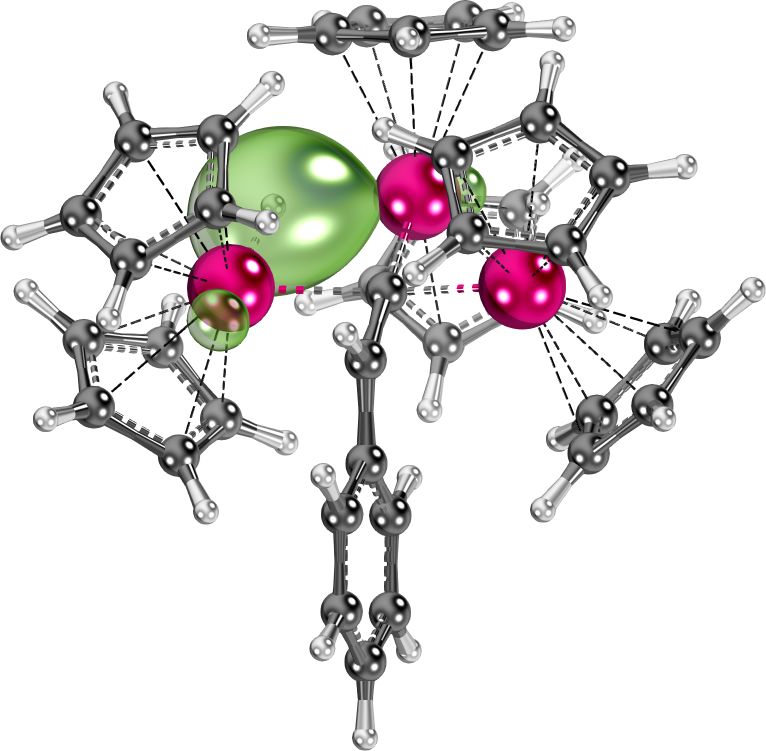 |
| Shortened NLMO 150 Analysis of H(79)-Zr(74) σ-bond reveal the hydride(79) bridge to the Zr(74,75) atoms (threshold > 1.3%)  150. (2.00000) 81.6077% BD ( 1)Zr 74- H 79  19.518% Zr 74 s( 12.58%)p 0.02( 0.31%)d 6.92( 87.02%)f 0.01( 0.09%)  15.362% Zr 75 s( 11.71%)p 0.01( 0.17%)d 7.51( 88.00%)f 0.01( 0.12%)  62.105% H 79 s( 99.90%)p 0.00( 0.10%) | Sel. Desc.  1.693* 127.1 [E = -0.3455 O =AB]  Centres/Charges  H 79 1.693  ZR 74 0.154  ZR 75 0.114 (other: 0.039) |
| 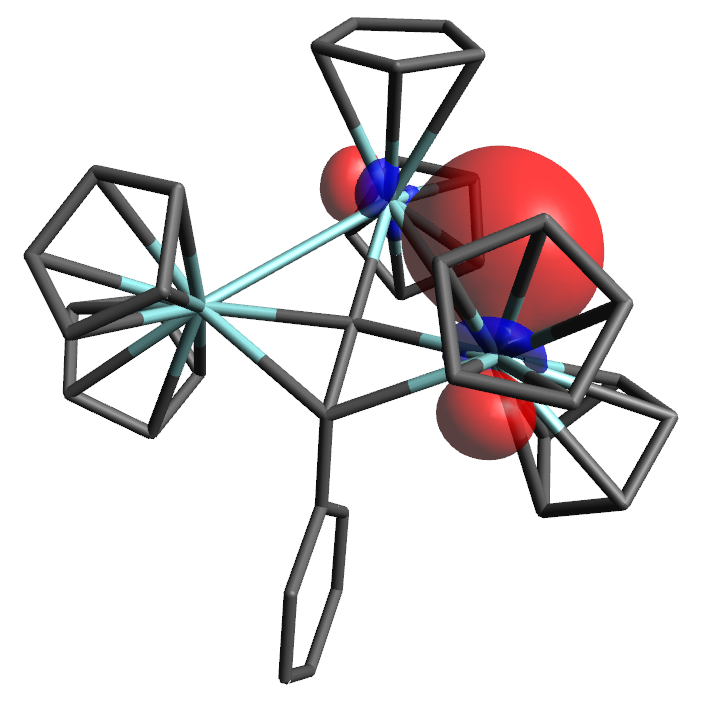 | 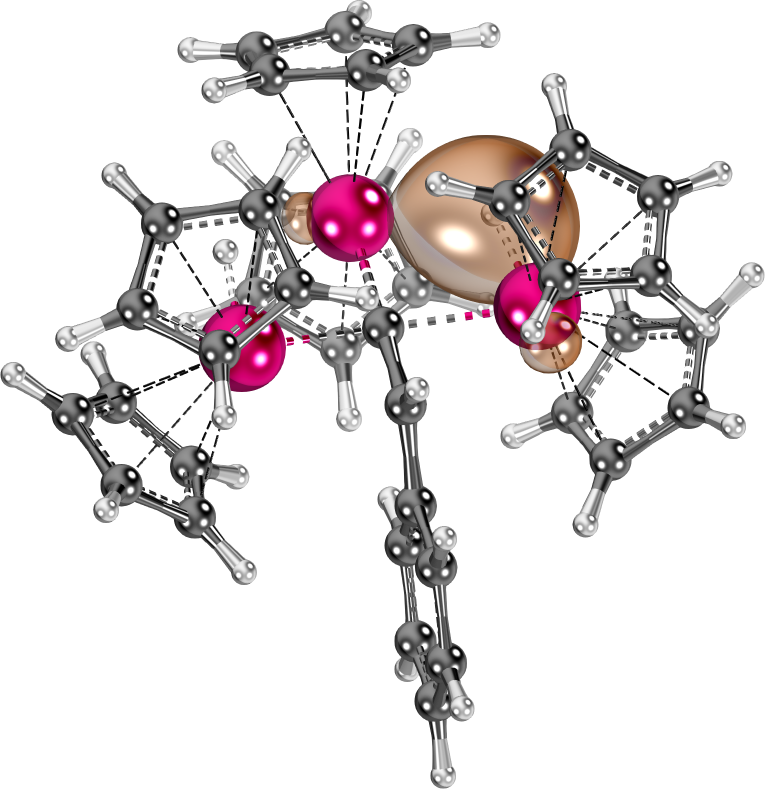 |
| Shortened NLMO 151 Analysis of H(78)-Zr(76) σ-bond reveal the hydride(78) bridge to the Zr(75,76) atoms (threshold > 1.3%)  151. (2.00000) 82.5553% BD ( 1)Zr 76- H 78  13.110% Zr 75 s( 10.44%)p 0.03( 0.26%)d 8.54( 89.15%)f 0.01( 0.15%)  20.279% Zr 76 s( 11.21%)p 0.03( 0.30%)d 7.88( 88.40%) f 0.01( 0.09%)  62.399% H 78 s( 99.90%)p 0.00( 0.10%) | Sel. Desc.  1.693* 128.1 [E = -0.3452 O =AB]  Centres/Charges  H 78 1.693  ZR 76 0.155  ZR 75 0.113 (other: 0.039) |

#### QT-AIM analysis of (Cp_2_Zr)_3_(µ-H_2_)(σ-µ-C=CHPh) (**7**)


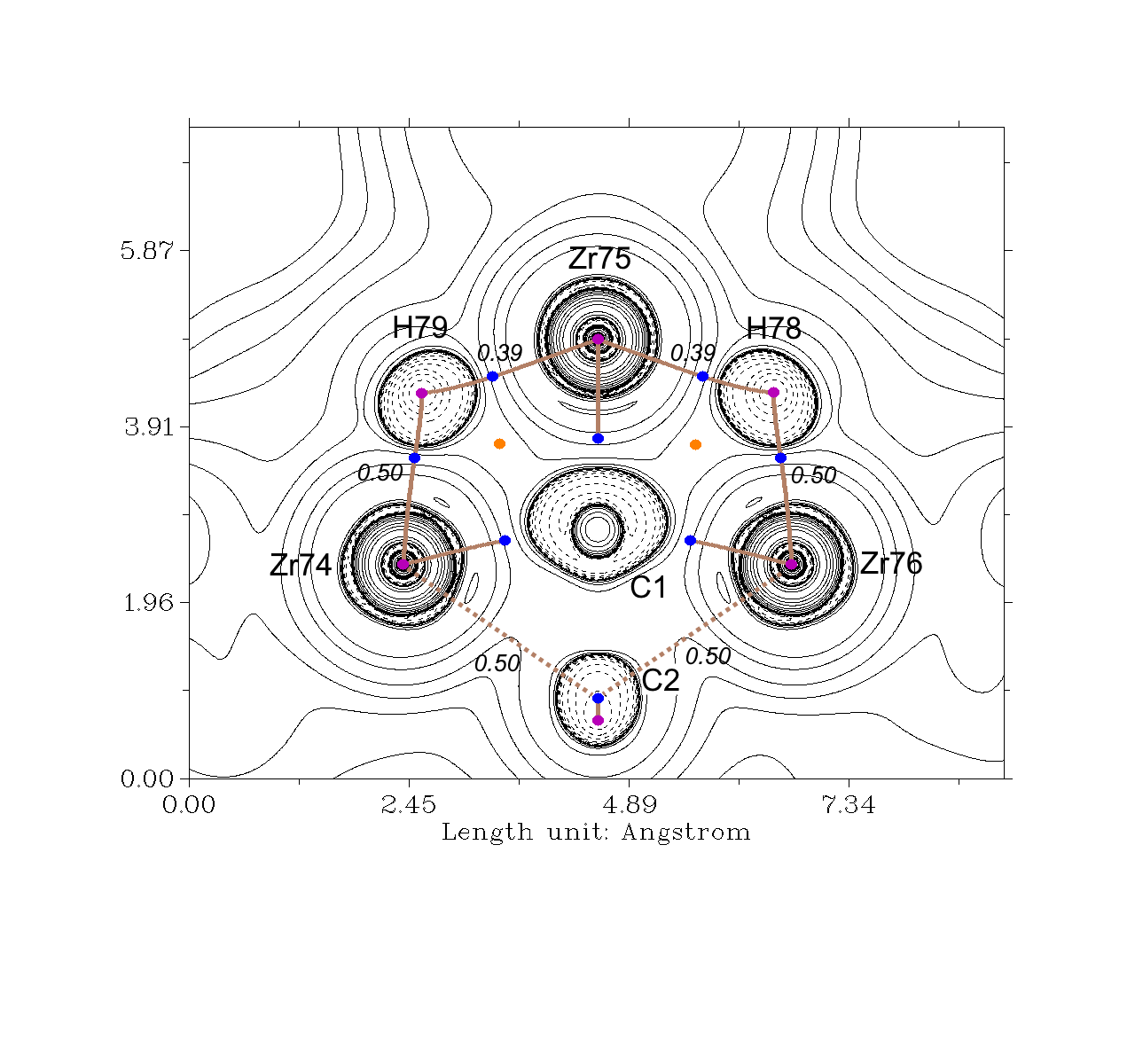


**Figure S17.** Contour plot of the Laplacian of the electron density ∇^2^*r* of complex **7** in the Zr74-Zr76-Zr75 plane. Dashed lines indicate negative (local charge concentration), solid lines indicate positive values (local charge depletion). The Laplacian plot is overlaid with the molecular graph from QT-AIM analysis and Wiberg bond indices (italic small numbers). Brown lines indicate bond paths, brown dashed lines are hypothetical bonds, blue dots correspond to bond critical points, light brown dots indicate ring critical points. Density from B3LYP-D3/def2-TZVP calculation.


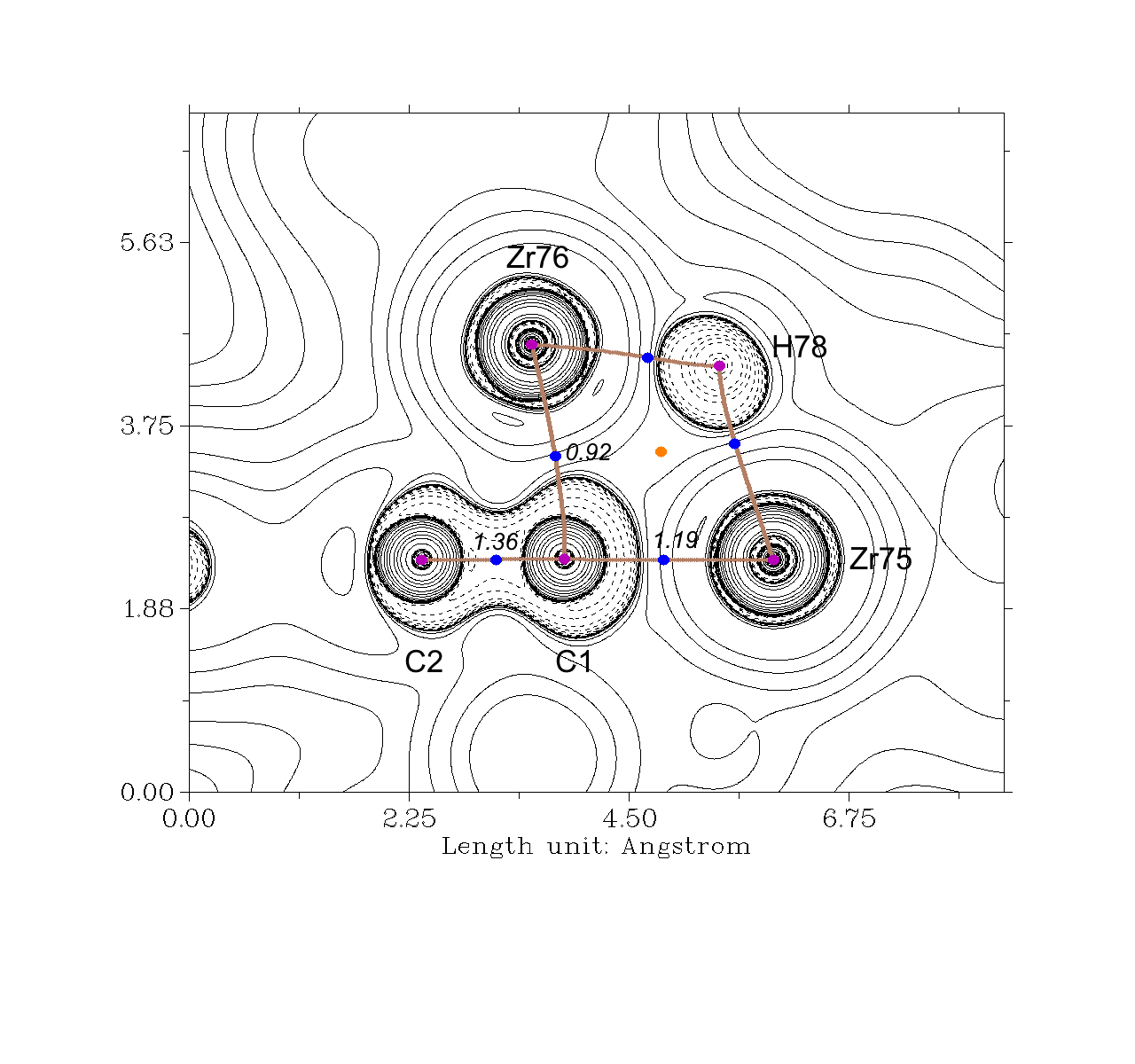


**Figure S18.** Contour plot of the Laplacian of the electron density ∇^2^*r* of complex **7** in the C2-Zr75-Zr76 plane. Dashed lines indicate negative (local charge concentration), solid lines indicate positive values (local charge depletion). The Laplacian plot is overlaid with the molecular graph from QT-AIM analysis and Wiberg bond indices (italic small numbers). Brown lines indicate bond paths, brown dashed lines are hypothetical bonds, blue dots correspond to bond critical points, light brown dots indicate ring critical points. Density from B3LYP-D3/def2-TZVP calculation.


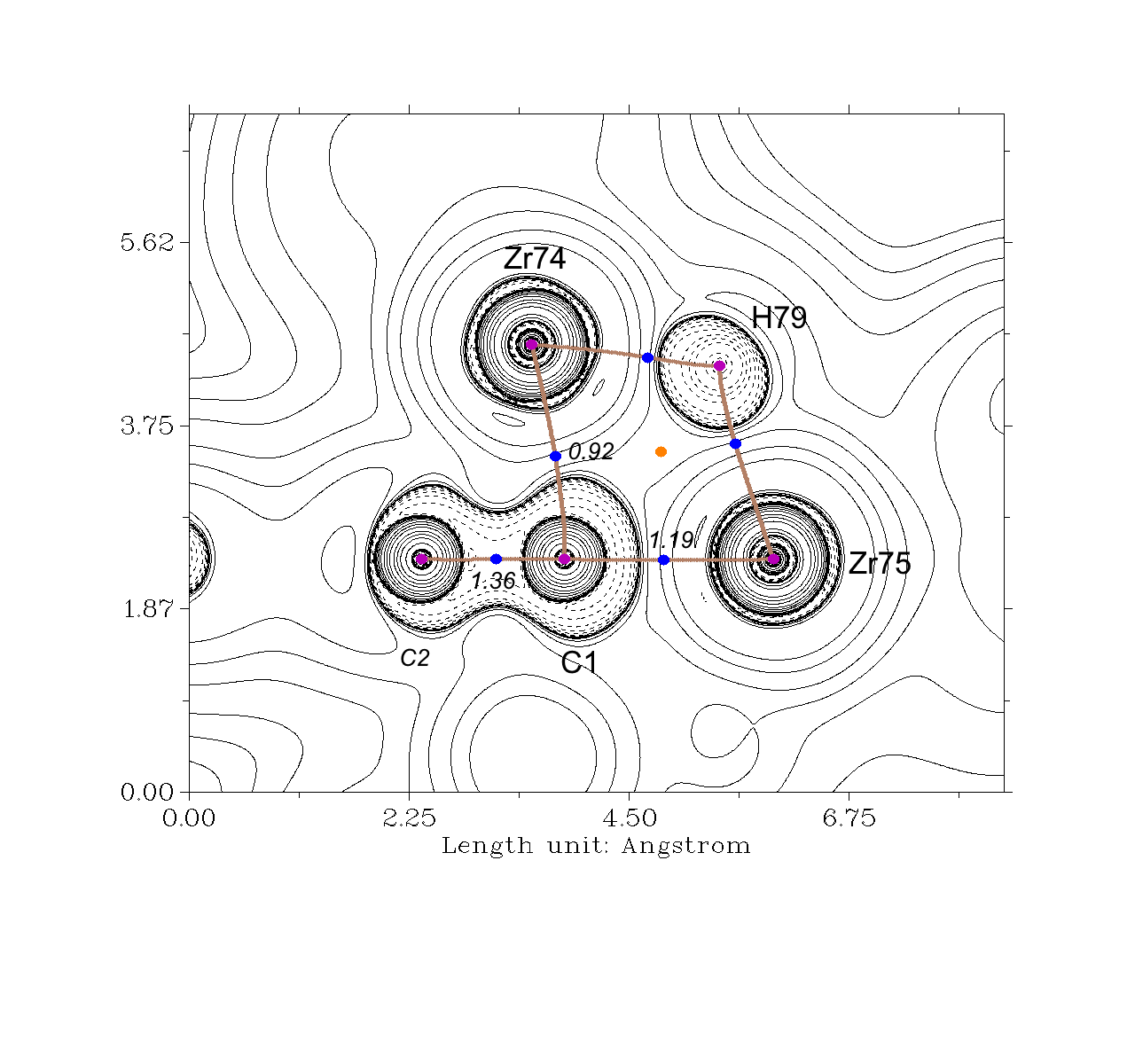


**Figure S19.** Contour plot of the Laplacian of the electron density ∇^2^*r* of complex **7** in the C2-Zr75-Zr74 plane. Dashed lines indicate negative (local charge concentration), solid lines indicate positive values (local charge depletion). The Laplacian plot is overlaid with the molecular graph from QT-AIM analysis and Wiberg bond indices (italic small numbers). Brown lines indicate bond paths, brown dashed lines are hypothetical bonds, blue dots correspond to bond critical points, light brown dots indicate ring critical points. Density from B3LYP-D3/def2-TZVP calculation.

#### Interaction region indicator (IRI) analysis of (Cp_2_Zr)_3_(µ-H_2_)(σ-µ-C=CHPh) (**7**)

For a better understanding of the unique bond situation in complex **7** in addition to the former analysis, an IRI analysis^19^ was carried out based on the calculated structures. This analysis clearly shows large areas of attractive van der Waals interactions between the cyclopentadienyl units and between the lower Cp units and the Ph substituent (green surfaces). The two three-center two-electron [Zr-H-Zr] bonding interactions are adequately represented by the blue surfaces between both Zr atoms and the hydride atoms involved. The central carbon atom C1 is bound to C2 and shows bonding interactions to all Zr atoms. This analysis also highlights the bonding interaction between C2 and the two side-on coordinated Zr centres.


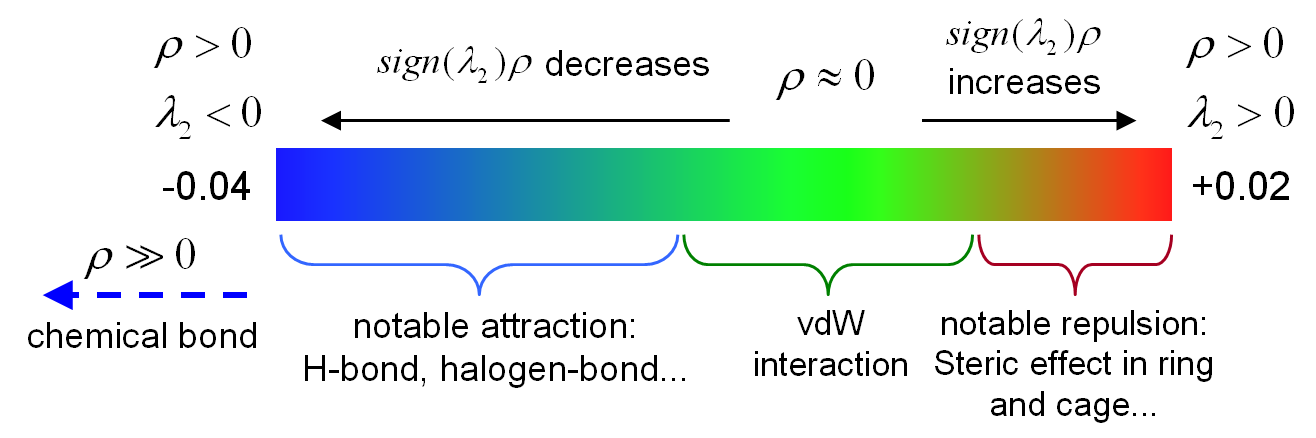


**Table S9.** Selected representations of the IRI analysis of **7**.

| Front view |
| --- |
| 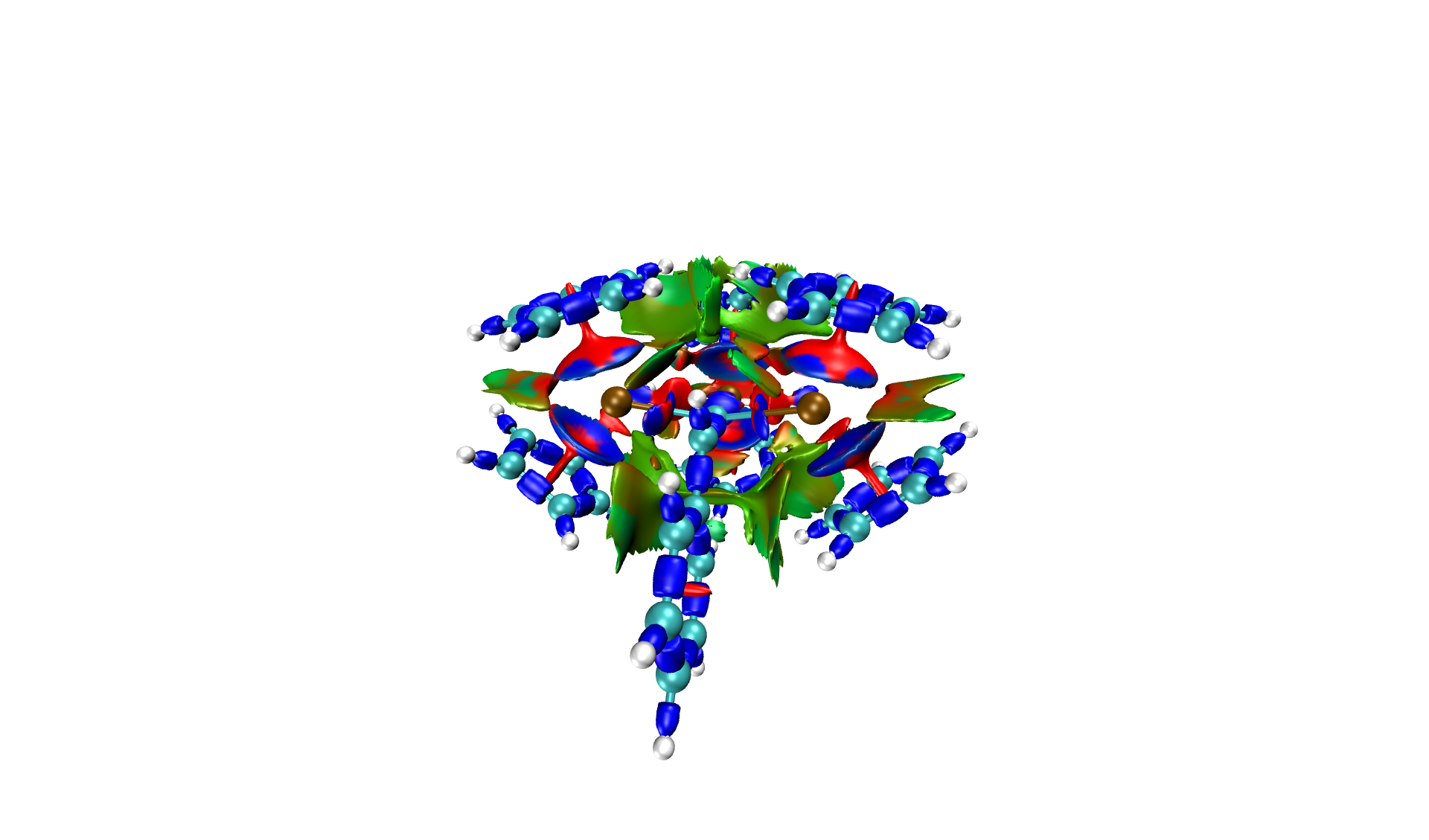 |
| Top view |
| 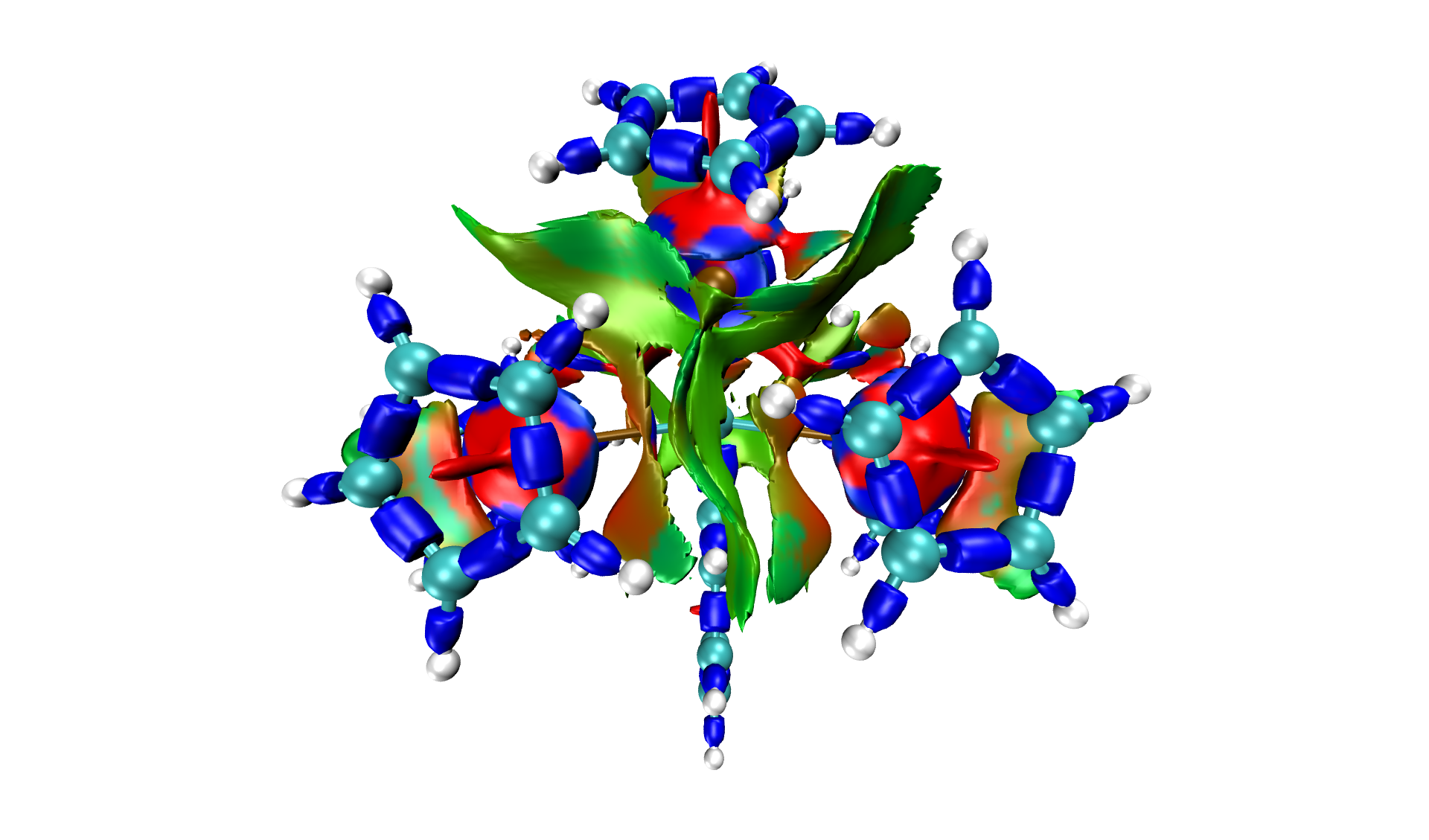 |
| Bottom view. |
| 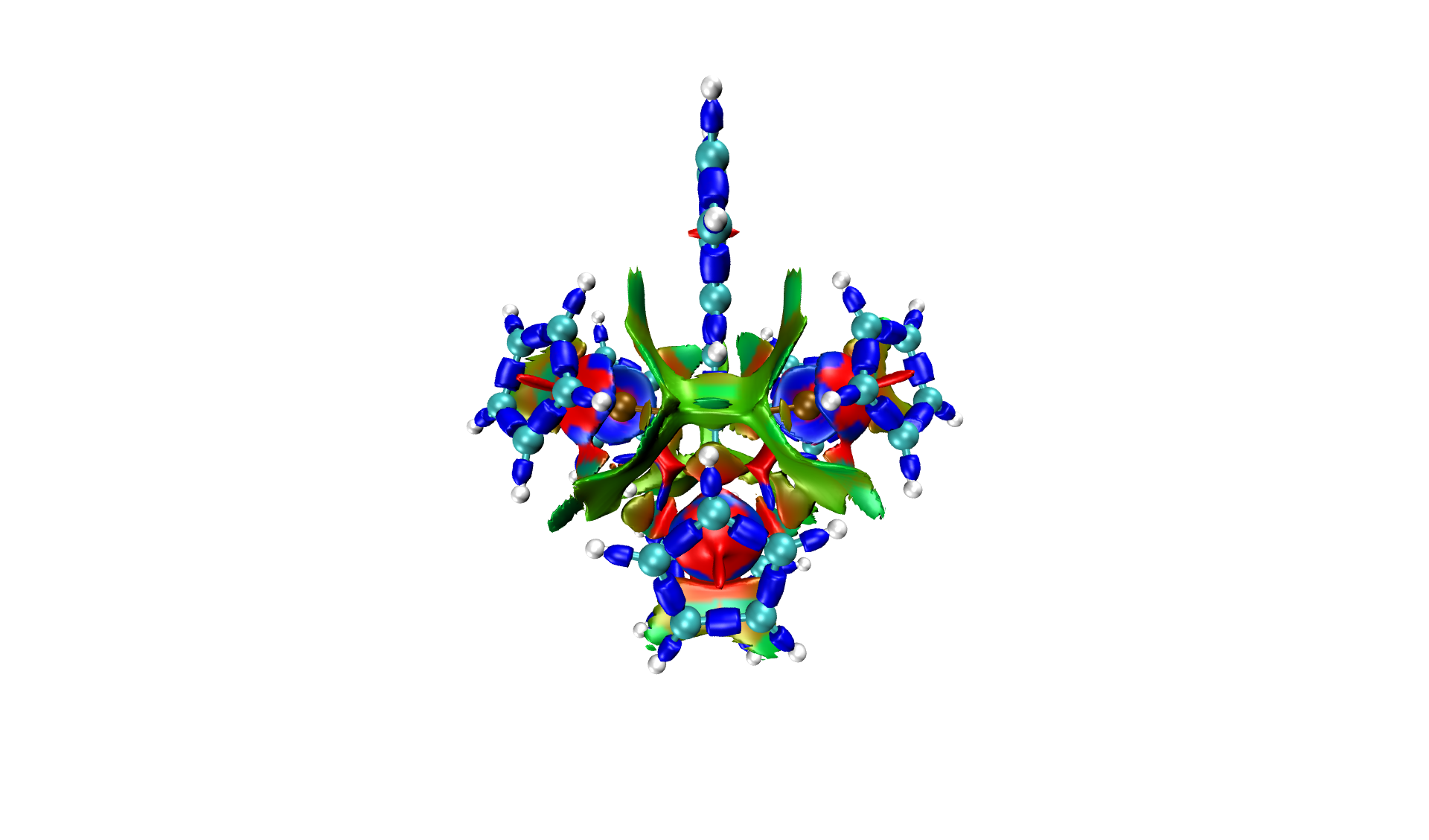 |
| Side view to visualise Zr1H1Zr2 bonding interaction |
| 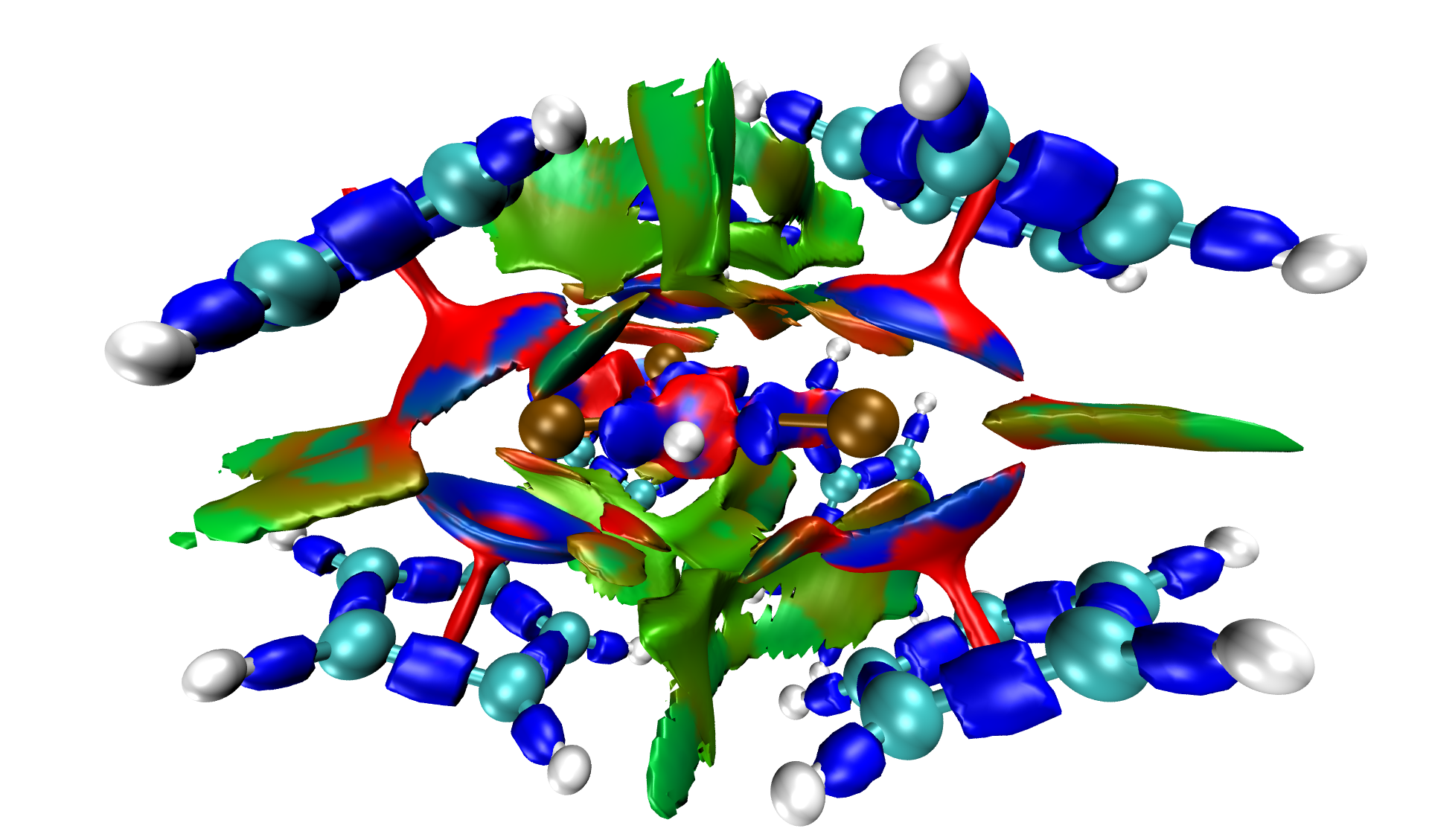 |
| Side view to visualise Zr2H2Zr3 bonding interaction |
| 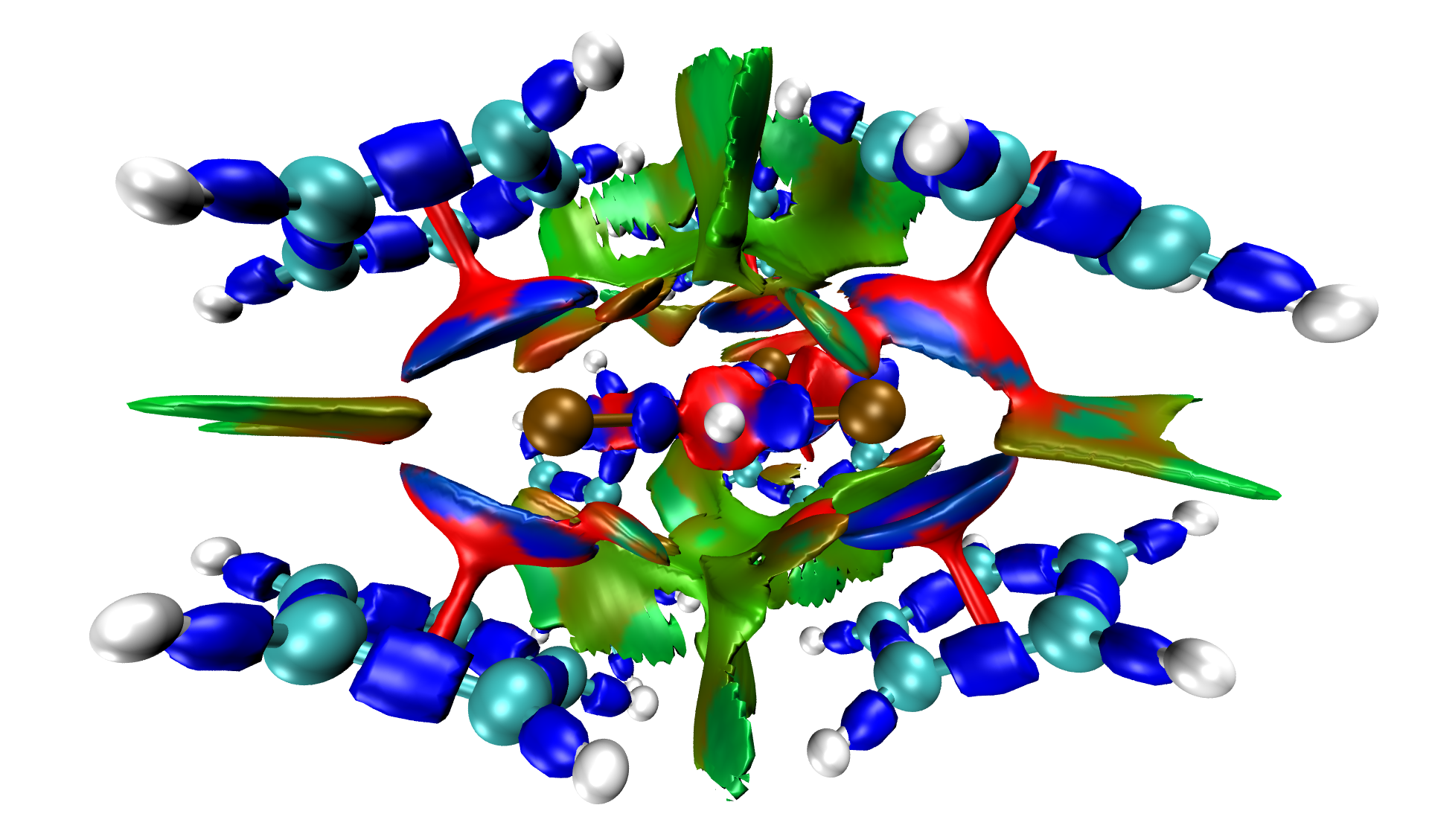 |
| Magnification, highlighting the central unit of the molecule |
| 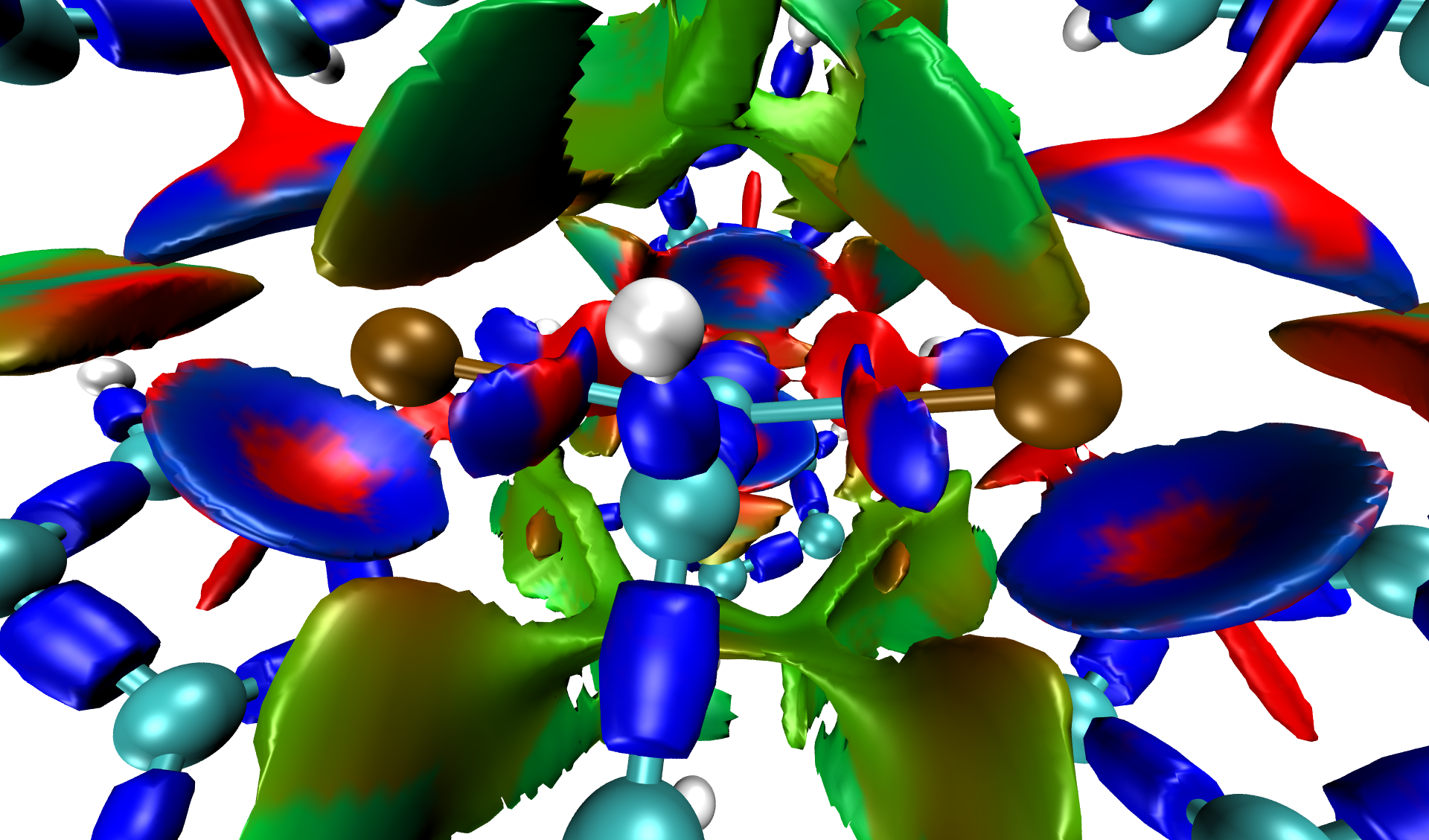 |

### **Bond analysis of** **(Cp_2_Zr)_2_(µ-CN-Py)(σ-C_2_Ph) (8)**


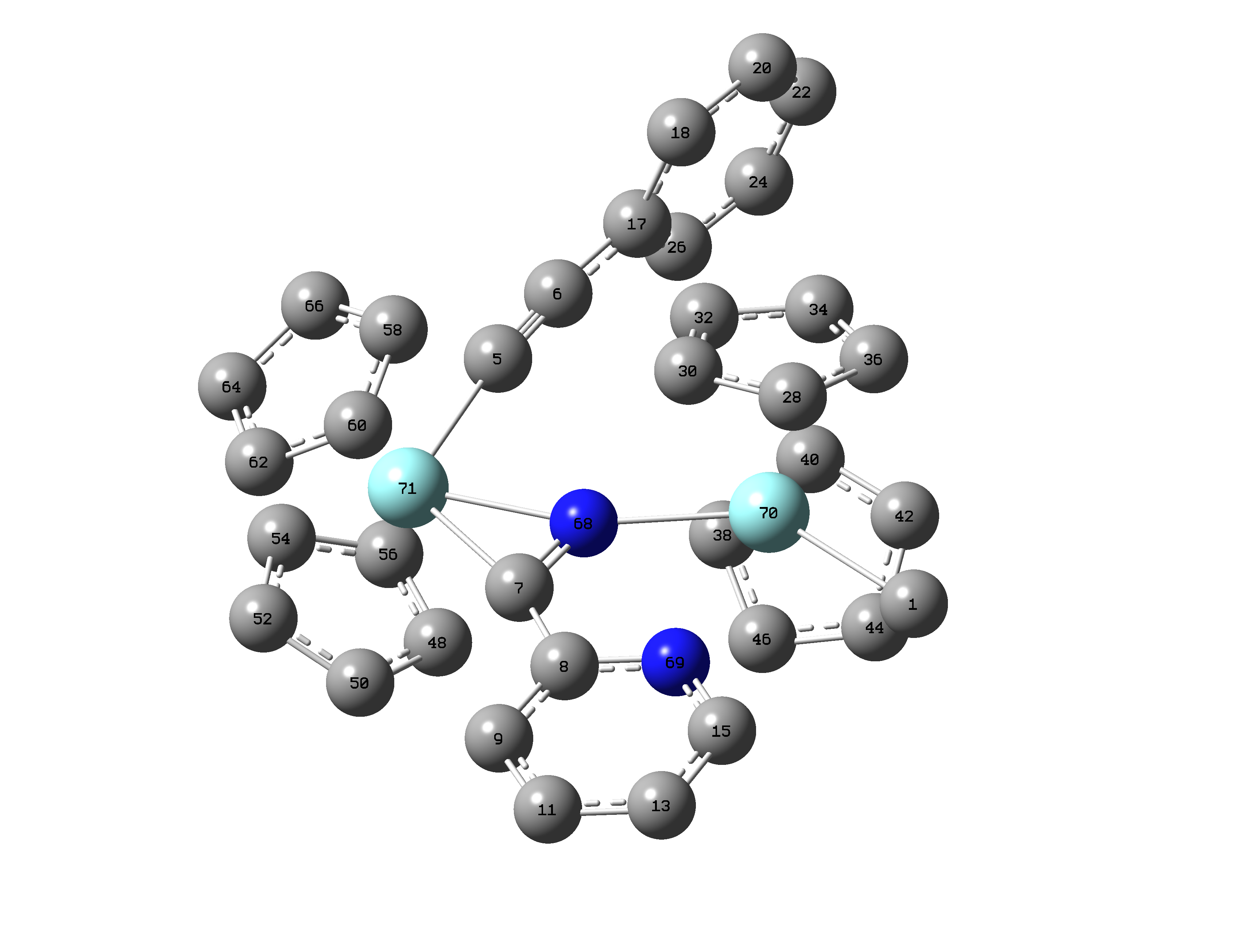


**Figure S20.** Labelling scheme for the bond analysis of complex **8**. Hydrogen atoms are omitted for clarity.

#### NBO/NLMO analysis of (Cp_2_Zr)_2_(CN-Py)Ph (**8**)

For this purpose, the molecule was divided into five logical units and the natural charges of the atoms of these were summed up.

**Table S10.** Summary of selected NBO analysis of **8**.

| 52. (1.77037) LP ( 1) N 68  s( 46.99%)p 1.13( 52.90%)d 0.00( 0.11%) f 0.00( 0.00%) | 53. (1.65107) LP ( 2) N 68  s( 4.31%)p22.17( 95.55%)d 0.03( 0.14%) f 0.00( 0.01%) |
| --- | --- |
| 54. (1.80988) LP ( 1) N 69  s( 27.78%)p 2.60( 72.17%)d 0.00( 0.05%) f 0.00( 0.00%) | 58. (1.96250) BD ( 1) C 1-Zr 70  ( 81.50%) 0.9028* C 1 s( 32.02%)p 2.12( 67.92%)d 0.00( 0.05%) f 0.00( 0.01%)  ( 18.50%) 0.4301*Zr 70 s( 12.76%)p 0.02( 0.24%)d 6.81( 86.93%) f 0.01( 0.07%) |
| 59. (1.98243) BD ( 1) C 5- C 6  ( 45.81%) 0.6769* C 5 s( 44.36%)p 1.25( 55.41%)d 0.00( 0.22%) f 0.00( 0.02%)  ( 54.19%) 0.7361* C 6 s( 54.50%)p 0.83( 45.37%)d 0.00( 0.06%) f 0.00( 0.07%) | 60. (1.94198) BD ( 2) C 5- C 6  ( 47.08%) 0.6862* C 5 s( 0.00%)p 1.00( 99.92%)d 0.00( 0.06%) f 0.00( 0.03%)  ( 52.92%) 0.7274* C 6 s( 0.00%)p 1.00( 99.84%)d 0.00( 0.04%) f 0.00( 0.12%) |
| 61. (1.88021) BD ( 3) C 5- C 6  ( 45.44%) 0.6741* C 5 s( 0.01%)p 1.00( 99.92%)d 0.00( 0.05%) f 0.00( 0.02%)  ( 54.56%) 0.7386* C 6 s( 0.00%)p 1.00( 99.84%)d 0.00( 0.03%) f 0.00( 0.12%) | 62. (1.95398) BD ( 1) C 5-Zr 71  ( 80.41%) 0.8967* C 5 s( 55.23%)p 0.81( 44.73%)d 0.00( 0.03%) f 0.00( 0.01%)  ( 19.59%) 0.4426*Zr 71 s( 13.73%)p 0.00( 0.07%)d 6.28( 86.13%) f 0.01( 0.07%) |
| 65. (1.95566) BD ( 1) C 7- N 68  ( 41.39%) 0.6434* C 7 s( 30.86%)p 2.24( 68.99%)d 0.00( 0.12%) f 0.00( 0.03%)  ( 58.61%) 0.7656* N 68 s( 48.59%)p 1.05( 50.99%)d 0.01( 0.42%) f 0.00( 0.01%) | 66. (1.87646) BD ( 2) C 7- N 68  ( 39.01%) 0.6246* C 7 s( 0.00%)p 1.00( 99.77%)d 0.00( 0.19%) f 0.00( 0.04%)  ( 60.99%) 0.7810* N 68 s( 0.00%)p 1.00( 99.76%)d 0.00( 0.23%) f 0.00( 0.02%) |
| 67. (1.89732) BD ( 1) C 7-Zr 71  ( 77.60%) 0.8809* C 7 s( 33.61%)p 1.97( 66.33%)d 0.00( 0.05%) f 0.00( 0.01%)  ( 22.40%) 0.4732*Zr 71 s( 5.22%)p 0.02( 0.09%)d18.12( 94.60%) f 0.02( 0.09%) | 69. (1.97665) BD ( 1) C 8- N 69  ( 41.08%) 0.6409* C 8 s( 31.09%)p 2.21( 68.79%)d 0.00( 0.09%) f 0.00( 0.03%)  ( 58.92%) 0.7676* N 69 s( 35.20%)p 1.83( 64.46%)d 0.01( 0.33%) f 0.00( 0.02%) |
|  |  |

**Table S11.** Summary of selected NLMOs of **8** (iso0.04).

| 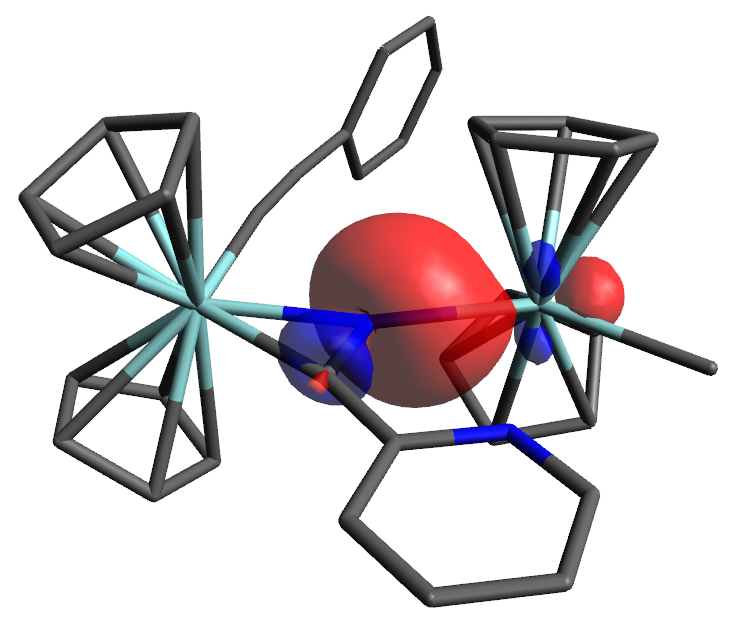 | 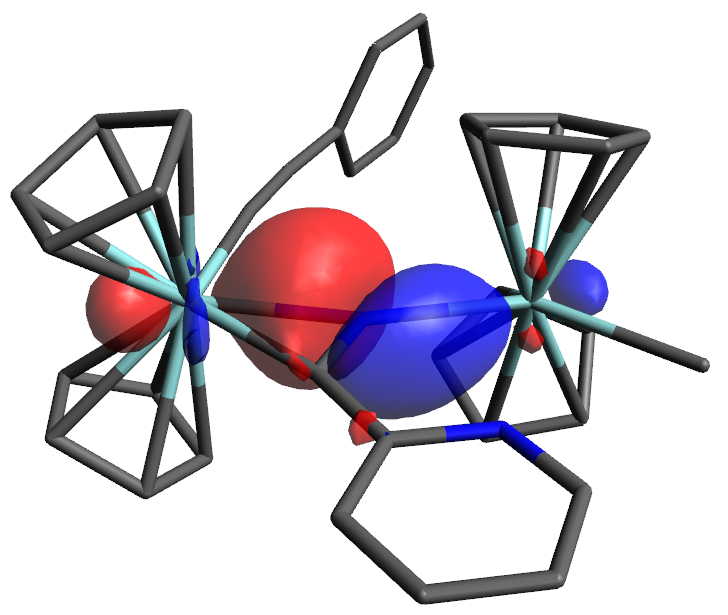 |
| --- | --- |
| Shortened NLMO 52 Analysis of LP (1) N(68) indicates a strongly polarised binding feature to to Zr(70) next to small contributions by Zr(71) atom (threshold > 1.3%)  52. (2.00000) 87.7237% LP ( 1) N 68  87.835% N 68 s( 48.30%)p 1.07( 51.60%)d 0.00( 0.10%) f 0.00( 0.00%)  8.706% Zr 70 s( 16.91%)p 0.01( 0.19%)d 4.89( 82.69%) f 0.01( 0.21%)  1.722% Zr 71 s( 33.82%)p 0.03( 1.11%)d 1.92( 64.84%) f 0.01( 0.22%) | Shortened NLMO 53 Analysis of LP (2) N(68) indicates a strongly polarised binding feature to to Zr(71) next to small contributions by Zr(70) atom (threshold > 1.3%)  53. (2.00000) 81.4464% LP ( 2) N 68  2.151% C 7 s( 0.54%)p99.99( 96.95%)d 4.30( 2.31%) f 0.37( 0.20%)  81.546% N 68 s( 5.80%)p16.21( 94.05%)d 0.02( 0.14%) f 0.00( 0.01%)  1.821% Zr 70 s( 2.77%)p 0.22( 0.62%)d34.72( 96.08%) f 0.19( 0.54%)  11.228% Zr 71 s( 4.64%)p 0.03( 0.15%)d20.47( 95.06%) f 0.03( 0.14%) |
| 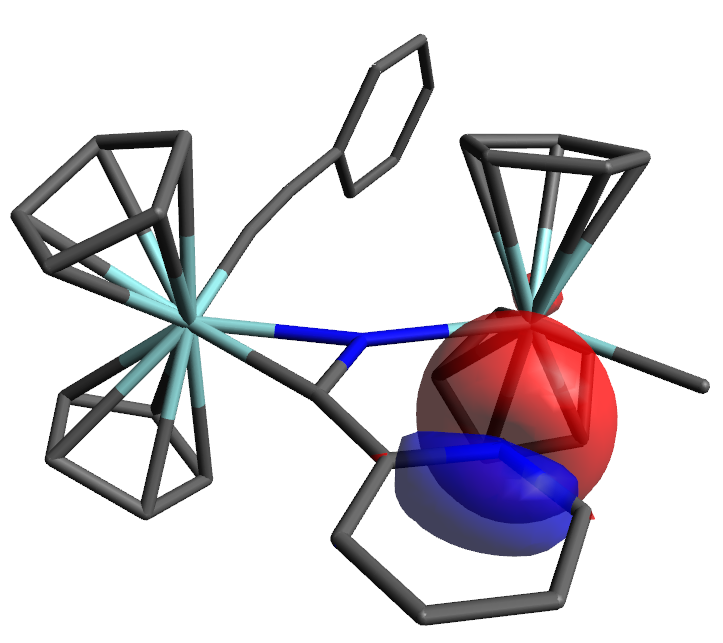 | 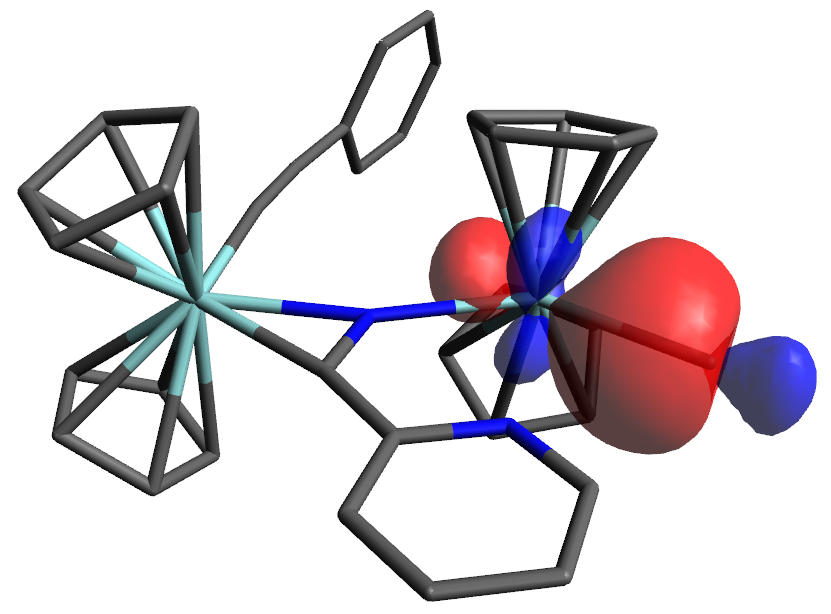 |
| Shortened NLMO 54 Analysis of LP N(69) indicates a strongly polarised binding feature to to Zr(70) (threshold > 1.3%)  54. (2.00000) 90.2227% LP ( 1) N 69  90.228% N 69 s( 28.43%)p 2.52( 71.52%)d 0.00( 0.05%) f 0.00( 0.00%)  6.064% Zr 70 s( 24.60%)p 0.01( 0.27%)d 3.05( 74.92%) f 0.01( 0.21%) | Shortened NLMO 58 Analysis of C(1)-Zr(70) ) σ -bond (threshold > 1.3%)  58. (2.00000) 98.1126% BD ( 1) C 1-Zr 70  79.932% C 1 s( 29.73%)p 2.36( 70.22%)d 0.00( 0.05%) f 0.00( 0.01%)  18.236% Zr 70 s( 12.53%)p 0.02( 0.24%)d 6.96( 87.16%) f 0.01( 0.07%) |
| 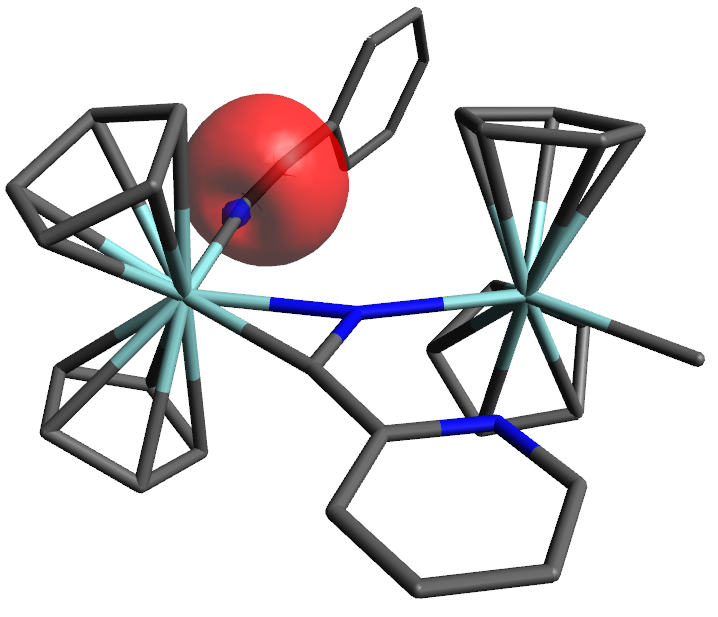 | 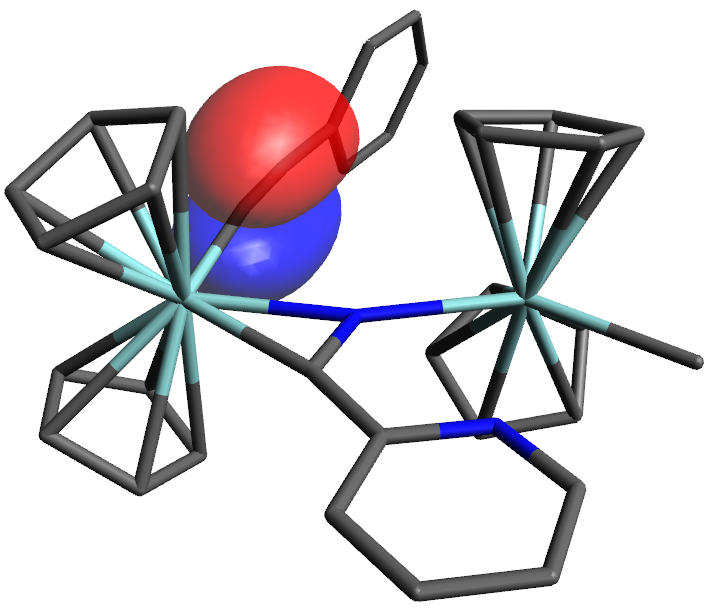 |
| Shortened NLMO 59 Analysis of C(5)-C(6) σ-bond reveal no contributions of the Zr atoms (threshold > 1.3%)  59. (2.00000) 99.1168% BD ( 1) C 5- C 6  45.447% C 5 s( 41.49%)p 1.40( 58.28%)d 0.01( 0.22%) f 0.00( 0.02%)  53.916% C 6 s( 48.33%)p 1.07( 51.54%)d 0.00( 0.05%) f 0.00( 0.08%) | Shortened NLMO 60 Analysis of C(5)-C(6) π-bond reveal only weak binding contributions of the Zr(71) atom (threshold > 1.3%)  60. (2.00000) 97.0363% BD ( 2) C 5- C 6  45.642% C 5 s( 0.00%)p 1.00( 99.92%)d 0.00( 0.06%) f 0.00( 0.03%)  51.395% C 6 s( 0.00%)p 1.00( 99.84%)d 0.00( 0.04%) f 0.00( 0.12%)  1.305% Zr 71 s( 0.01%)p50.00( 0.58%)d99.99( 98.77%) f54.63( 0.64%) |
| 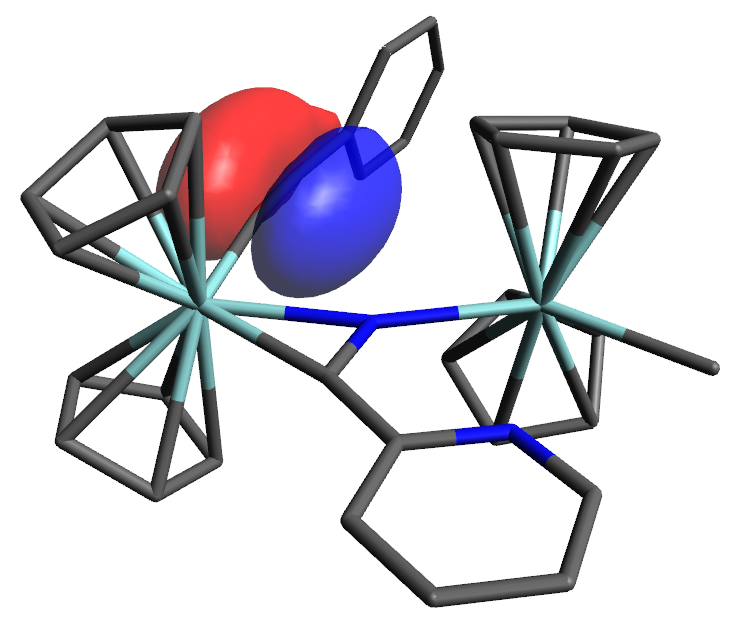 | 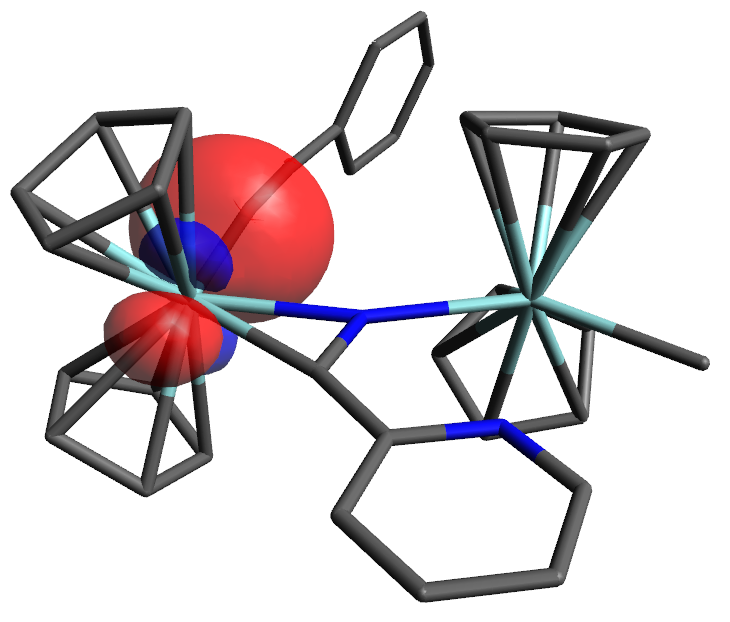 |
| Shortened NLMO 61 Analysis of C(5)-C(6) π-bond reveal no bridging feature of this in plane π-bond to the Zr(70) atom (threshold > 1.3%)  61. (2.00000) 93.9437% BD ( 3) C 5- C 6  42.529% C 5 s( 0.02%)p99.99( 99.91%)d 3.38( 0.05%) f 1.48( 0.02%)  51.427% C 6 s( 0.01%)p 1.00( 99.84%)d 0.00( 0.03%) f 0.00( 0.12%)  2.476% C 17 s( 0.02%)p99.99( 99.38%)d36.35( 0.56%) f 3.24( 0.05%) | Shortened NLMO 62 Analysis of C(5)-Zr(71) σ-bond (threshold > 1.3%)  62. (2.00000) 97.6846% BD ( 1) C 5-Zr 71  78.683% C 5 s( 52.13%)p 0.92( 47.82%)d 0.00( 0.03%) f 0.00( 0.01%)  19.234% Zr 71 s( 15.55%)p 0.00( 0.06%)d 5.42( 84.32%) f 0.00( 0.07%) |
| 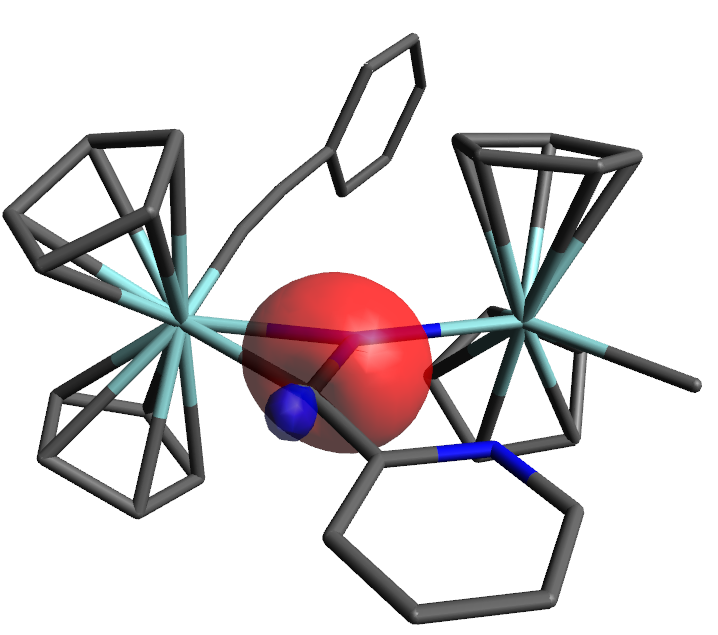 | 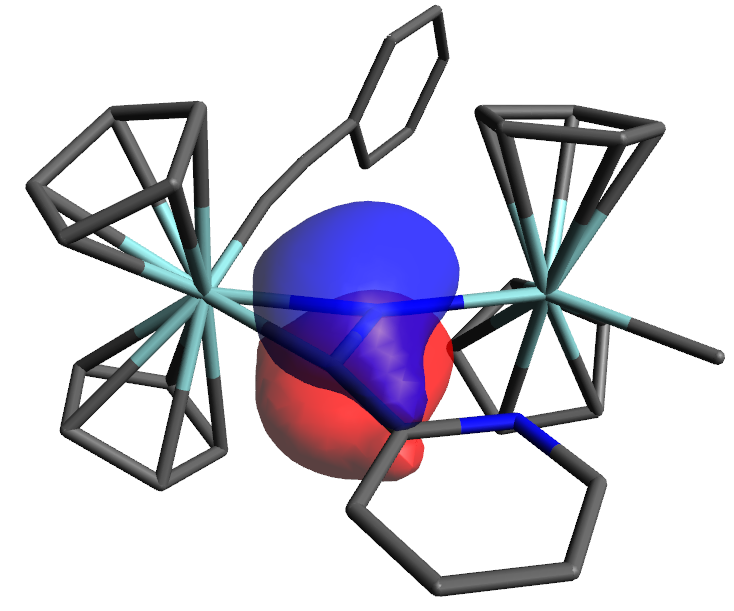 |
| Shortened NLMO 65 Analysis of C(7)-N(68) σ-bond reveal no bridging feature to the Zr atoms (threshold > 1.3%)  65. (2.00000) 97.7420% BD ( 1) C 7- N 68  40.528% C 7 s( 27.66%)p 2.61( 72.19%)d 0.00( 0.12%) f 0.00( 0.03%)  57.378% N 68 s( 44.46%)p 1.24( 55.10%)d 0.01( 0.43%) f 0.00( 0.01%) | Shortened NLMO 65 Analysis of C(7)-N(68) π-bond reveal only small binding contributions from the Zr(71) atom (threshold > 1.3%)  66. (2.00000) 92.4285% BD ( 2) C 7- N 68  37.556% C 7 s( 0.00%)p 1.00( 99.78%)d 0.00( 0.17%) f 0.00( 0.04%)  2.967% C 8 s( 0.00%)p 1.00( 99.54%)d 0.00( 0.41%) f 0.00( 0.06%)  54.901% N 68 s( 0.01%)p 1.00( 99.75%)d 0.00( 0.23%) f 0.00( 0.02%)  2.618% Zr 71 s( 0.00%)p 1.00( 0.58%)d99.99( 98.62%)f 1.35( 0.79%) |
| 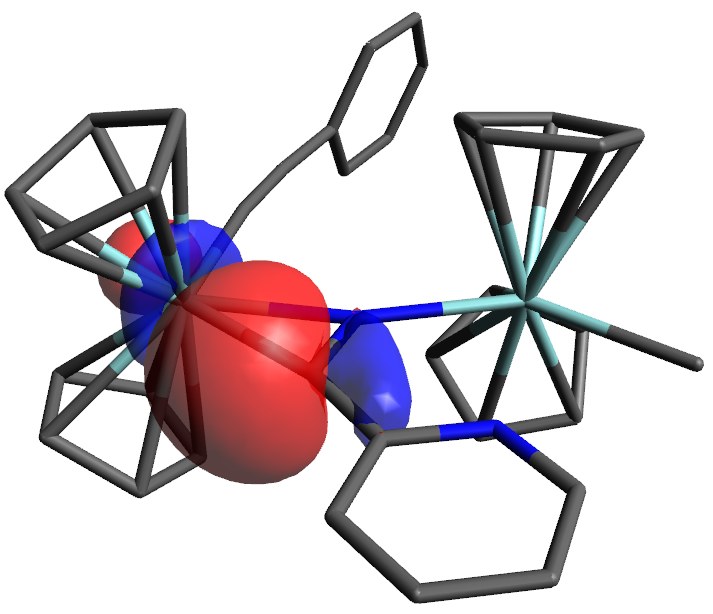 |  |
| Shortened NLMO 62 Analysis of C(7)-Zr(71) σ-bond (threshold > 1.3%)  67. (2.00000) 94.8021% BD ( 1) C 7-Zr 71  73.392% C 7 s( 32.50%)p 2.07( 67.43%)d 0.00( 0.05%) f 0.00( 0.01%)  1.347% Zr 70 s( 16.28%)p 0.06( 1.04%)d 5.08( 82.65%) f 0.00( 0.04%)  21.615% Zr 71 s( 6.64%)p 0.01( 0.08%)d14.03( 93.18%) f 0.01( 0.09%) |  |

#### QT-AIM analysis of (Cp_2_Zr)_2_(CN-Py)Ph (**8**)


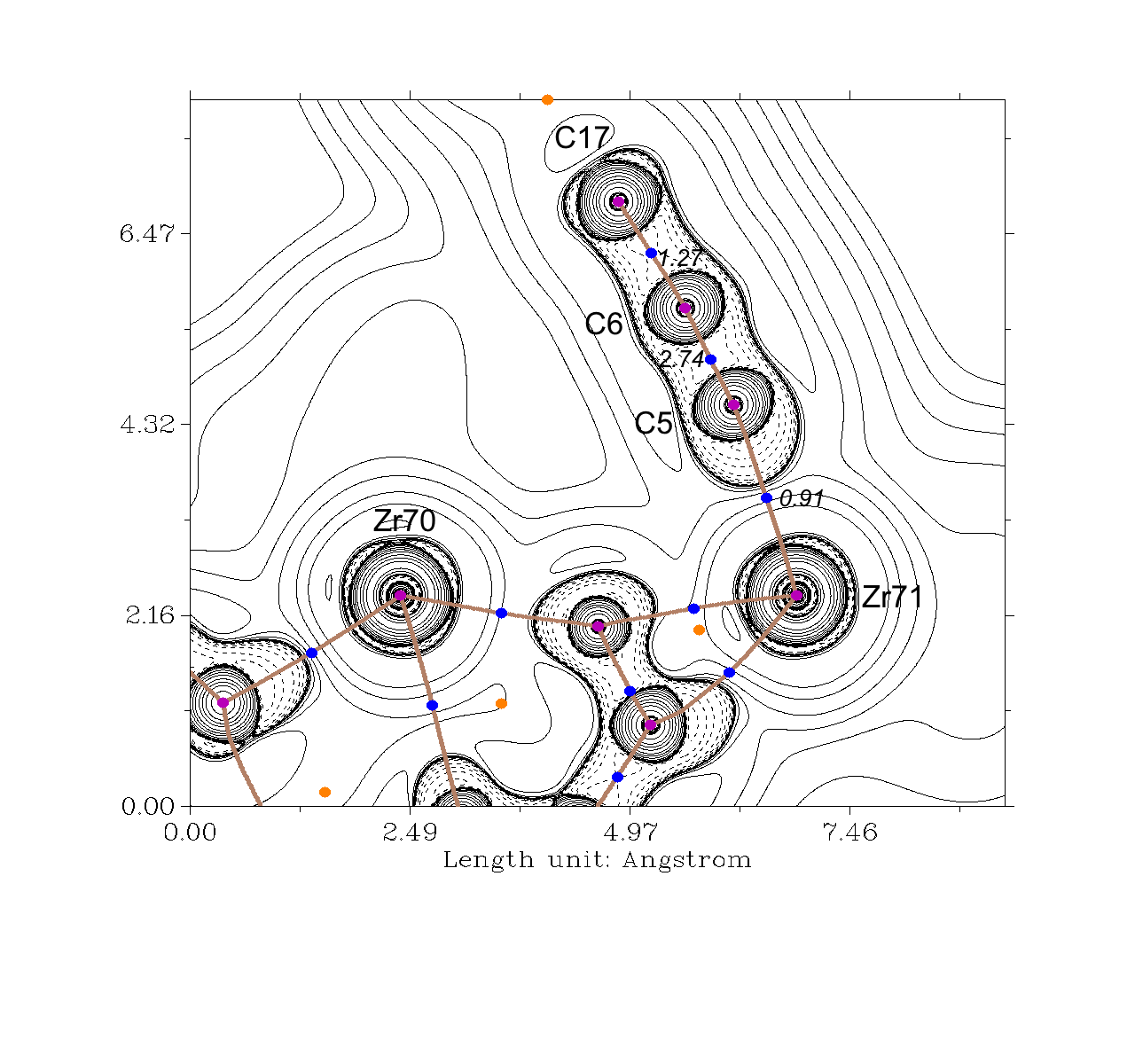


**Figure S21.** Contour plot of the Laplacian of the electron density ∇^2^*r* of complex **8** in the Zr70-Zr71-C6 plane. Dashed lines indicate negative (local charge concentration), solid lines indicate positive values (local charge depletion). The Laplacian plot is overlaid with the molecular graph from QT-AIM analysis and Wiberg bond indices (italic small numbers). Brown lines indicate bond paths, brown dashed lines are hypothetical bonds, blue dots correspond to bond critical points, light brown dots indicate ring critical points. Density from B3LYP-D3/def2-TZVP calculation.


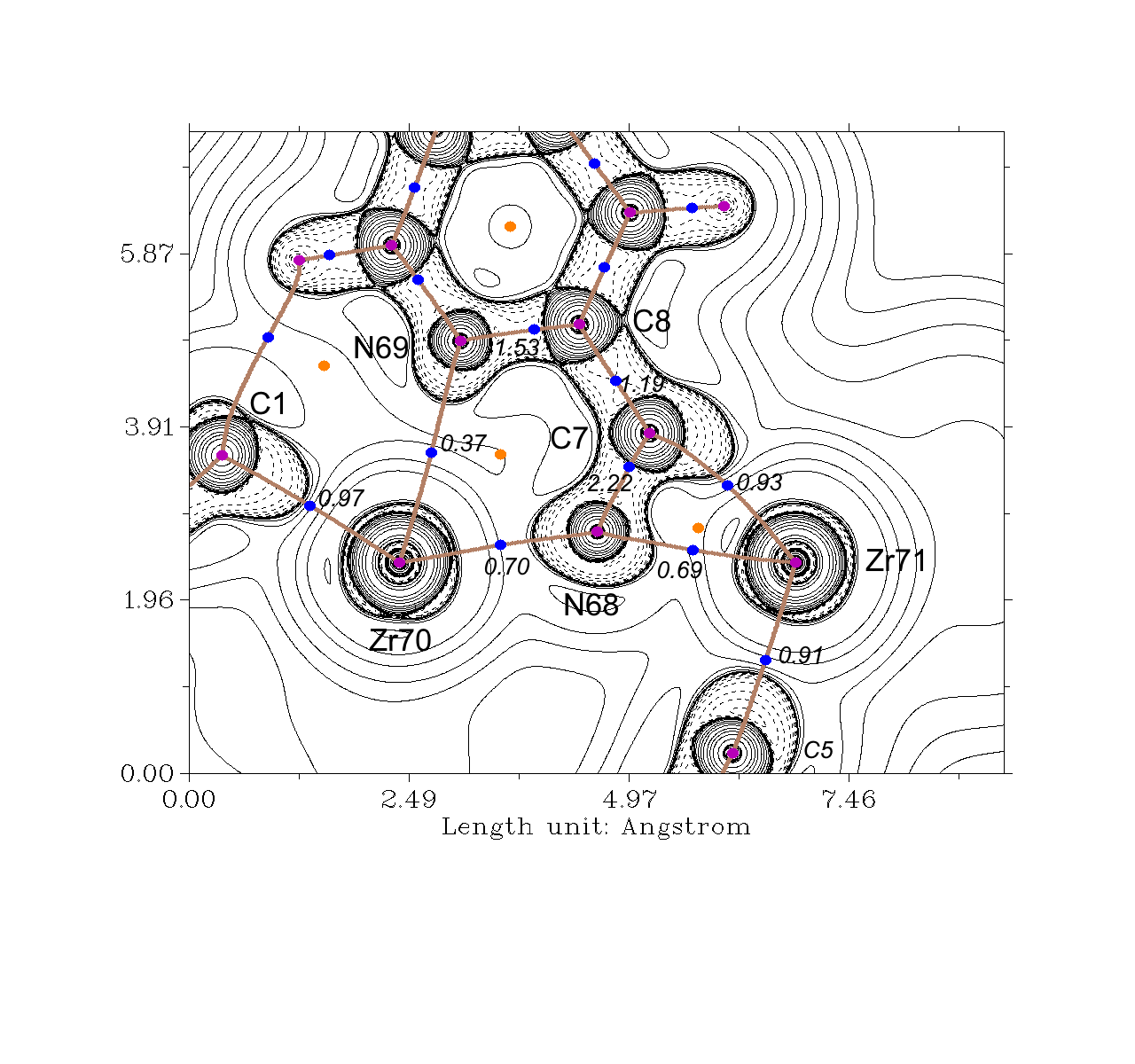


**Figure S22.** Contour plot of the Laplacian of the electron density ∇^2^*r* of complex **8** in the Zr70-Zr71-N69 plane. Dashed lines indicate negative (local charge concentration), solid lines indicate positive values (local charge depletion). The Laplacian plot is overlaid with the molecular graph from QT-AIM analysis and Wiberg bond indices (italic small numbers). Brown lines indicate bond paths, brown dashed lines are hypothetical bonds, blue dots correspond to bond critical points, light brown dots indicate ring critical points. Density from B3LYP-D3/def2-TZVP calculation.

### **Bond analysis of (Cp_2_Zr)_2_(NCCH_3_)(H_3_CC_2_Ph) (9)**


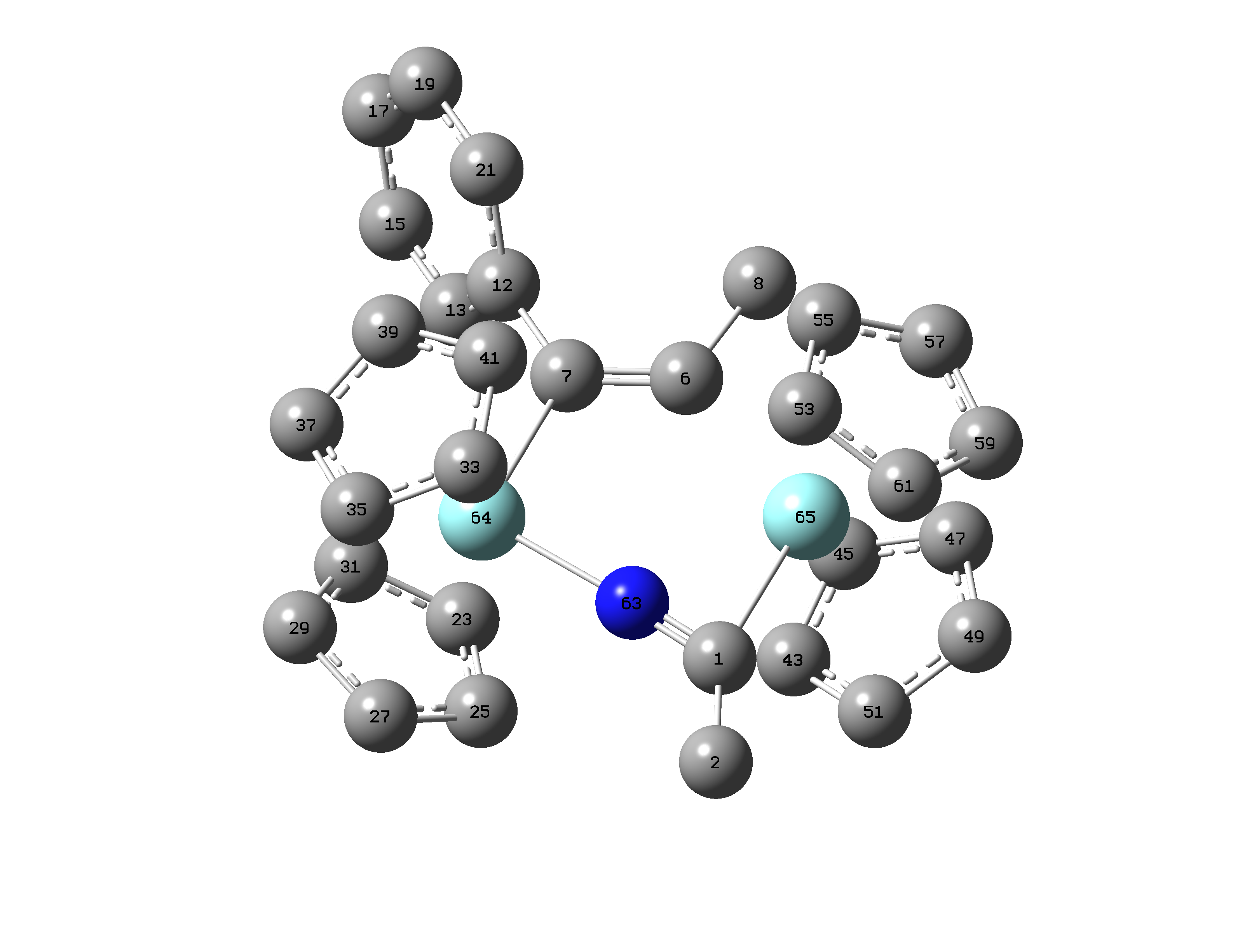


**Figure S23.** Labelling scheme for the bond analysis of complex **9**. Hydrogen atoms are omitted for clarity.

#### NBO/NLMO analysis of (Cp_2_Zr)_2_(NCCH_3_)(H_3_CC_2_Ph) (**9**)

For this purpose, the molecule was divided into four logical units and the natural charges of the atoms of these were summed up.

**Table S12.** Summary of selected NBO analysis of **9**.

| 46. (1.75589) LP ( 1) N 63  s( 40.92%)p 1.44( 59.06%)d 0.00( 0.00%) f 0.00( 0.01%) | 47. (1.57781) LP ( 2) N 63  s( 1.73%)p56.67( 98.14%)d 0.06( 0.11%) f 0.01( 0.02%) |
| --- | --- |
| 49. (1.95419) BD ( 1) C 1- N 63  ( 39.63%) 0.6295* C 1 s( 31.56%)p 2.16( 68.31%)d 0.00( 0.10%) f 0.00( 0.03%)  ( 60.37%) 0.7770* N 63 s( 57.06%)p 0.75( 42.56%)d 0.01( 0.36%) f 0.00( 0.02%) | 50. (1.90879) BD ( 2) C 1- N 63  ( 33.38%) 0.5778* C 1 s( 0.00%)p 1.00( 99.78%)d 0.00( 0.18%) f 0.00( 0.03%)  ( 66.62%) 0.8162* N 63 s( 0.01%)p 1.00( 99.82%)d 0.00( 0.13%) f 0.00( 0.03%) |
| 51. (1.86257) BD ( 1) C 1-Zr 65  ( 77.29%) 0.8791* C 1 s( 30.41%)p 2.29( 69.51%)d 0.00( 0.08%) f 0.00( 0.01%)  ( 22.71%) 0.4766*Zr 65 s( 5.65%)p 0.01( 0.05%)d16.69( 94.22%) f 0.02( 0.09%) | 55. (1.95591) BD ( 1) C 6- C 7  ( 48.79%) 0.6985* C 6 s( 38.67%)p 1.58( 61.13%)d 0.00( 0.17%) f 0.00( 0.04%)  ( 51.21%) 0.7156* C 7 s( 40.63%)p 1.46( 59.17%)d 0.00( 0.15%) f 0.00( 0.05%) |
| 56. (1.85571) BD ( 2) C 6- C 7  ( 47.44%) 0.6888* C 6 s( 0.02%)p99.99( 99.90%)d 2.36( 0.06%)f 1.12( 0.03%)  ( 52.56%) 0.7250* C 7 s( 0.08%)p99.99( 99.84%)d 0.58( 0.04%) f 0.51( 0.04%) | 58. (1.86146) BD ( 1) C 6-Zr 65  ( 79.04%) 0.8891* C 6 s( 32.11%)p 2.11( 67.85%)d 0.00( 0.03%) f 0.00( 0.01%)  ( 20.96%) 0.4578*Zr 65 s( 10.80%)p 0.01( 0.09%)d 8.25( 89.06%) f 0.01( 0.06%) |
| 60. (1.86744) BD ( 1) C 7-Zr 64  ( 78.09%) 0.8837* C 7 s( 27.88%)p 2.58( 72.04%)d 0.00( 0.06%) f 0.00( 0.01%)  ( 21.91%) 0.4681*Zr 64 s( 12.38%)p 0.01( 0.11%)d 7.07( 87.46%)f 0.00( 0.05%) |  |

**Table S13.** Summary of selected NLMOs of **9** (iso0.04).

| 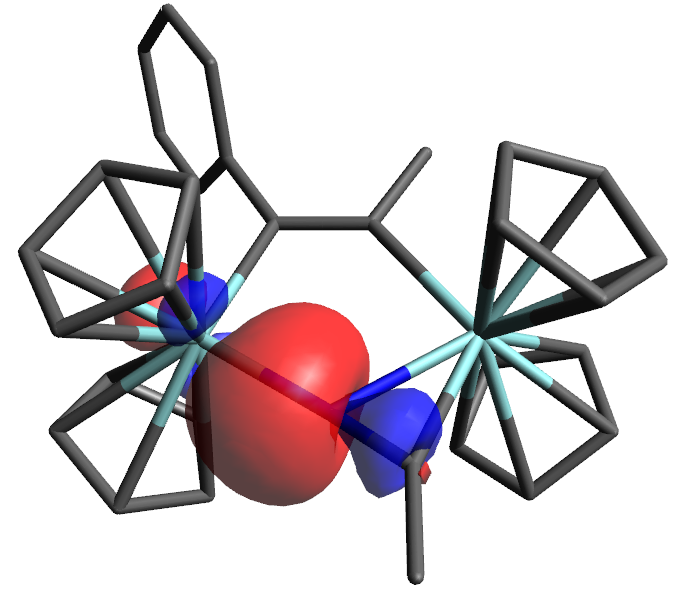 | 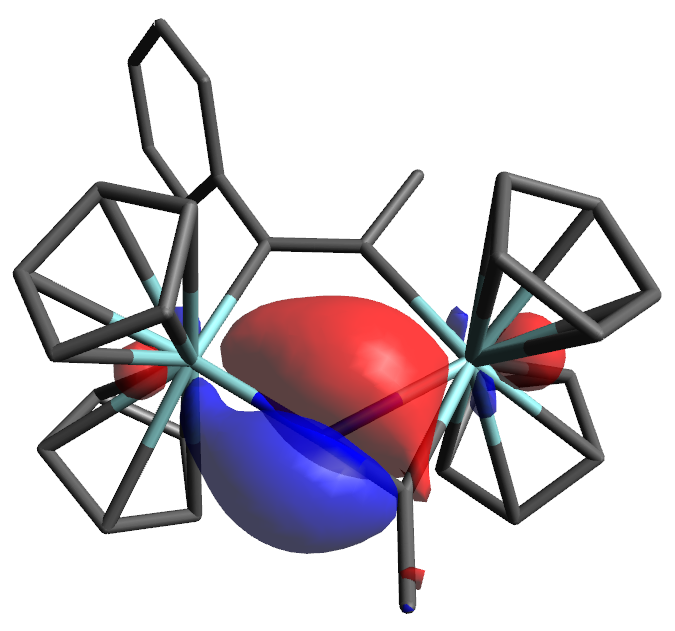 |
| --- | --- |
| Shortened NLMO 46 Analysis of LP (1) N(63) indicates a strongly polarised binding feature to Zr(63) next to small contributions by Zr(65) atom (threshold > 1.3%)  46. (2.00000) 86.9644% LP ( 1) N 63  87.084% N 63 s( 42.08%)p 1.38( 57.90%)d 0.00( 0.00%) f 0.00( 0.01%)  10.335% Zr 64 s( 14.83%)p 0.02( 0.23%)d 5.72( 84.79%) f 0.01( 0.15%)  1.356% Zr 65 s( 31.51%)p 0.02( 0.51%)d 2.15( 67.81%) f 0.01( 0.18%) | Shortened NLMO 53 Analysis of LP (2) N(63) indicates a strongly polarised binding/bridging feature to both Zr(64, 65) atoms next to small contribution by C(1) atom (threshold > 1.3%)  47. (2.00000) 77.3391% LP ( 2) N 63  2.520% C 1 s( 1.57%)p61.77( 96.79%)d 0.97( 1.51%) f 0.09( 0.14%)  77.515% N 63 s( 3.15%)p30.70( 96.73%)d 0.03( 0.10%) f 0.01( 0.02%)  7.513% Zr 64 s( 2.05%)p 0.07( 0.15%)d47.46( 97.51%) f 0.14( 0.29%)  9.085% Zr 65 s( 5.54%)p 0.01( 0.05%)d17.01( 94.28%) f 0.02( 0.12%) |
| 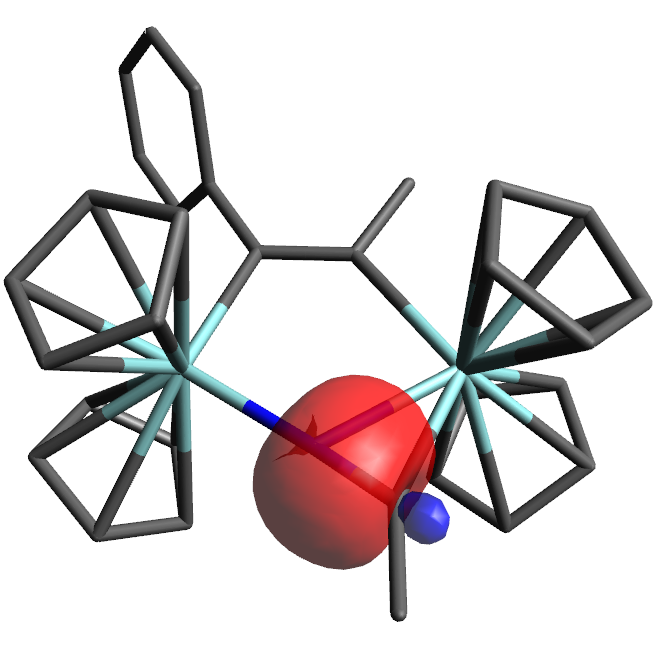 | 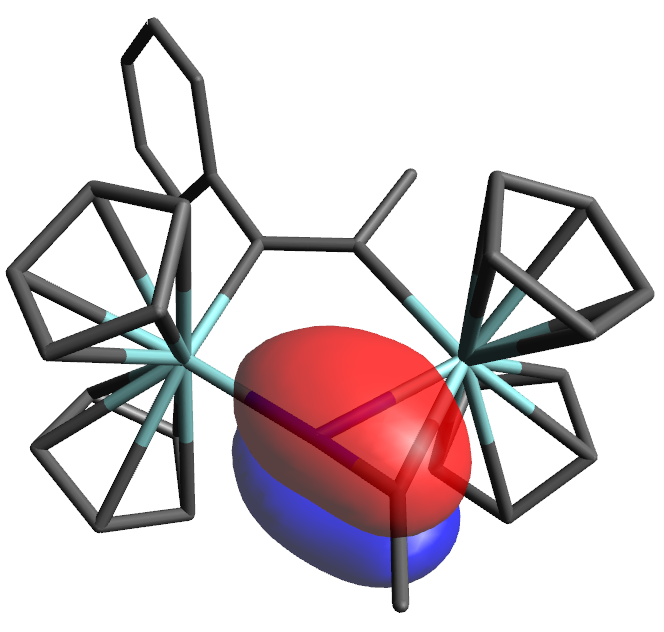 |
| Shortened NLMO 49 Analysis of C(1)-N(63) σ-bond (threshold > 1.3%)  49. (2.00000) 97.6498% BD ( 1) C 1- N 63  38.743% C 1 s( 29.01%)p 2.44( 70.86%)d 0.00( 0.10%) f 0.00( 0.03%)  59.096% N 63 s( 53.48%)p 0.86( 46.14%)d 0.01( 0.36%) f 0.00( 0.02%) | Shortened NLMO 50 Analysis of C(1)-N(63) π-bond reveals only small binding contributions by both Zr(64, 65) atoms (threshold > 1.3%)  50. (2.00000) 95.2435% BD ( 2) C 1- N 63  31.728% C 1 s( 0.00%)p 1.00( 99.78%)d 0.00( 0.19%) f 0.00( 0.03%)  63.519% N 63 s( 0.00%)p 1.00( 99.83%)d 0.00( 0.13%) f 0.00( 0.03%)  1.339% Zr 64 s( 0.00%)p 1.00( 1.23%)d79.51( 97.55%) f 0.99( 1.22%)  2.218% Zr 65 s( 0.06%)p 6.81( 0.41%)d99.99( 98.66%) f14.29( 0.87%) |
| 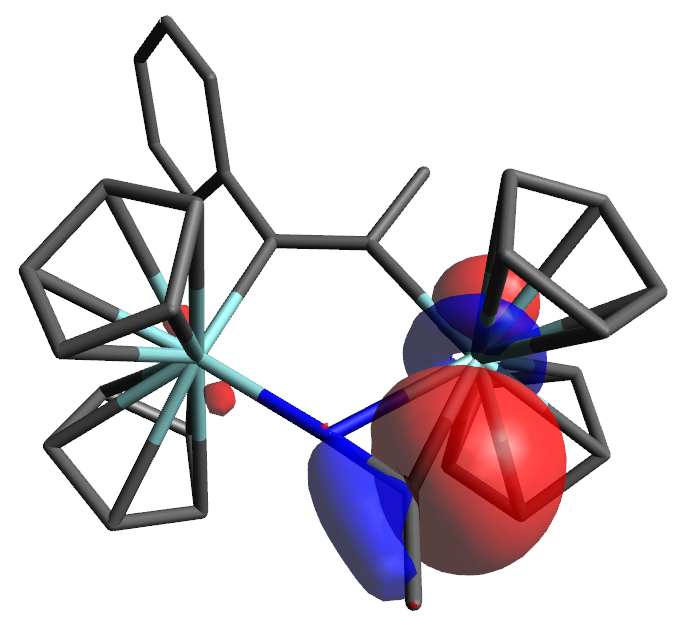 | 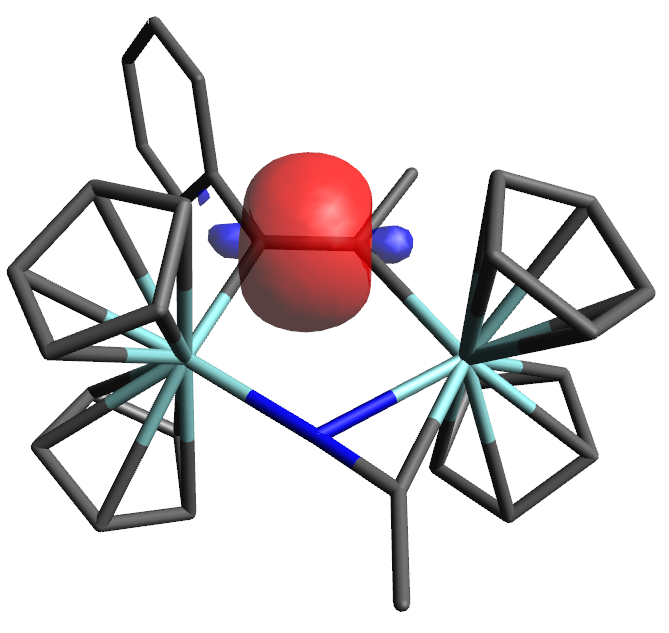 |
| Shortened NLMO 51 Analysis of C(1)-Zr(65) σ-bond reveal small contributions of the Zr(64) atom (threshold > 1.3%)  51. (2.00000) 92.8948% BD ( 1) C 1-Zr 65  71.426% C 1 s( 30.47%)p 2.28( 69.44%)d 0.00( 0.08%) f 0.00( 0.01%)  2.861% Zr 64 s( 2.96%)p 0.24( 0.70%)d32.56( 96.32%) f 0.01( 0.03%)  21.629% Zr 65 s( 6.51%)p 0.01( 0.05%)d14.34( 93.35%) f 0.01( 0.09%) | Shortened NLMO 55 Analysis of C(6)-C(7) σ -bond (threshold > 1.3%)  55. (2.00000) 97.7756% BD ( 1) C 6- C 7  47.768% C 6 s( 34.92%)p 1.86( 64.88%)d 0.00( 0.17%) f 0.00( 0.04%)  50.164% C 7 s( 37.03%)p 1.70( 62.77%)d 0.00( 0.15%)f 0.00( 0.05%) |
| 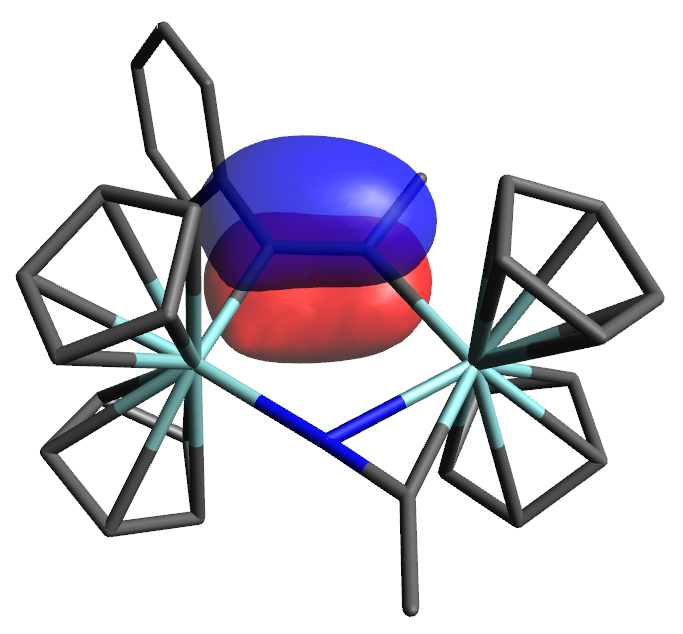 | 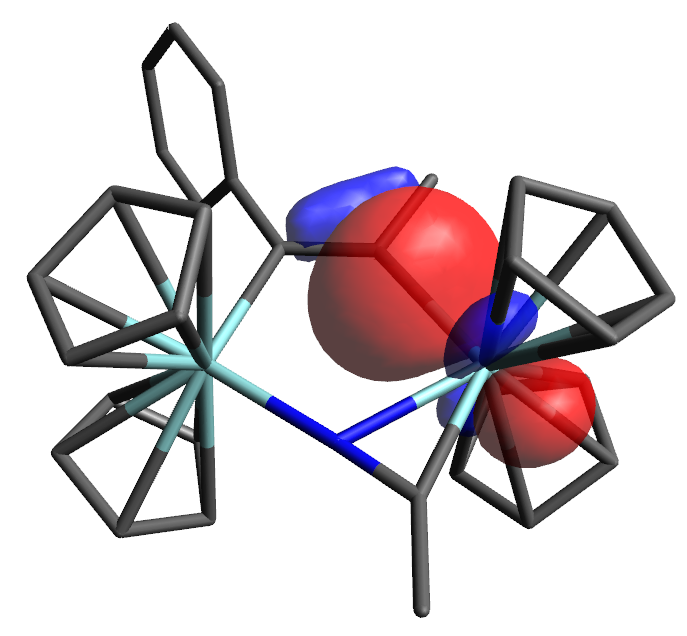 |
| Shortened NLMO 56 Analysis of C(6)-C(7) π-bond reveal only small contributions of both Zr(64, 65) atoms (threshold > 1.3%)  56. (2.00000) 92.5796% BD ( 2) C 6- C 7  43.890% C 6 s( 0.02%)p99.99( 99.90%)d 3.36( 0.05%) f 1.61( 0.03%)  48.692% C 7 s( 0.08%)p99.99( 99.83%)d 0.54( 0.04%) f 0.47( 0.04%)  2.876% Zr 64 s( 0.03%)p12.14( 0.34%)d99.99( 99.05%) f21.17( 0.59%)  1.382% Zr 65 s( 0.01%)p59.26( 0.69%)d99.99( 98.65%) f54.86( 0.64%) | Shortened NLMO 62 Analysis of C(6)-Zr(65) σ-bond reveal only small contributions of the Zr(64) atom (threshold > 1.3%)  58. (2.00000) 92.9333% BD ( 1) C 6-Zr 65  73.664% C 6 s( 30.55%)p 2.27( 69.41%)d 0.00( 0.03%) f 0.00( 0.01%)  2.344% Zr 64 s( 6.17%)p 0.03( 0.16%)d15.17( 93.59%) f 0.01( 0.08%)  19.455% Zr 65 s( 12.49%)p 0.01( 0.08%)d 6.99( 87.37%) f 0.00( 0.05%) |
| 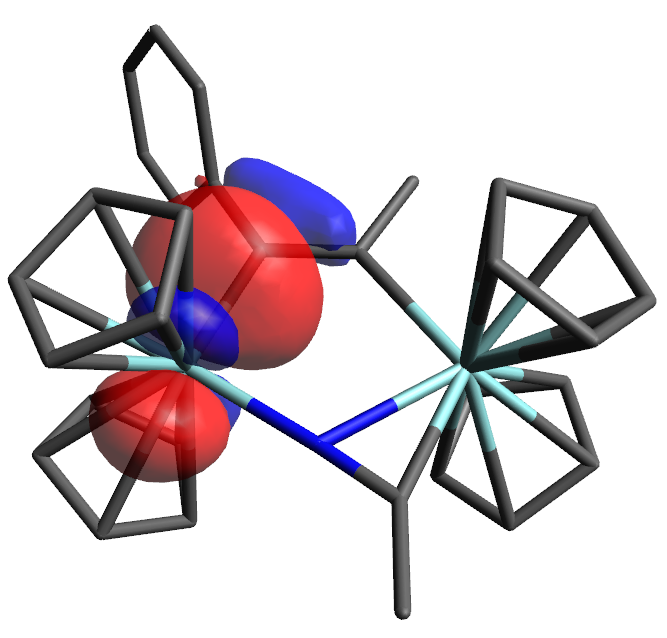 |  |
| Shortened NLMO 60 Analysis of C(7)-Zr(64) σ-bond (threshold > 1.3%)  60. (2.00000) 93.3209% BD ( 1) C 7-Zr 64  73.107% C 7 s( 26.09%)p 2.83( 73.84%)d 0.00( 0.06%) f 0.00( 0.01%)  1.620% C 12 s( 3.13%)p30.64( 95.99%)d 0.27( 0.85%) f 0.01( 0.03%)  20.262% Zr 64 s( 12.50%)p 0.01( 0.11%)d 6.99( 87.33%) f 0.00( 0.05%) |  |

#### QT-AIM analysis of (Cp_2_Zr)_2_(NCCH_3_)(H_3_CC_2_Ph) (**9**)


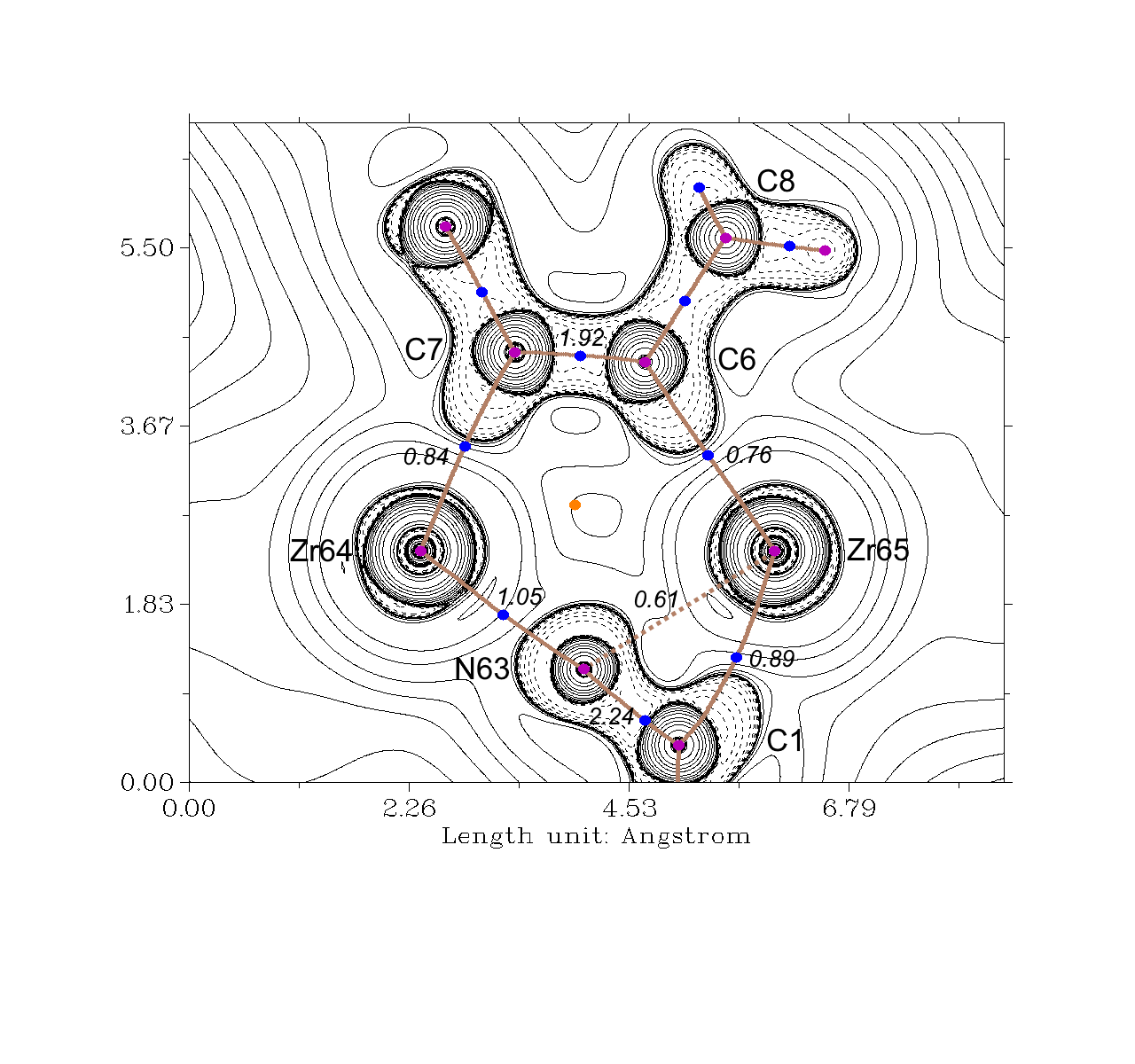


**Figure S24.** Contour plot of the Laplacian of the electron density ∇^2^*r* of complex **9** in the Zr64-Zr75-C6 plane. Dashed lines indicate negative (local charge concentration), solid lines indicate positive values (local charge depletion). The Laplacian plot is overlaid with the molecular graph from QT-AIM analysis and Wiberg bond indices (italic small numbers). Brown lines indicate bond paths, brown dashed lines are hypothetical bonds, blue dots correspond to bond critical points, light brown dots indicate ring critical points. Density from B3LYP-D3/def2-TZVP calculation.

## Bond analysis of small molecules for comparison

The following bond analysis were performed on the same level of theory as mentioned above for the Zr complexes. The results are shown here for comparison, supporting the discussion of the data of the Zr complexes.

### QT-AIM analysis of phenylacetylene


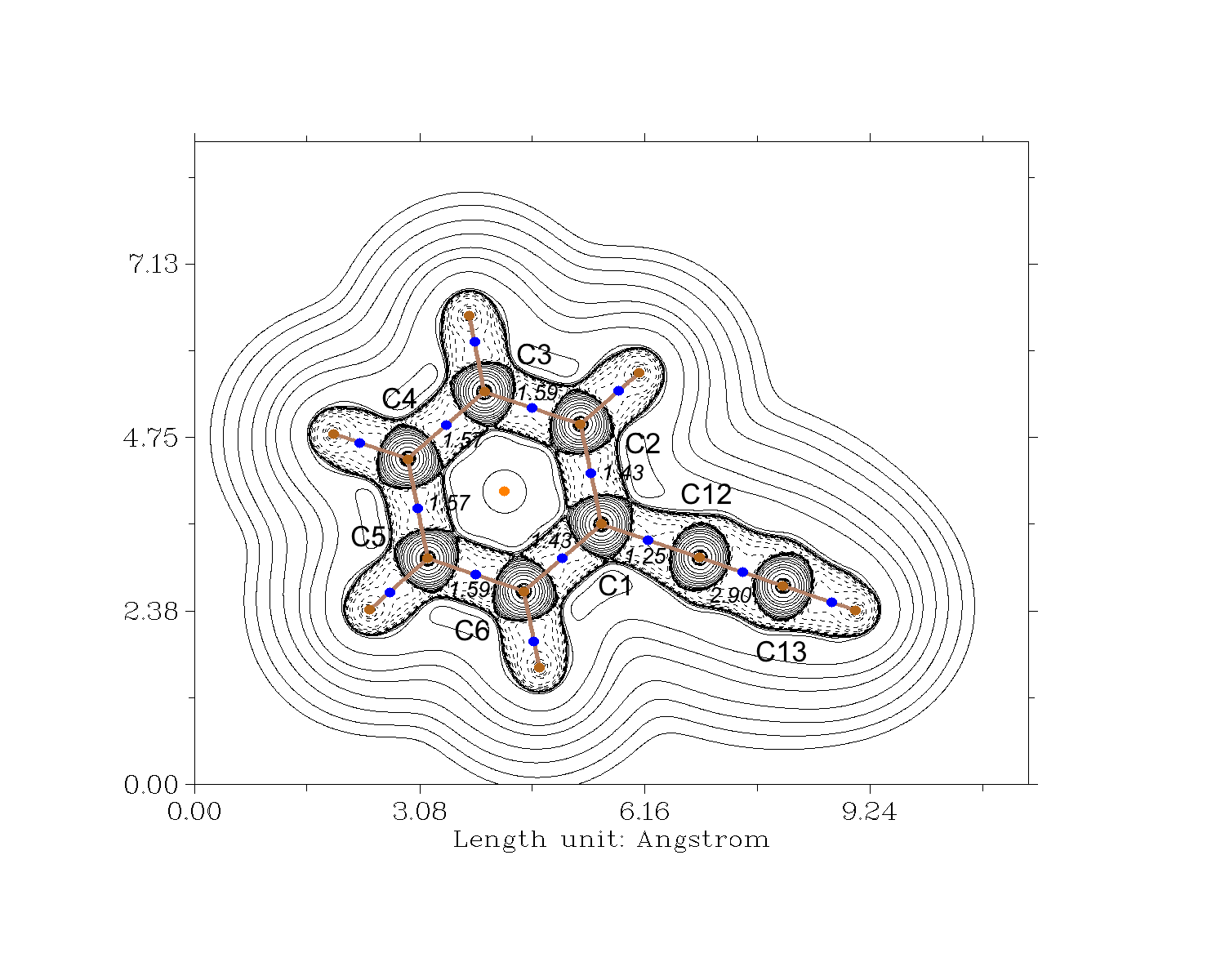


**Figure S25.** Contour plot of the Laplacian of the electron density ∇^2^*r* of starting material phenylacethylene in the C3-C6-C13 plane. Dashed lines indicate negative (local charge concentration), solid lines indicate positive values (local charge depletion). The Laplacian plot is overlaid with the molecular graph from QT-AIM analysis and Wiberg bond indices (italic small numbers). Brown lines indicate bond paths, blue dots correspond to bond critical points, light brown dots indicate ring critical points. Density from B3LYP-D3/def2-TZVP calculation.

### QT-AIM analysis of 2-cyanopyridine


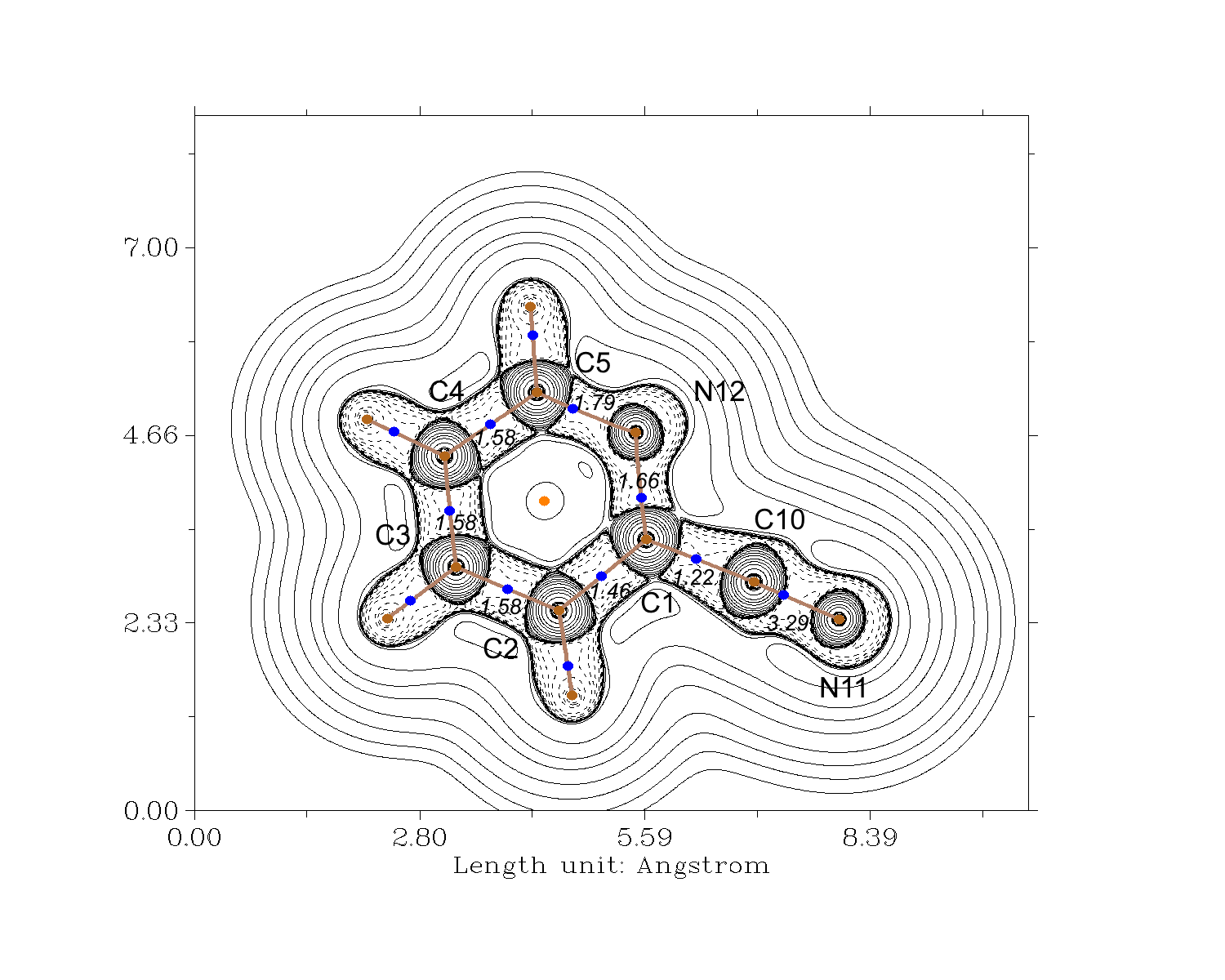


**Figure S26.** Contour plot of the Laplacian of the electron density ∇^2^*r* of starting material 2-cyanopyridine in the C3-C6-C13 plane. Dashed lines indicate negative (local charge concentration), solid lines indicate positive values (local charge depletion). The Laplacian plot is overlaid with the molecular graph from QT-AIM analysis and Wiberg bond indices (italic small numbers). Brown lines indicate bond paths, blue dots correspond to bond critical points, light brown dots indicate ring critical points. Density from B3LYP-D3/def2-TZVP calculation.

### QT-AIM analysis of acetonitrile


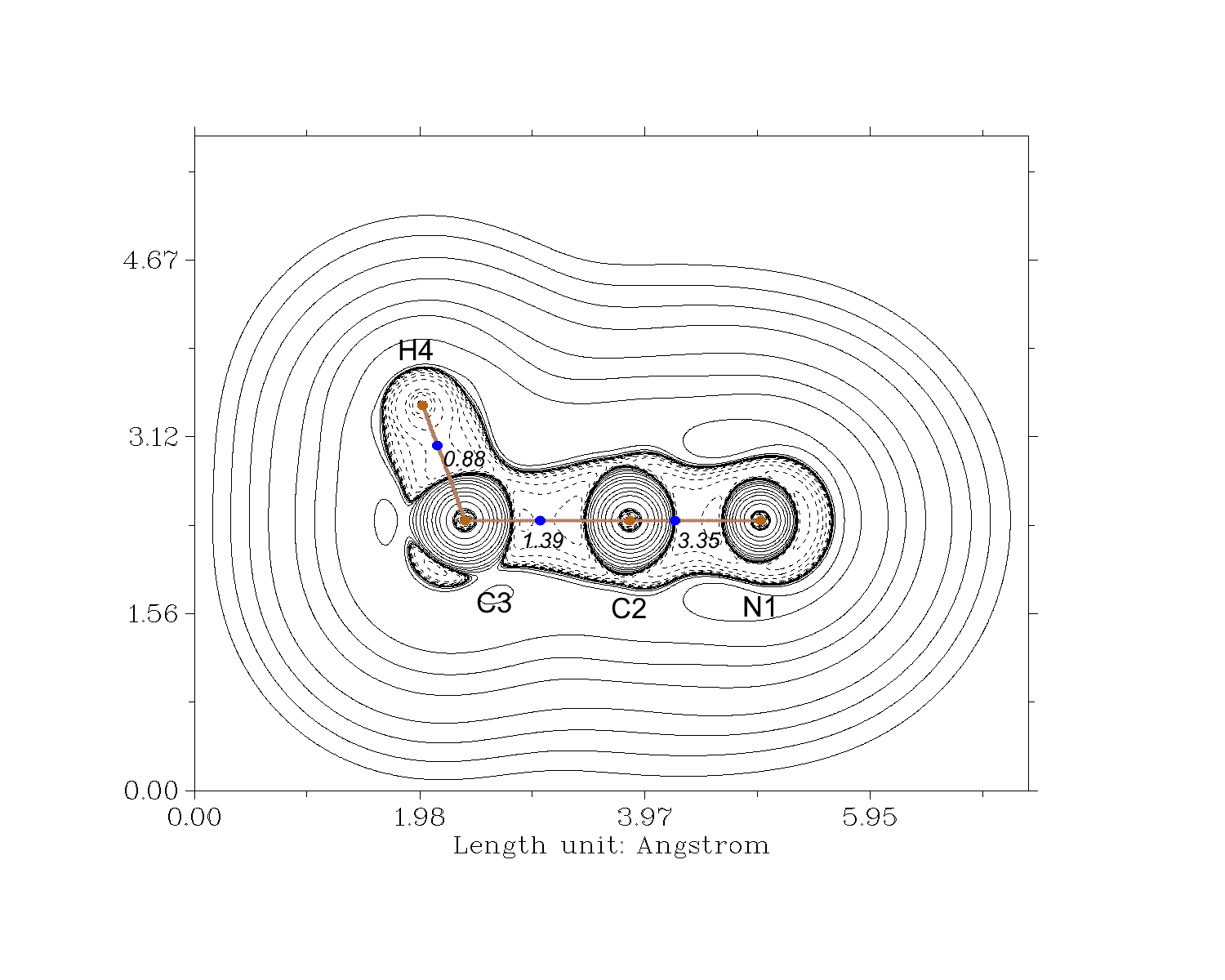


**Figure S27.** Contour plot of the Laplacian of the electron density ∇^2^*r* of starting material acetonitrile in the H4-C2-N1 plane. Dashed lines indicate negative (local charge concentration), solid lines indicate positive values (local charge depletion). The Laplacian plot is overlaid with the molecular graph from QT-AIM analysis and Wiberg bond indices (italic small numbers). Brown lines indicate bond paths, blue dots correspond to bond critical points, light brown dots indicate ring critical points. Density from B3LYP-D3/def2-TZVP calculation.

### QT-AIM Analysis of diborane


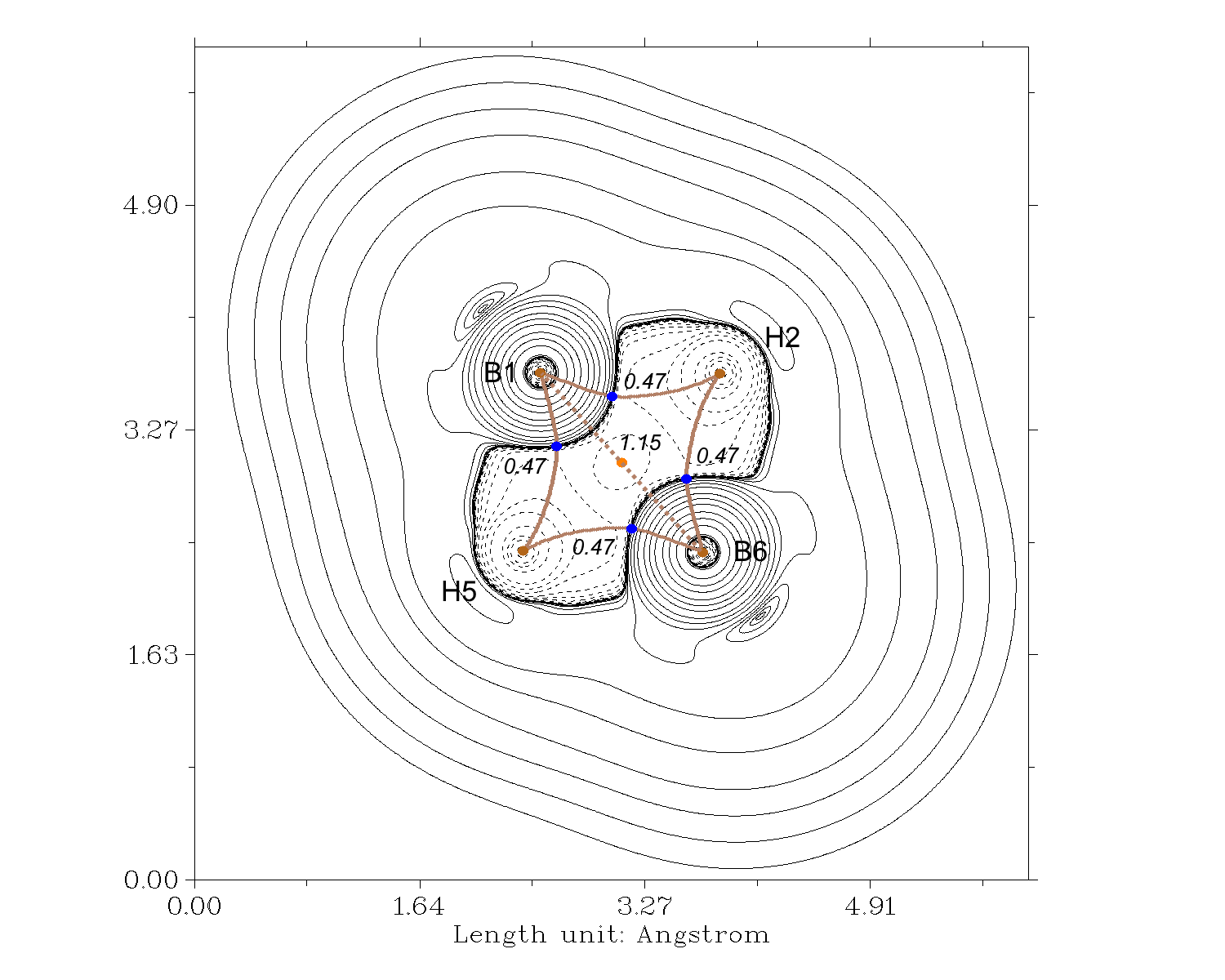


**Figure S28.** Contour plot of the Laplacian of the electron density ∇^2^*r* of diborane (B_2_H_6_) in the multicentre bonding plane B1-H2-B6. Dashed lines indicate negative (local charge concentration), solid lines indicate positive values (local charge depletion). The Laplacian plot is overlaid with the molecular graph from QT-AIM analysis and Wiberg bond indices (italic small numbers). Brown lines indicate bond paths, blue dots correspond to bond critical points, light brown dots indicate ring critical points. Density from B3LYP-D3/def2-TZVP calculation, optimised in *D*2*h* symmetry.

## Investigation of charge transfer based on TD-DFT

### **TD-DFT analysis of (Cp_2_Zr)_2_(µ-Me)(µ-C_2_Ph) (5)**

** Figure S29.** Plot of the theoretical (TD-DFT) UV-vis spectrum of **5** suggesting a half-width at half-height of 0.25 eV. This reveals the second excited state as responsible for the orange colour of the complex.

Excitation energies and oscillator strengths:

Excited State 1: Singlet-A 2.4811 eV 499.71 nm f=0.0008 <S**2>=0.000

113 -> 114 0.69916

This state for optimization and/or second-order correction.

Total Energy, E(TD-HF/TD-DFT) = -1215.21347529

Copying the excited state density for this state as the 1-particle RhoCI density.

Excited State 2: Singlet-A 2.8662 eV 432.57 nm f=0.0387 <S**2>=0.000

113 -> 115 0.64674

113 -> 116 0.18234

113 -> 117 -0.17247

Excited State 3: Singlet-A 3.5852 eV 345.83 nm f=0.0021 <S**2>=0.000

113 -> 116 -0.32440

113 -> 117 -0.14443

113 -> 118 0.57951

**Table S14.** Representation of the molecular orbitals which are involved in the second excited state of complex **5** (PBE0-D3/def2-TZVP).

| HOMO 113 (iso0.04, -4.817 eV) | LUMO+1 115 (iso0.04, -0.815 eV) |
| --- | --- |
| 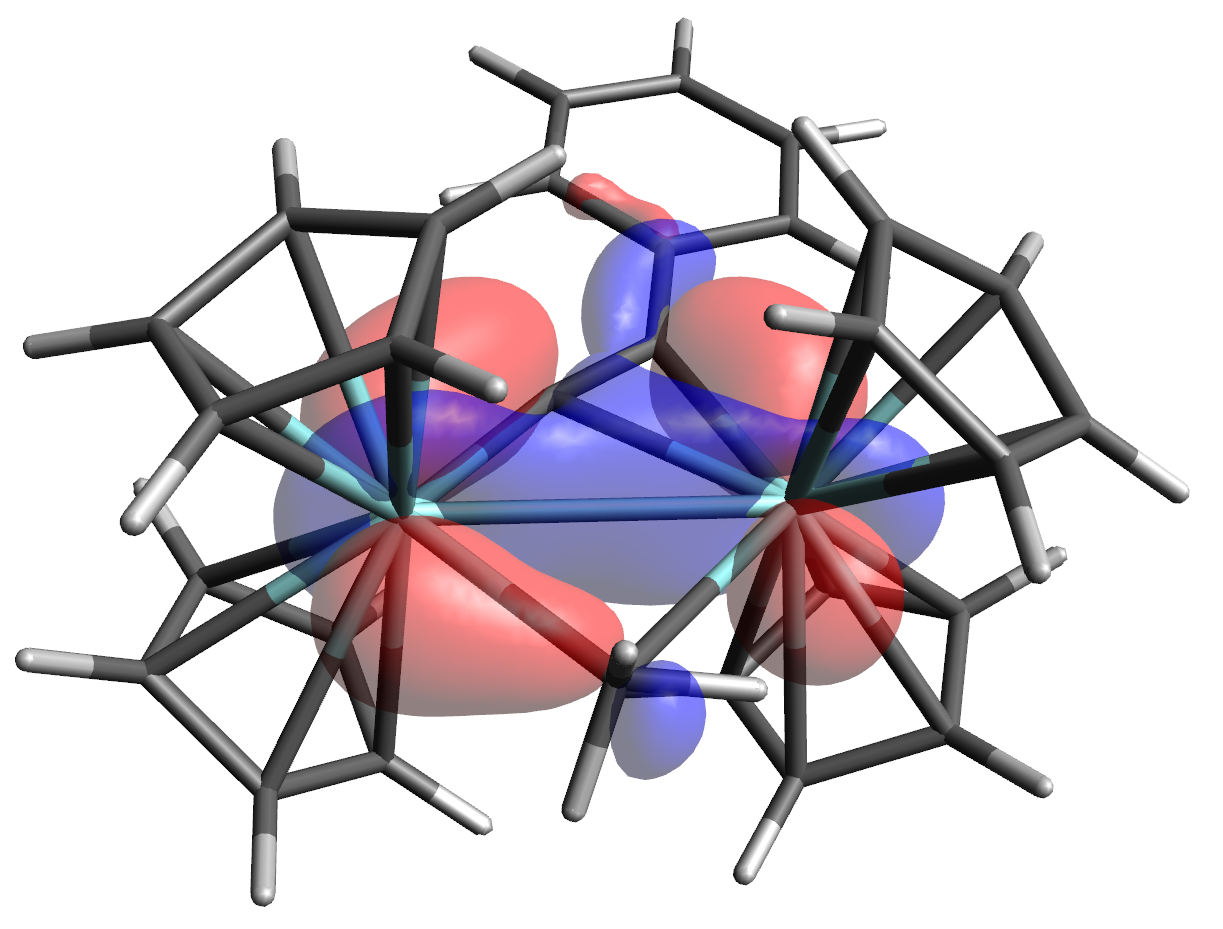 | 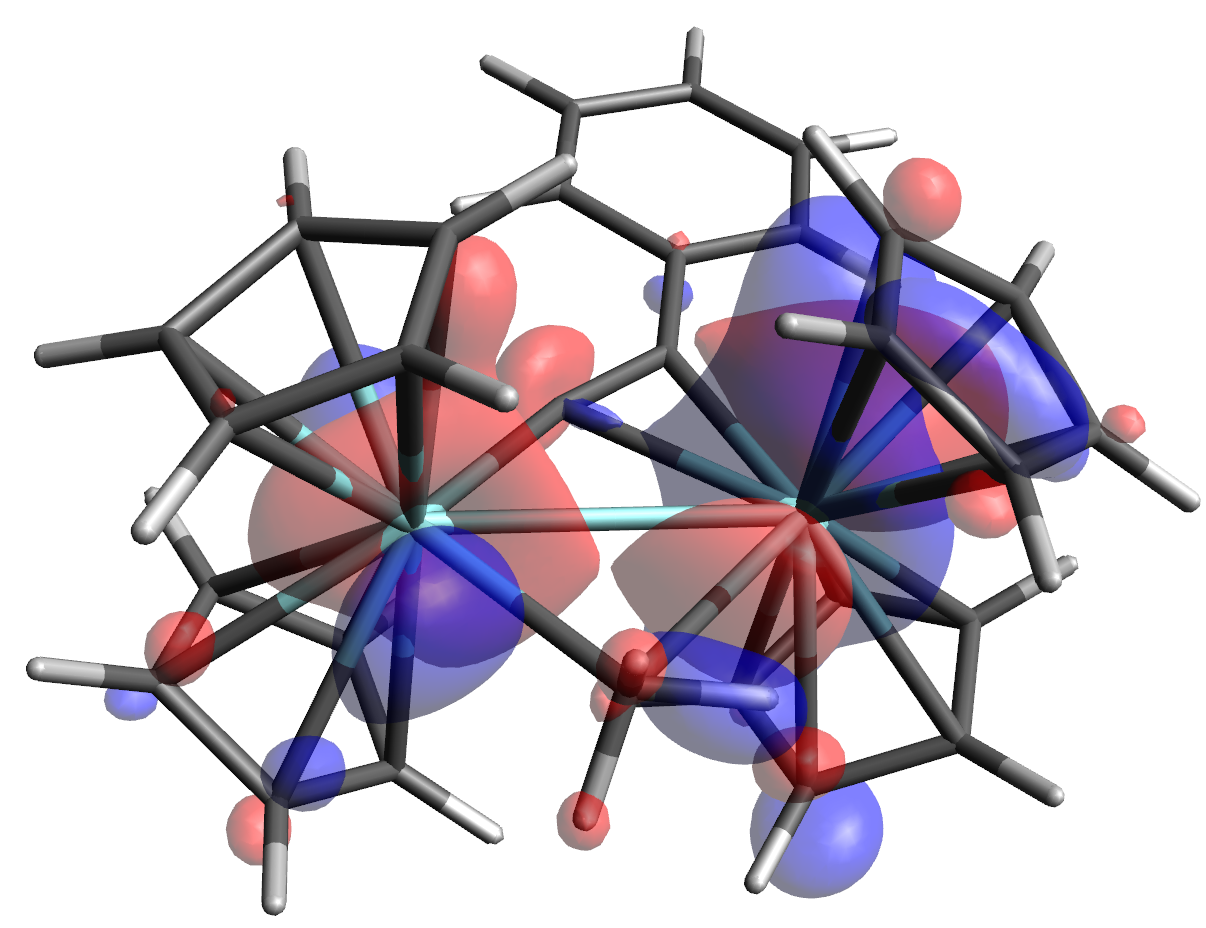 |
| LUMO+2 116 (iso0.04, -0.140 eV) | LUMO+3 117 (iso0.04, -0.100 eV) |
| 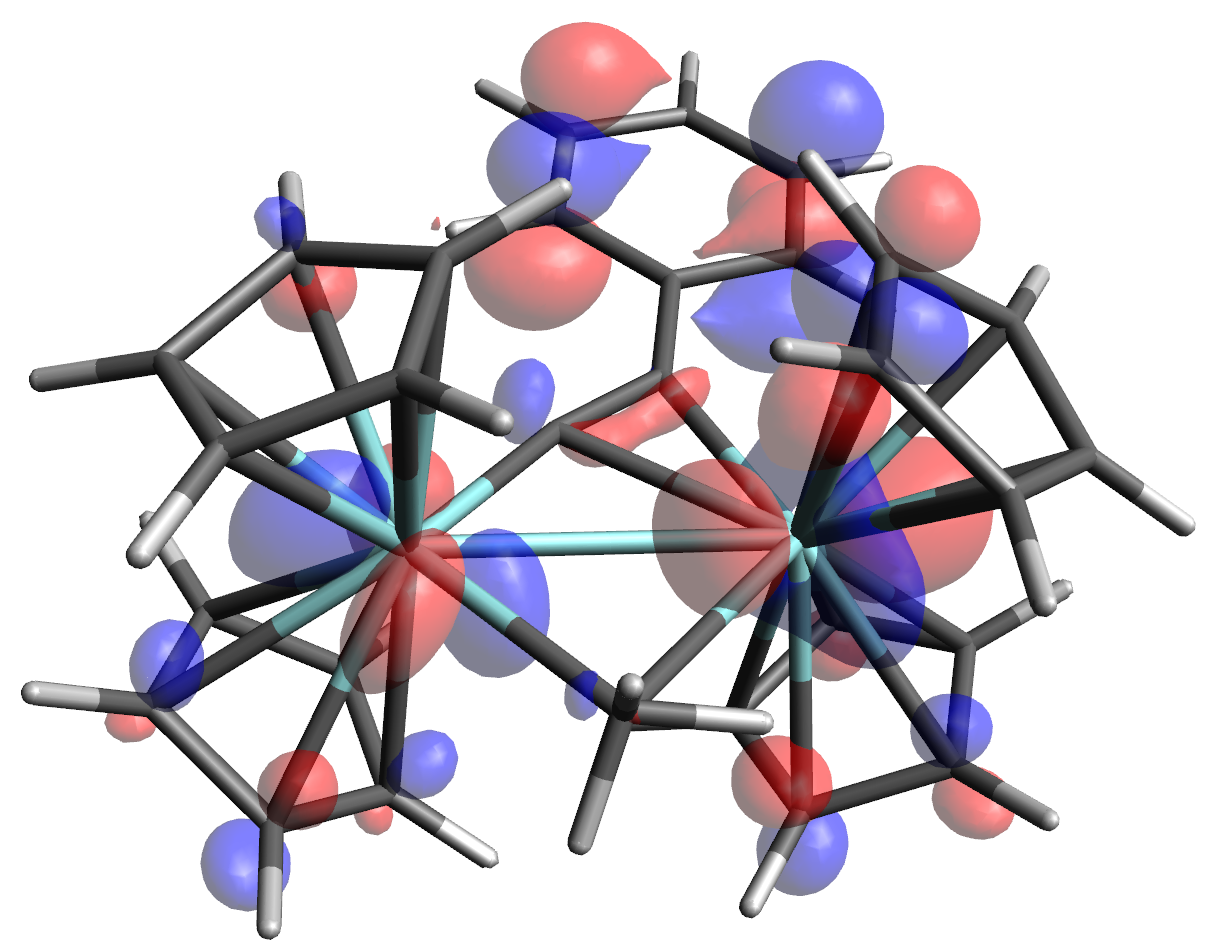 | 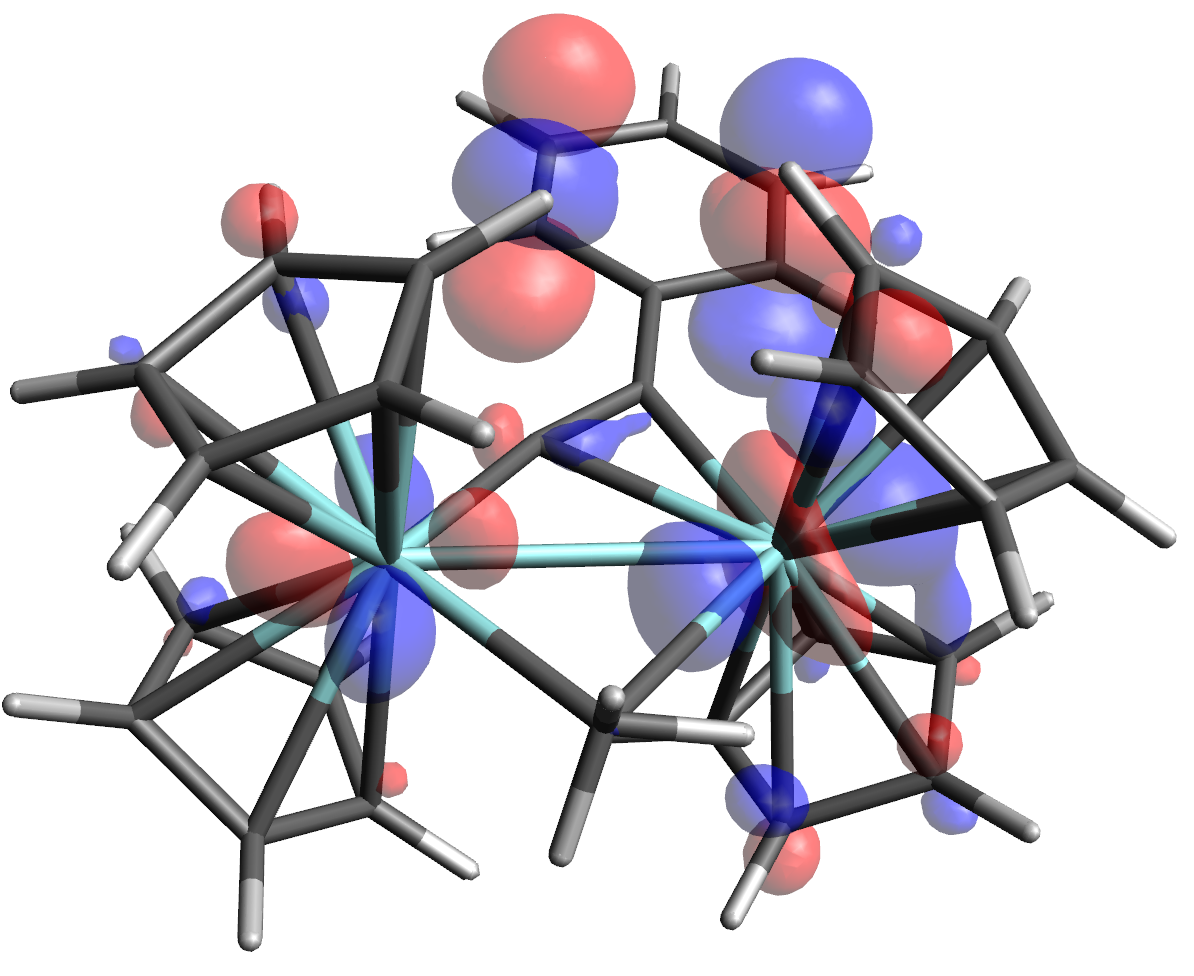 |

**Table S15.** Analysis of the charge density differences (CCD) of the second excited state of **5** (green regions are electron accepting and blue regions electron donating due to the excitation (PBE0-D3/def2-TZVP)

| CDD EX2 (iso0.004) |  |
| --- | --- |
| 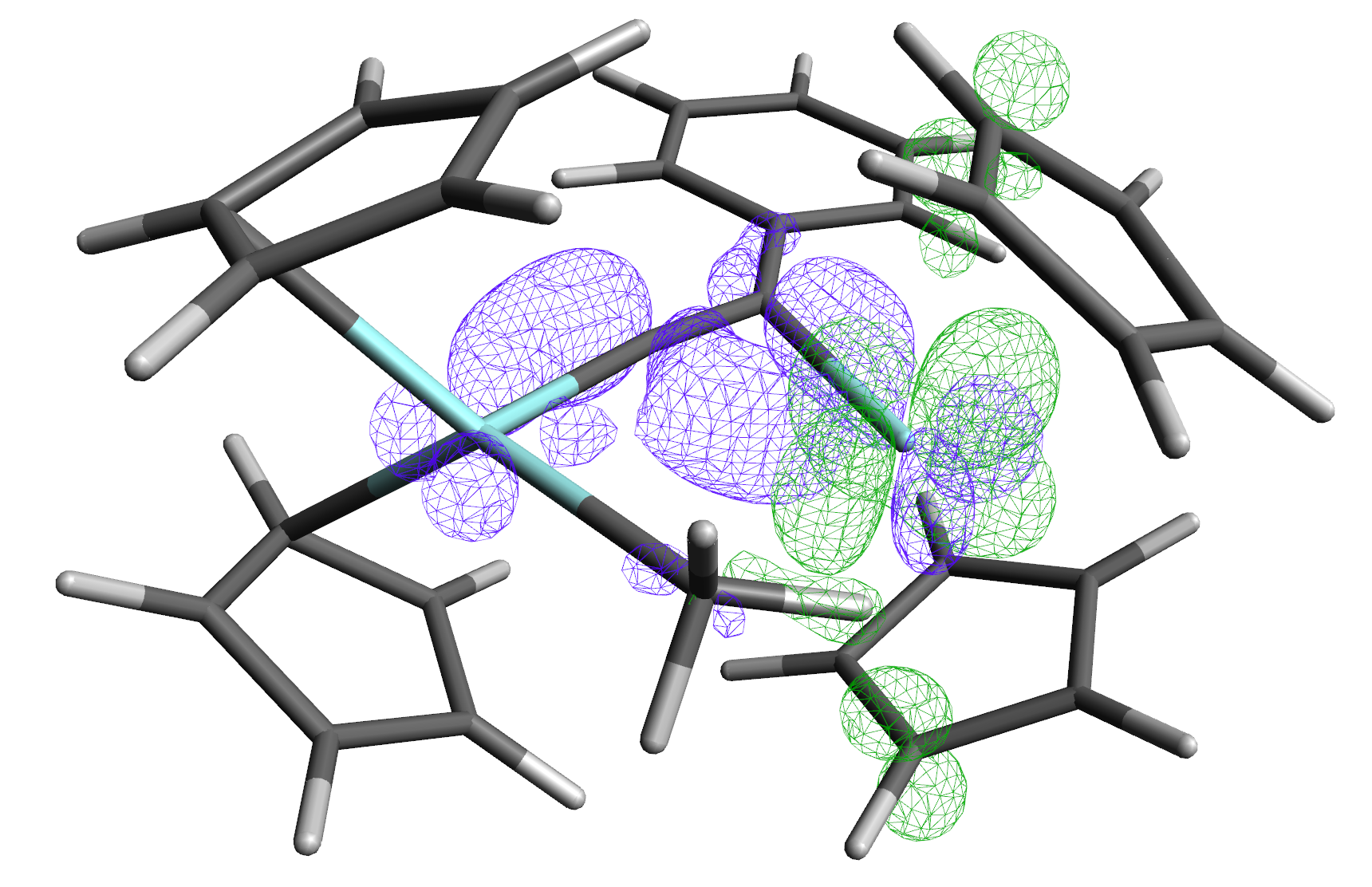 |  |

### **TD-DFT Analysis of (Cp_2_Zr)_2_(µ-H)(µ-C_2_Ph) (6)**

**Figure S30.** Plot of the theoretical (TD-DFT) UV-vis spectrum of **6** suggesting a half-width at half-height of 0.25 eV. This reveals the first two excited states as responsible for the intense blue colour of the complex.

Excitation energies and oscillator strengths:

Excited State 1: Singlet-A 2.3892 eV 518.95 nm f=0.0544 <S**2>=0.000

109 -> 110 0.56756

109 -> 111 0.38619

109 -> 113 -0.10158

This state for optimization and/or second-order correction.

Total Energy, E(TD-HF/TD-DFT) = -1175.94266295

Copying the excited state density for this state as the 1-particle RhoCI density.

Excited State 2: Singlet-A 2.4128 eV 513.87 nm f=0.0304 <S**2>=0.000

109 -> 110 -0.37041

109 -> 111 0.58477

Excited State 3: Singlet-A 3.3150 eV 374.01 nm f=0.0009 <S**2>=0.000

108 -> 110 0.70207

Excited State 4: Singlet-A 3.4711 eV 357.19 nm f=0.0008 <S**2>=0.000

109 -> 112 0.58505

109 -> 114 -0.34975

109 -> 118 -0.12315

- The two first exited states are the most prominent ones in the Vis region and are responsible for the colour of this complex. Therefore, the following analysis was focussed on these first two exited states.

**Table S16.** Representation of the molecular orbitals which are involved in the first two excited states of complex **6** (PBE0-D3/def2-TZVP).

| HOMO 109 (iso0.04, -4.788 eV) | LUMO 110 (iso0.04, -1.438 eV) |
| --- | --- |
| 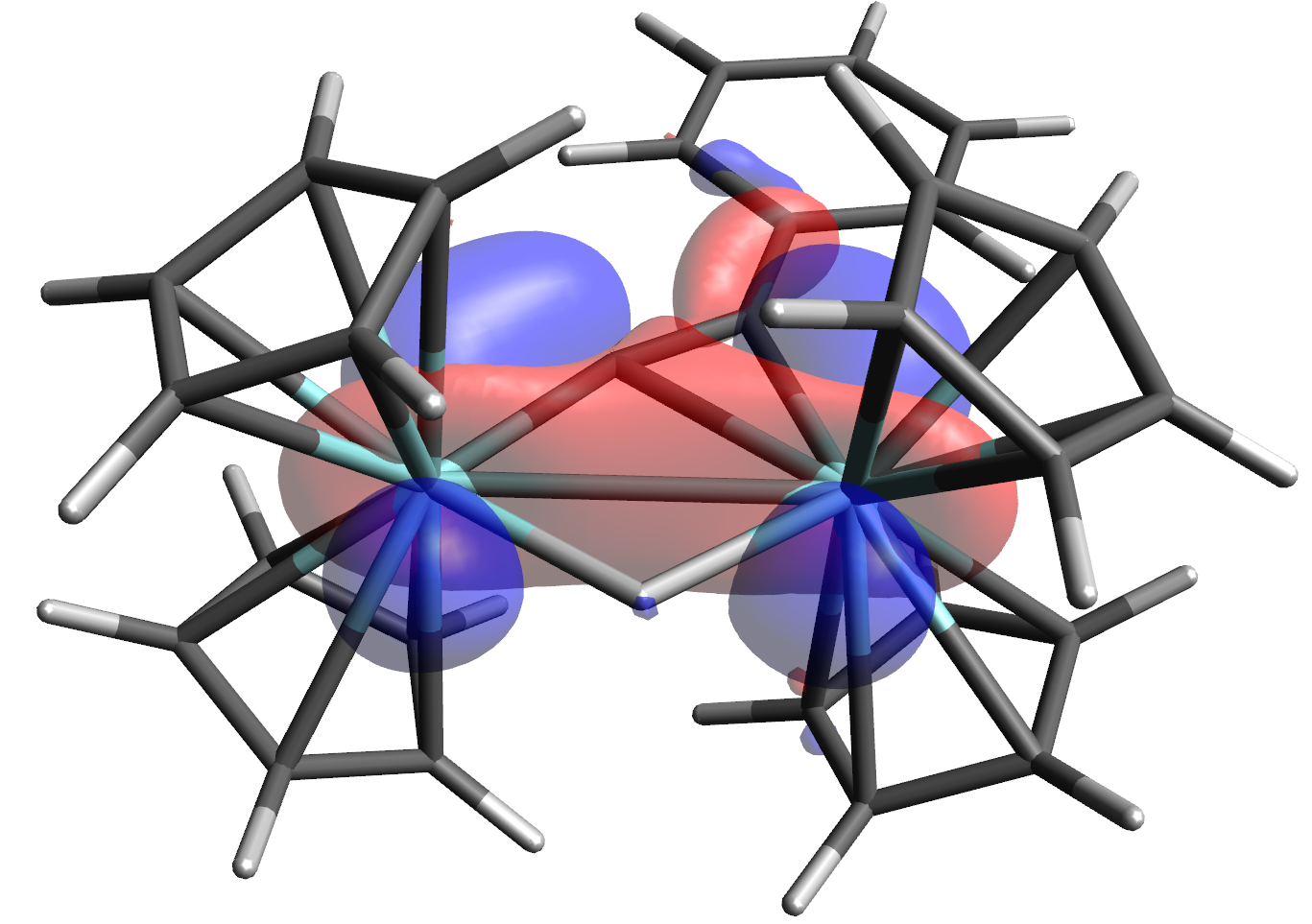 | 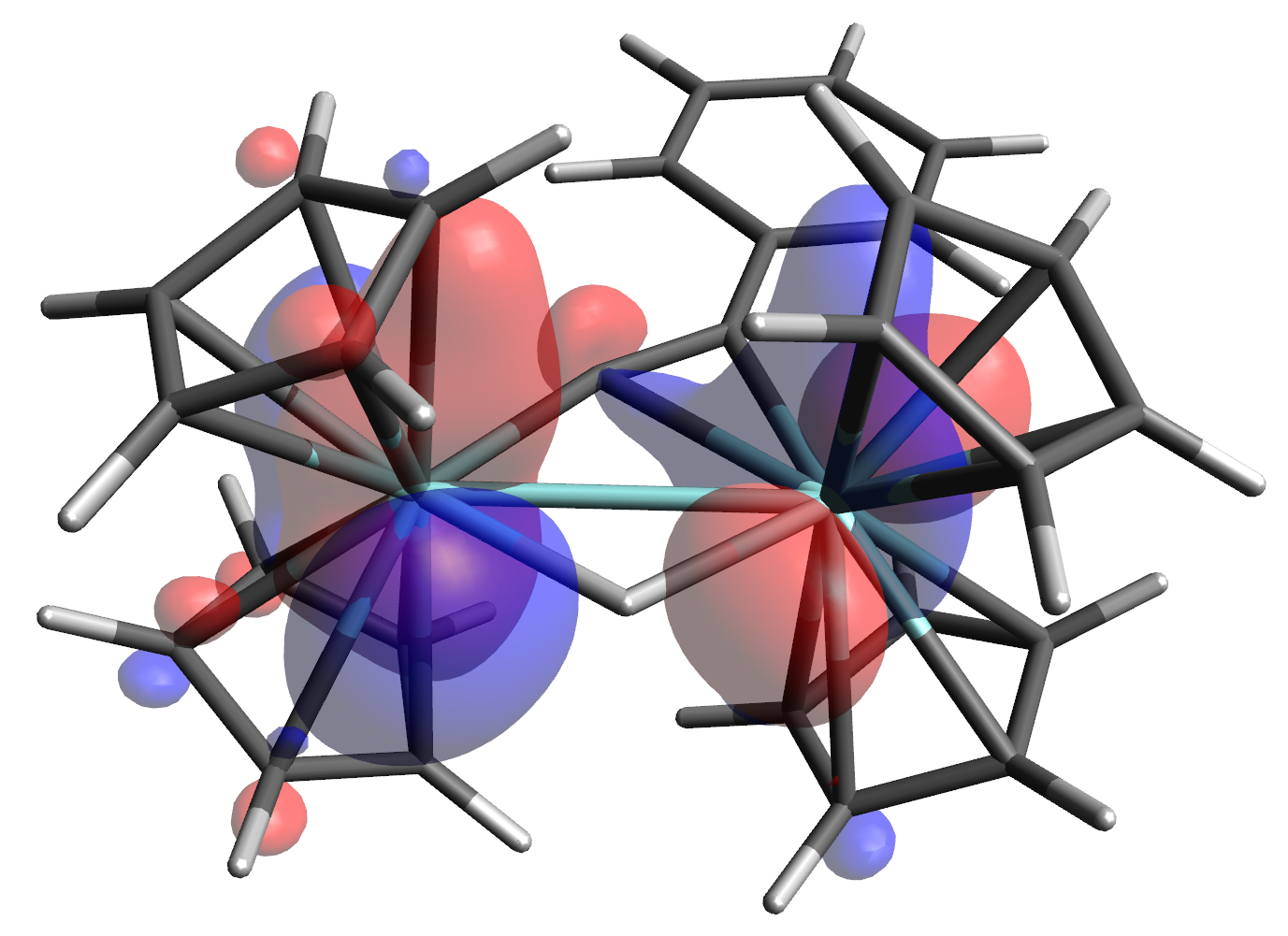 |
| LUMO+1 111 (iso0.04, -0.108 eV) | LUMO+3 113 (iso0.04, -0.069 eV) |
| 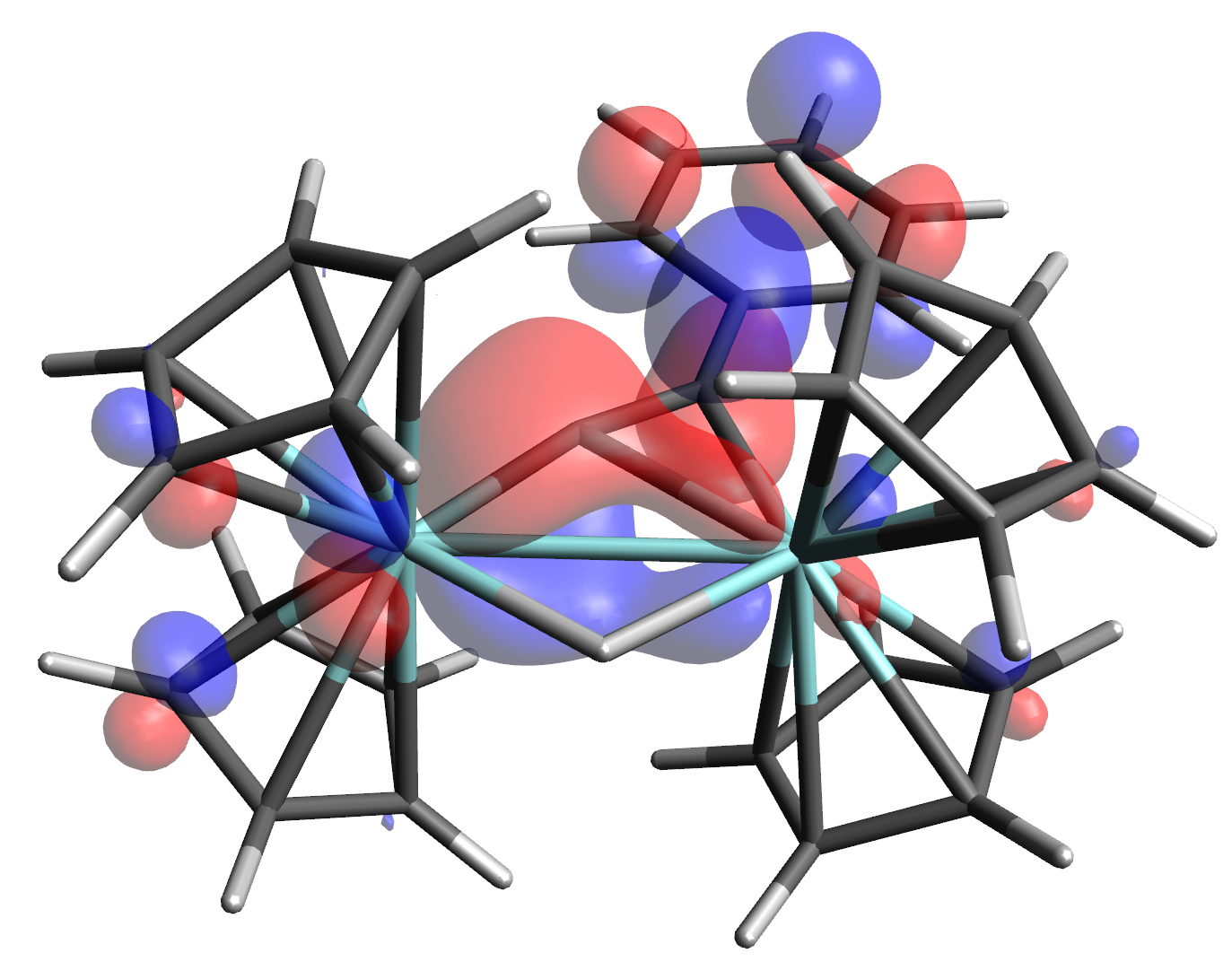 | 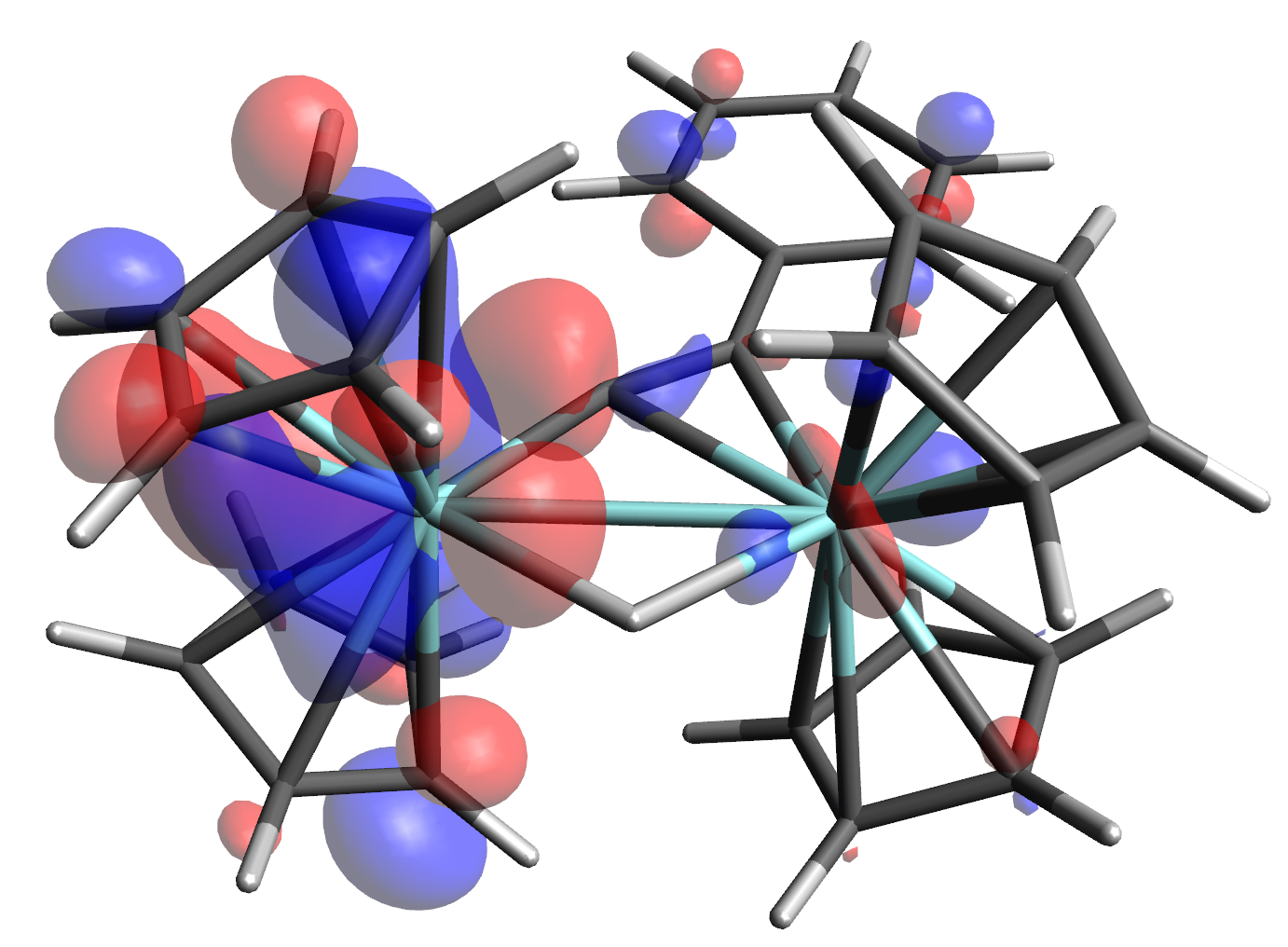 |

**Table S17.** Analysis of the charge density differences (CCD) of excited states of complex **6** (green regions are electron accepting and blue regions electron donating due to the excitation, PBE0-D3/def2-TZVP)

| CDD EX1 (iso0.004) | CDD EX2 (iso0.004) |
| --- | --- |
| 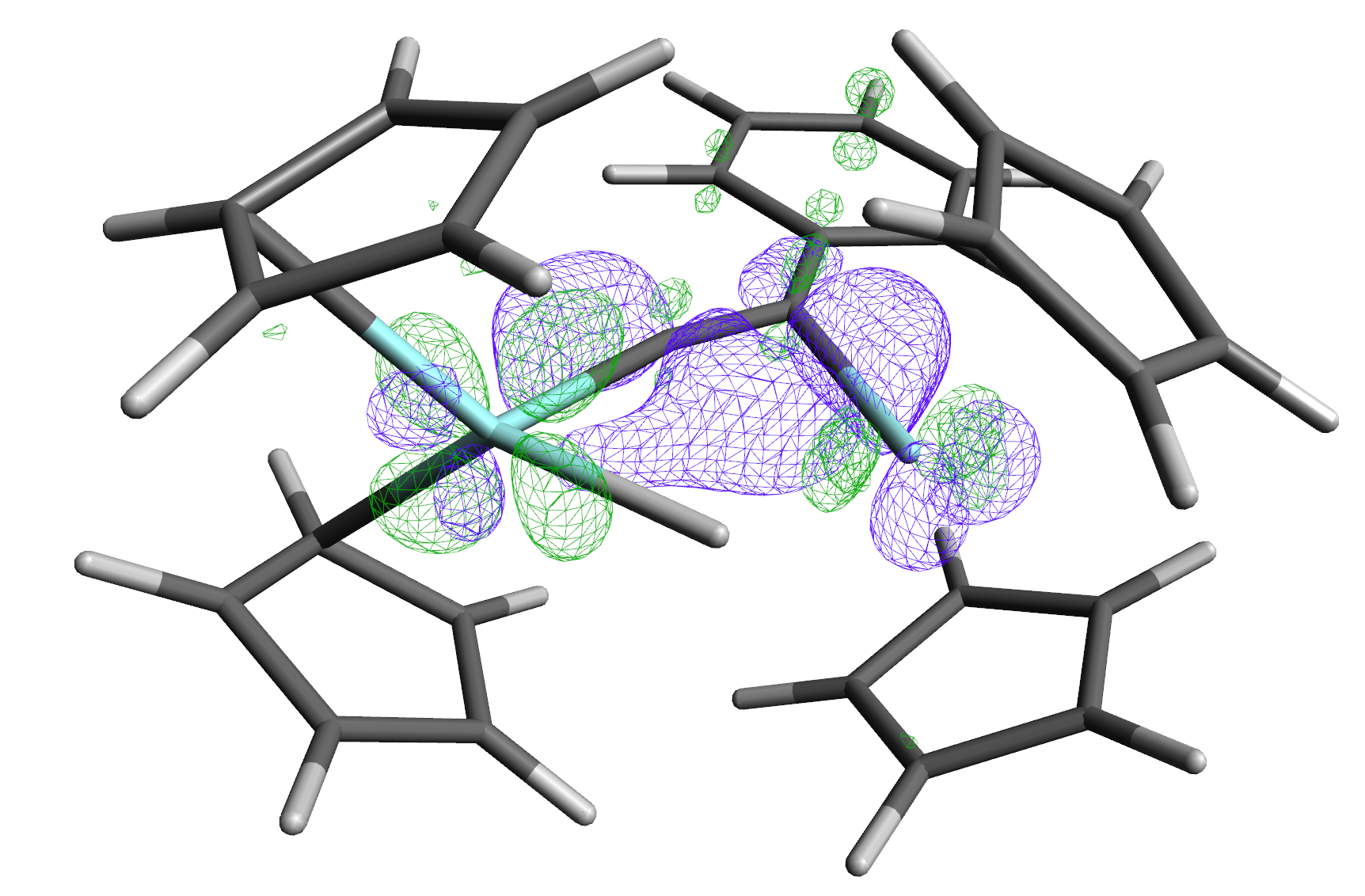 | 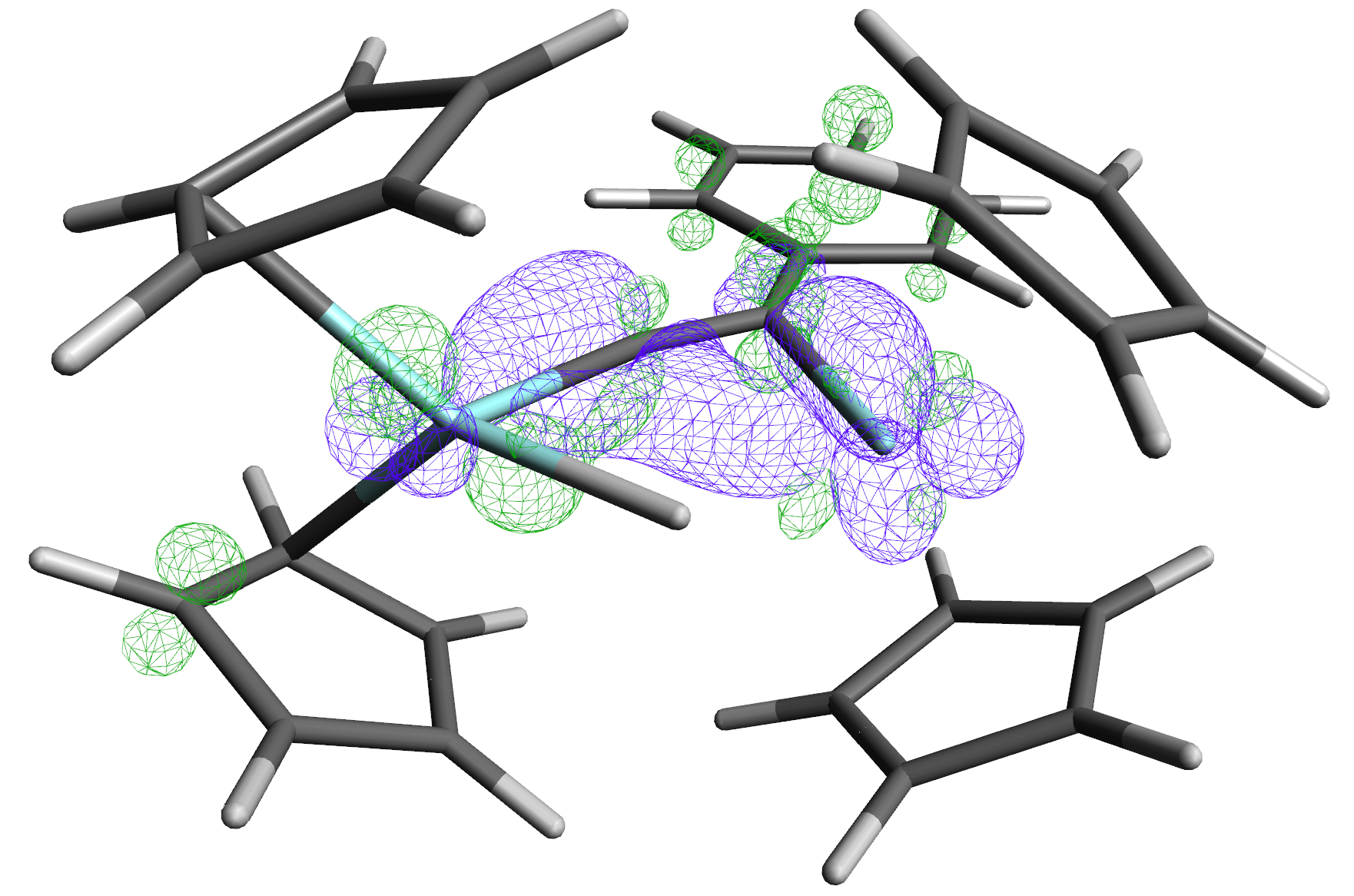 |

### **TD-DFT analysis of (Cp_2_Zr)_3_(µ-H_2_)(σ- µ -C=CHPh) (7)**

**Figure S31.** Plot of the theoretical (TD-DFT) UV-vis spectrum of **7** suggesting a half-width at half-height of 0.25 eV. This reveals the first six excited states as responsible for the intense purple colour of the complex.

Excitation energies and oscillator strengths:

Excited State 1: Singlet-A 2.0745 eV 597.65 nm f=0.0007 <S**2>=0.000

150 -> 152 0.19176

151 -> 152 0.67654

This state for optimization and/or second-order correction.

Total Energy, E(TD-HF/TD-DFT) = -1611.08626893

Copying the excited state density for this state as the 1-particle RhoCI density.

Excited State 2: Singlet-A 2.4887 eV 498.18 nm f=0.0598 <S**2>=0.000

150 -> 152 0.64660

151 -> 152 -0.18131

151 -> 153 0.17248

Excited State 3: Singlet-A 3.1664 eV 391.57 nm f=0.0072 <S**2>=0.000

150 -> 153 0.64460

151 -> 153 -0.25155

Excited State 4: Singlet-A 3.2416 eV 382.48 nm f=0.0405 <S**2>=0.000

150 -> 153 0.21030

150 -> 154 0.37954

151 -> 153 0.50314

151 -> 154 -0.18932

Excited State 5: Singlet-A 3.3361 eV 371.64 nm f=0.0177 <S**2>=0.000

151 -> 153 0.13132

151 -> 154 0.66184

Excited State 6: Singlet-A 3.3966 eV 365.03 nm f=0.0880 <S**2>=0.000

148 -> 152 -0.13680

150 -> 152 0.12585

150 -> 153 -0.10268

150 -> 154 0.54939

151 -> 153 -0.32071

Excited State 7: Singlet-A 3.4441 eV 359.99 nm f=0.0007 <S**2>=0.000

150 -> 155 0.15180

151 -> 155 0.66626

151 -> 158 0.10690

- The first three exited states are the only ones which are tailing in the Vis region and are responsible for the colour of this complex. Therefore, the following analysis was focussed on these first exited states.

**Table S18.** Representation of the molecular orbitals which are involved in the first two excited states of complex **7**. Hydrogen atoms are omitted for clarity (PBE0-D3/def2-TZVP).

| HOMO-3 148 (iso0.04, -6.365) | HOMO-2 149 (iso0.04, -6.187) |
| --- | --- |
| 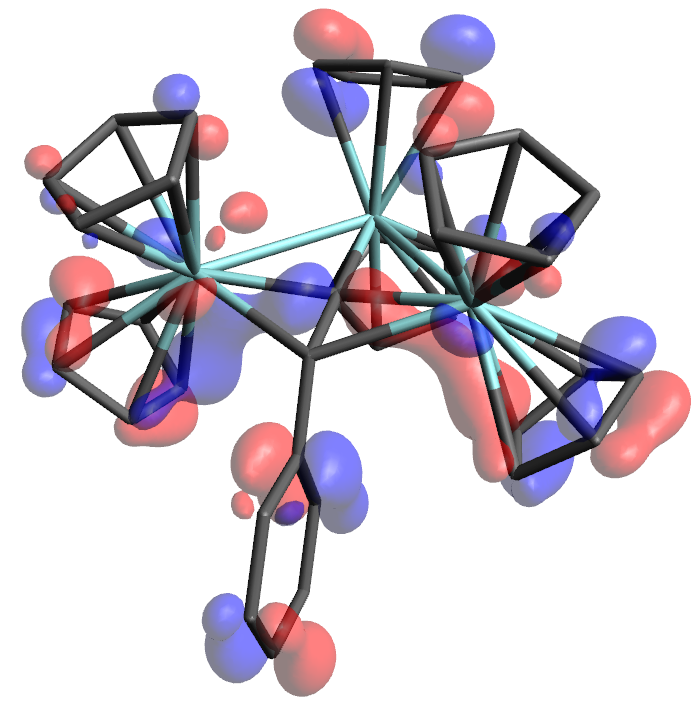 | 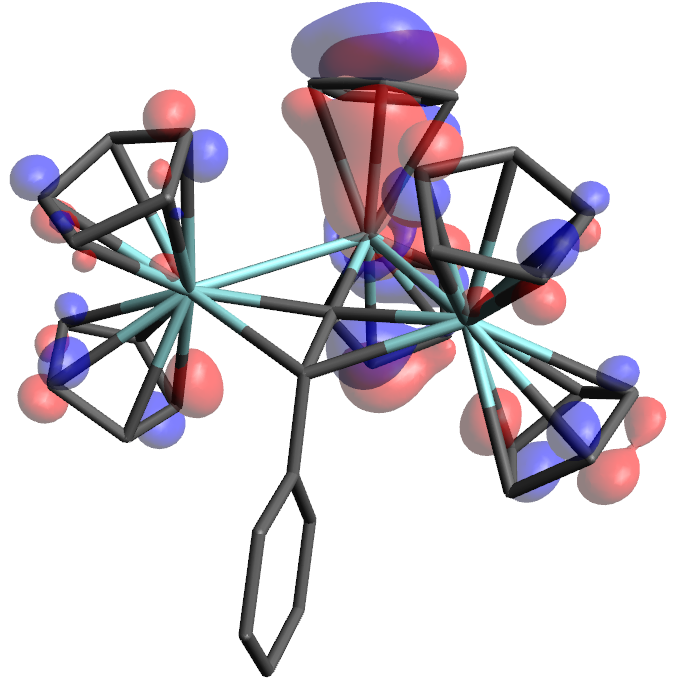 |
| HOMO-1 150 (iso0.04, -4.957 eV) | HOMO 151 (iso0.04, -4.908 eV) |
| 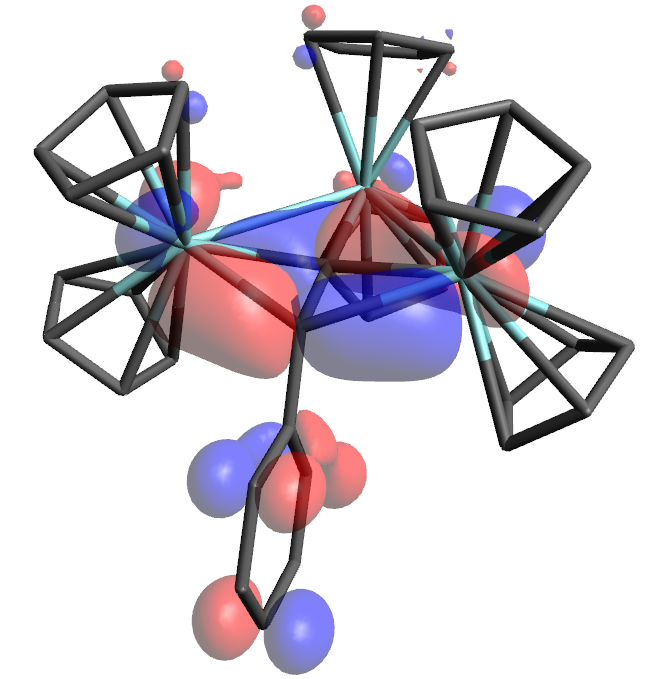 | 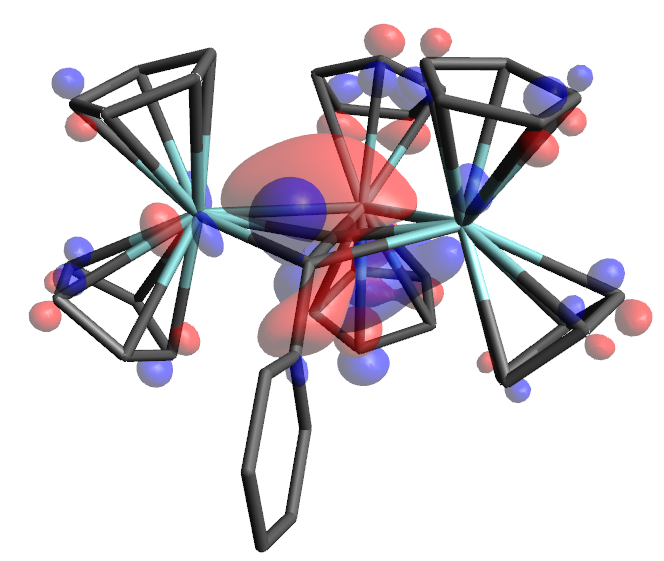 |
| LUMO 152 (iso0.04, -1.677 eV) | LUMO+1 153 (iso0.04, -0.663 eV) |
| 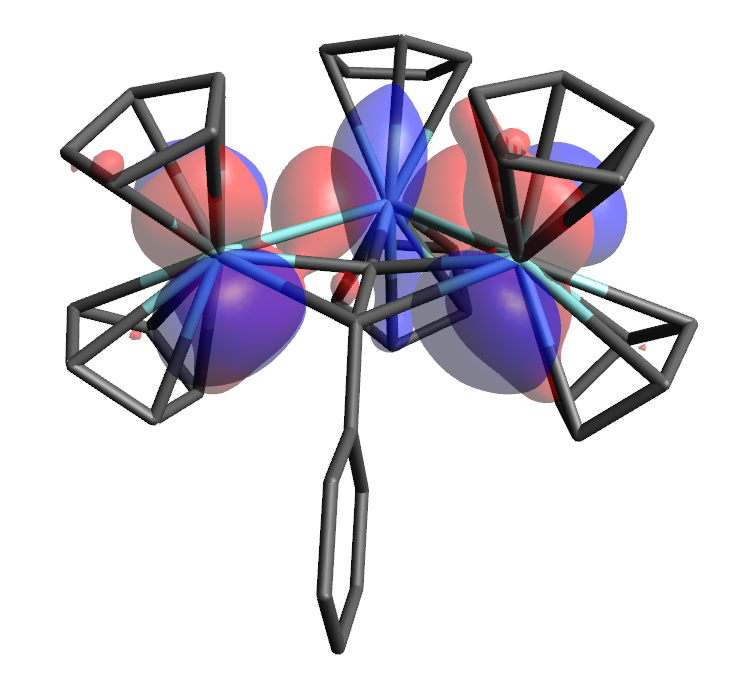 | 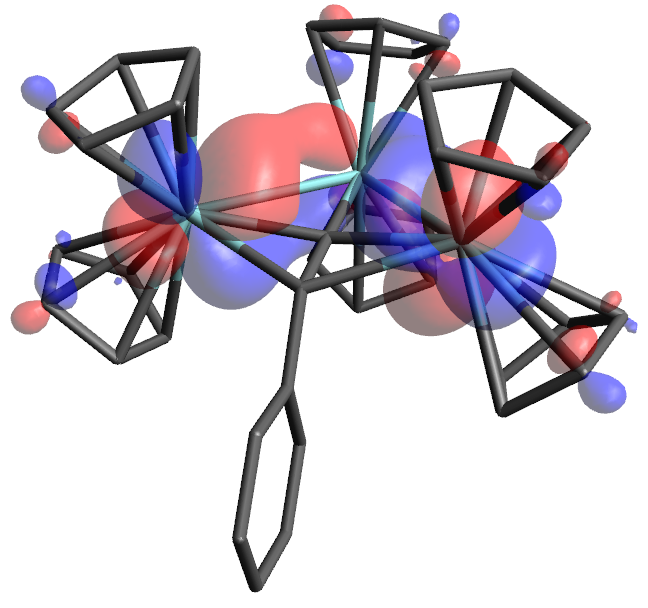 |
| LUMO+2 154 (iso0.04, -0.502 eV) |  |
| 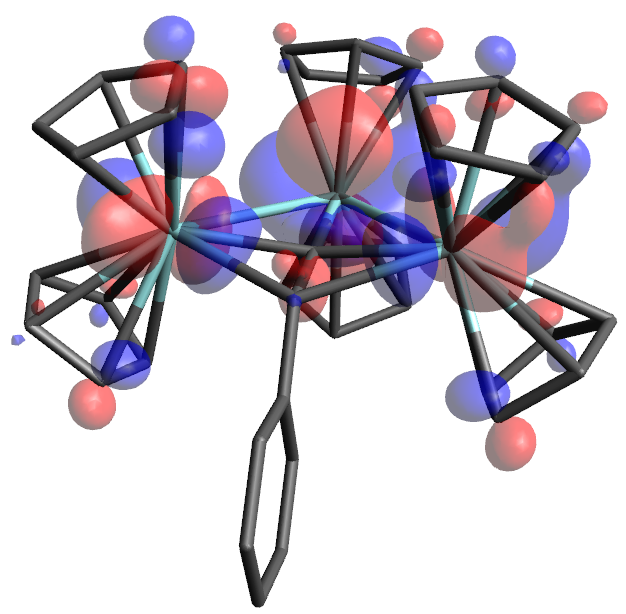 |  |

**Table S19.** Analysis of the charge density differences (CCD) of excited states of complex **7**. Hydrogen atoms are omitted for clarity (green regions are electron accepting and blue regions electron donating due to the excitation, PBE0-D3/def2-TZVP).

| CDD EX1 (iso0.004) | CDD EX2 (iso0.004) |
| --- | --- |
| 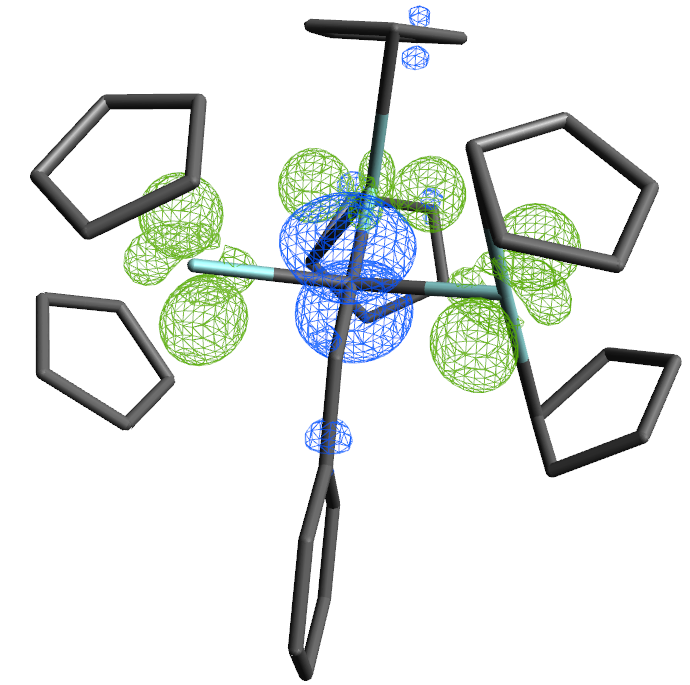 | 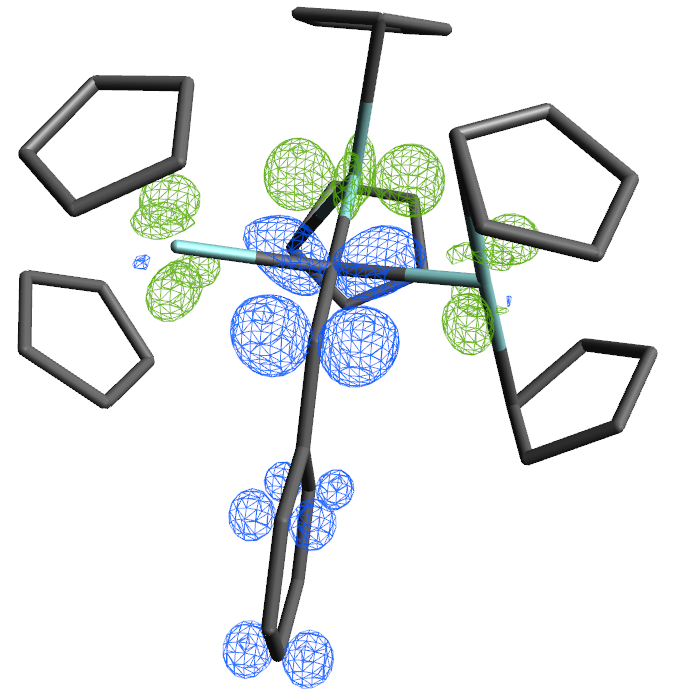 |
| CDD EX3 (iso0.004) | CDD EX4 (iso0.004) |
| 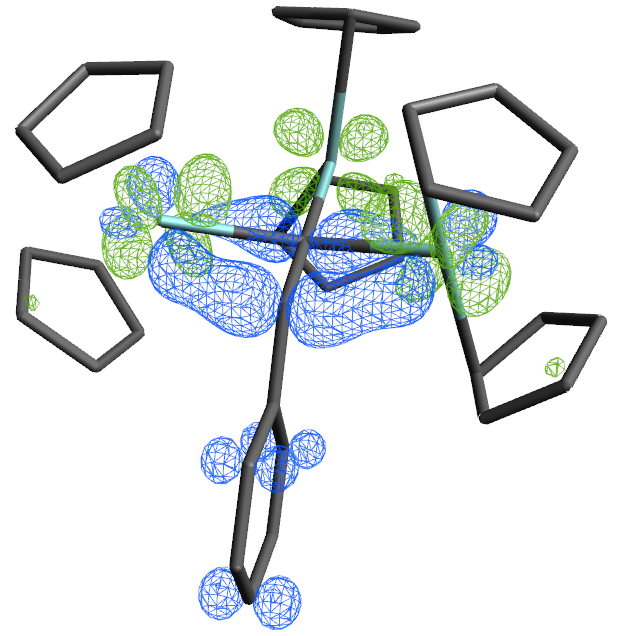 | 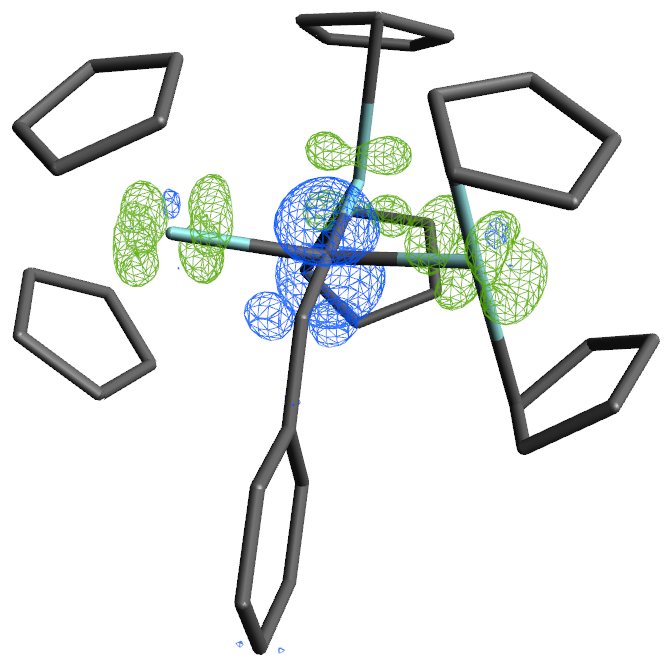 |

| CDD EX5 (iso0.004) | CDD EX6 (iso0.004) |
| --- | --- |
| 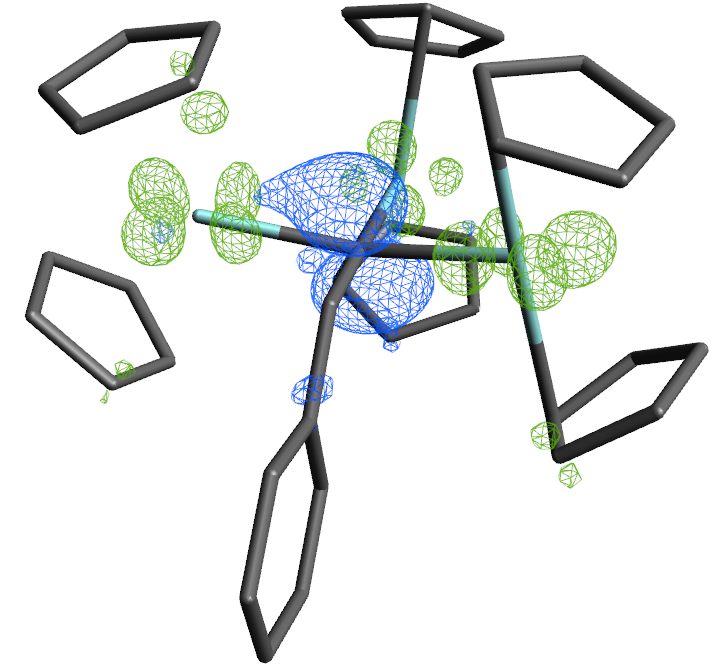 | 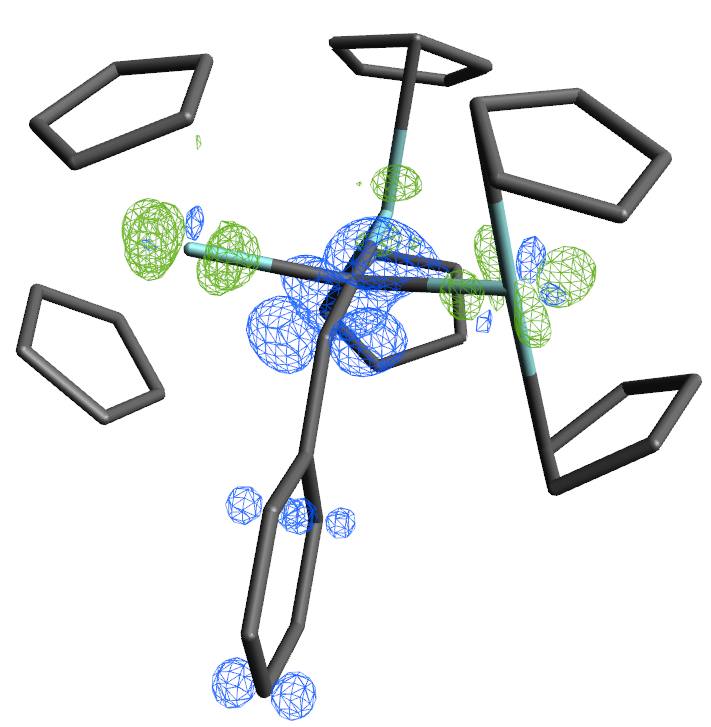 |

### **TD-DFT analysis of (Cp_2_Zr)2(CN-Py)Ph (8)**

**Figure S32.** Plot of the theoretical (TD-DFT) UV-vis spectrum of **8** suggesting a half-width at half-height of 0.25 eV. This reveals the first six excited states as responsible for the orange colour of the complex.

Excitation energies and oscillator strengths:

Excited State 1: Singlet-A 2.2493 eV 551.21 nm f=0.0004 <S**2>=0.000

138 -> 141 0.14570

139 -> 141 -0.16306

140 -> 141 0.65787

This state for optimization and/or second-order correction.

Total Energy, E(TD-HF/TD-DFT) = -1555.52222025

Copying the excited state density for this state as the 1-particle RhoCI density.

Excited State 2: Singlet-A 3.2507 eV 381.41 nm f=0.0002 <S**2>=0.000

138 -> 142 0.12155

139 -> 141 -0.18936

139 -> 142 -0.14196

140 -> 141 -0.17416

140 -> 142 0.61619

Excited State 3: Singlet-A 3.3346 eV 371.81 nm f=0.0013 <S**2>=0.000

137 -> 141 0.24249

138 -> 141 -0.16838

139 -> 141 0.57717

140 -> 141 0.14889

140 -> 142 0.22145

Excited State 4: Singlet-A 3.3854 eV 366.23 nm f=0.0008 <S**2>=0.000

137 -> 141 0.64556

139 -> 141 -0.21562

140 -> 142 -0.13125

Excited State 5: Singlet-A 3.4619 eV 358.14 nm f=0.0102 <S**2>=0.000

136 -> 141 -0.11476

138 -> 141 0.65007

139 -> 141 0.19860

Excited State 6: Singlet-A 3.6309 eV 341.47 nm f=0.0270 <S**2>=0.000

136 -> 141 0.68757

Excited State 7: Singlet-A 3.8242 eV 324.21 nm f=0.0030 <S**2>=0.000

135 -> 141 0.69700

- Due to the low oscillator strength of the first four exited stated these might have a lower contribution on the colour of this complex and the tailing of the fifth and sixth exited states show more contributions to the macroscopically observed orange colour of this complex **8**. Nevertheless, the following analysis was performed on these six exited states.

**Table S20.** Representation of the molecular orbitals which are involved in the first six excited states of complex **8**. Hydrogen atoms are omitted for clarity (PBE0-D3/def2-TZVP).

| HOMO-4 136 (iso0.04, -6.477) | HOMO-3 137 (iso0.04, -6.428) |
| --- | --- |
| 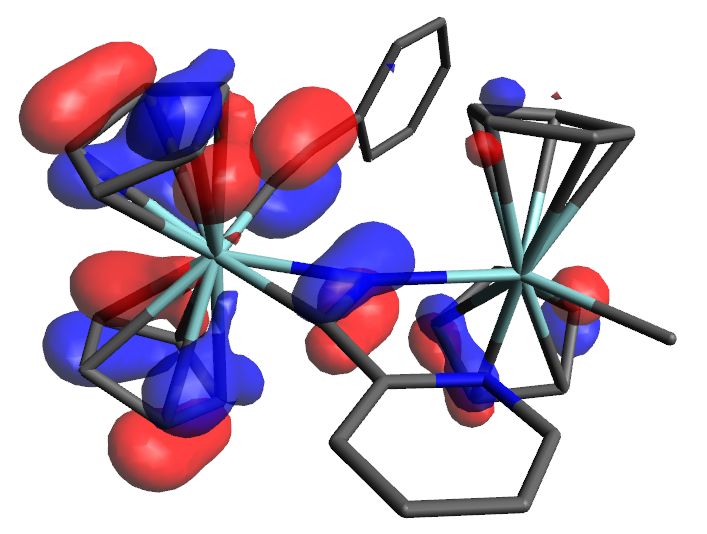 | 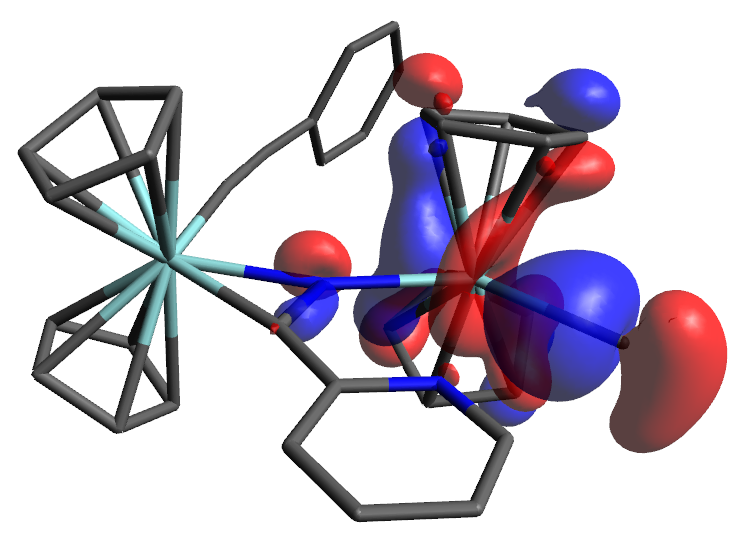 |
| HOMO-2 138 (iso0.04, -6.312 eV) | HOMO-1 139 (iso0.04, -6.181 eV) |
| 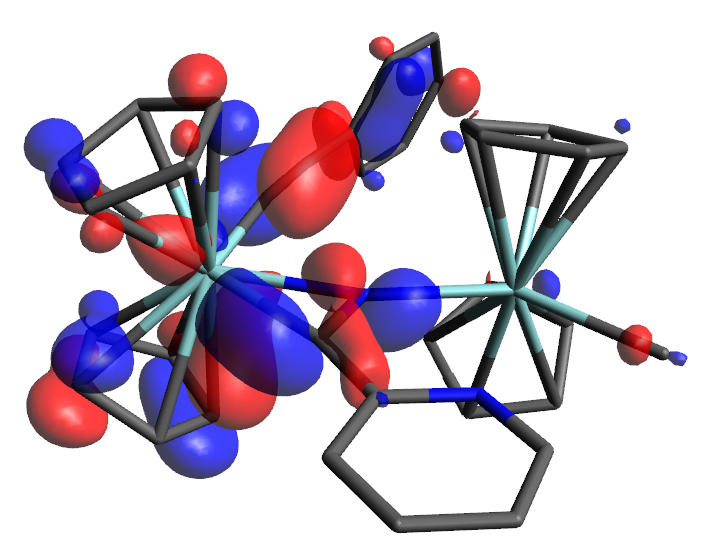 |  |
| HOMO 140 (iso0.04, -5.477 eV) | LUMO 141 (iso0.04, -2.125eV) |
|  |  |

| LUMO+1 142 (iso0.04, -1.315 eV) |  |
| --- | --- |
|  |  |

**Table S21.** Analysis of the charge density differences (CCD) of excited states of complex **7**. Hydrogen atoms are omitted for clarity (green regions are electron accepting and blue regions electron donating due to the excitation, PBE0-D3/def2-TZVP).

| CDD EX1 (iso0.004) | CDD EX2 (iso0.004) |
| --- | --- |
|  |  |
| CDD EX3 (iso0.004) | CDD EX4 (iso0.004) |
|  |  |

| CDD EX5 (iso0.004) | CDD EX6 (iso0.004) |
| --- | --- |
|  |  |

### **TD-DFT Analysis of (Cp_2_Zr)_2_(NCCH_3_)(H_3_CC_2_Ph) (9)**

**Figure S33.** Plot of the theoretical (TD-DFT) UV-vis spectrum of **9** suggesting a half-width at half-height of 0.25 eV. This reveals the first three excited states as responsible for the pale yellow colour of the complex.

Excitation energies and oscillator strengths:

Excited State 1: Singlet-A 3.2130 eV 385.88 nm f=0.0011 <S**2>=0.000

123 -> 125 0.55969

124 -> 125 -0.41458

This state for optimization and/or second-order correction.

Total Energy, E(TD-HF/TD-DFT) = -1347.88897824

Copying the excited state density for this state as the 1-particle RhoCI density.

Excited State 2: Singlet-A 3.4181 eV 362.73 nm f=0.0001 <S**2>=0.000

123 -> 125 0.41197

124 -> 125 0.56286

Excited State 3: Singlet-A 3.4954 eV 354.70 nm f=0.0023 <S**2>=0.000

123 -> 126 0.10215

124 -> 126 0.68566

- The first three exited states are the only ones which are tailing in the Vis region and are responsible for the colour of this complex. Therefore, the following analysis was focussed on these first exited states.

**Table S22.** Representation of the molecular orbitals which are involved in the first two excited states of complex **9**. Hydrogen atoms are omitted for clarity (PBE0-D3/def2-TZVP).

| HOMO-1 123 (iso0.04, -5.669 eV) | HOMO 124 (iso0.04, -5.375 eV) |
| --- | --- |
|  |  |
| LUMO 125 (iso0.04, -0.919 eV) | LUMO+1 126 (iso0.04, -0.696 eV) |
|  |  |

**Table S23.** Analysis of the charge density differences (CCD) of excited states of complex **9**. Hydrogen atoms are omitted for clarity (green regions are electron accepting and blue regions electron donating due to the excitation, PBE0-D3/def2-TZVP)

| CDD EX1 (iso0.004) | CDD EX2 (iso0.004) |
| --- | --- |
|  |  |
| CDD EX3 (iso0.004) |  |
|  |  |

# References

1. [] J. M. Camara, R. A. Petros and J. R. Norton, *J. Am. Chem. Soc.* **2011**, *133*, 5263-5273. [↑](#endnote-ref-1)
2. [] P. J. Walsh, F. J. Hollander and R. G. Bergman, *J* *Am. Chem. Soc.* **1988**, *110*, 8729-8731. [↑](#endnote-ref-2)
3. [] J. R. Nitschke, S. Züricher and T. D. Tilley, *J.Am. Chem. Soc.* **2000**, *122*, 10345-10352. [↑](#endnote-ref-3)
4. [] G. R. Fulmer, A. J. M. Miller, N. H. Sherden, H. E. Gottlieb, A. Nudelman, B. M. Stoltz, J. E. Bercaw and K. I. Goldberg, *Organometallics* **2010**, *29*, 2176-2179. [↑](#endnote-ref-4)
5. [] G. M. Sheldrick, *Acta Cryst.,* *Sect. A: Found. Crystallogr.* **2008**, *64*, 112-122. [↑](#endnote-ref-5)
6. [] G. M. Sheldrick, *Acta Cryst.*, *Sect. C: Struct. Chem.* **2015**, *71*, 3-8. [↑](#endnote-ref-6)
7. [] H. Putz and K. Brandenburg, *Diamond-Crystal and Molecular Structure Visualization*, Crystal Impact-GbR, Kreuzherrenstr. 102, 53227, Bonn, Germany, **2018**. [↑](#endnote-ref-7)
8. [] S. Chen, J. Wang and H. Wang, *Materials & Design* **2016**, *90*, 84-90. [↑](#endnote-ref-8)
9. [] M. J. Frisch, G. W. Trucks, H. B. Schlegel, G. E. Scuseria, M. A. Robb, J. R. Cheeseman, G. Scalmani, V. Barone, G. A. Petersson, H. Nakatsuji, X. Li, M. Caricato, A. V. Marenich, J. Bloino, B. G. Janesko, R. Gomperts, B. Mennucci, H. P. Hratchian, J. V. Ortiz, A. F. Izmaylov, J. L. Sonnenberg, D. Williams-Young, F. Ding, F. Lipparini, F. Egidi, J. Goings, B. Peng, A. Petrone, T. Henderson, D. Ranasinghe, V. G. Zakrzewski, J. Gao, N. Rega, G. Zheng, W. Liang, M. Hada, M. Ehara, K. Toyota, R. Fukuda, J. Hasegawa, M. Ishida, T. Nakajima, Y. Honda, O. Kitao, H. Nakai, T. Vreven, K. Throssell, J. A., Jr. Montgomery, J. E. Peralta, F. Ogliaro, M. J. Bearpark, J. J. Heyd, E. N. Brothers, K. N. Kudin, V. N. Staroverov, T. A. Keith, R. Kobayashi, J. Normand, K. Raghavachari, A. P. Rendell, J. C. Burant, S. S. Iyengar, J. Tomasi, M. Cossi, J. M. Millam, M. Klene, C. Adamo, R. Cammi, J. W. Ochterski, R. L. Martin, K. Morokuma, O. Farkas, J. B. Foresman and D. J. Fox, *Gaussian 16, Rev. C.01*, Gaussian, Inc., Wallingford, CT, 2016. [↑](#endnote-ref-9)
10. [] S. K. Podiyanachari, G. Bender, C. G. Daniliuc, G. Kehr and G. Erker, *Organometallics* **2014**, *33*, 3481-3488. [↑](#endnote-ref-10)
11. [] (a) E. D. Glendening, J. K. Badenhoop, A. E. Reed, J. E. Carpenter, J. A. Bohmann, C. M. Morales, C. R. Landis and F. Weinhold, Theoretical Chemistry Institute, University of Wisconsin, Madison, **2013**; (b) J. E. Carpenter and F. Weinhold, *J. Mol. Struct.: THEOCHEM* **1988**, *169*, 41-62; (c) F. Weinhold and J. E. Carpenter, in *The structure of small molecules and ions*, Springer, **1988**, 227-236; (d) F. Weinhold and C. R. Landis, *Valency and bonding: a natural bond orbital donor-acceptor perspective*, Cambridge University Press, **2005**. [↑](#endnote-ref-11)
12. [] (a) A. D. Becke, *Phys. Rev. A* **1988**, *38*, 3098-3100; (b) J. P. Perdew, *Phys. Rev. B* **1986**, *33*, 8822-8824. [↑](#endnote-ref-12)
13. [] (a) S. H. Vosko, L. Wilk and M. Nusair, *Can. J. Phys.* **1980**, *58*, 1200-1211; (b) C. Lee, W. Yang, R. G. Parr, *Phys. Rev. B* **1988**, *37*, 785-789; (c) B. Miehlich, A. Savin, H. Stoll and H. Preuss, *Chem. Phys. Lett.* **1989**, *157*, 200-206; (d) A. D. Becke, *J. Chem. Phys.* **1993**, *98*, 5648-5652. [↑](#endnote-ref-13)
14. [] F. Weigend and R. Ahlrichs, *Phys. Chem. Chem. Phys.* **2005**, *7*, 3297-3305. [↑](#endnote-ref-14)
15. [] (a) S. Grimme, J. Antony, S. Ehrlich and H. Krieg, *J. Chem. Phys.* **2010**, *132*, 154104; (b) S. Grimme, S. Ehrlich and L. Goerigk, *J. Comput. Chem.* **2011**, *32*, 1456-1465. [↑](#endnote-ref-15)
16. [] T. Lu and F. Chen, *J. Comput. Chem.* **2012**, *33*, 580-592. [↑](#endnote-ref-16)
17. [] a) G. Knizia, *J. Chem. Theo. Comp.* **2013**, *9*, 4834-4843; b) G. Knizia, J. E. M. N. Klein, *Ange. Chem. Int. Ed.* **2015**, *54*, 5518-5522. [↑](#endnote-ref-17)
18. [] T. Lu and Q. Chen, *Chemistry–Methods*, ***2021***, *1*, 231-239. [↑](#endnote-ref-18)
19. [] a) J. P. Perdew, K. Burke, and M. Ernzerhof *Phys. Rev. Lett.* **1996**, 77, 3865-68; b) J. P. Perdew, K. Burke, and M. Ernzerhof *Phys. Rev. Lett.* **1997**, 78,1396. [↑](#endnote-ref-19)
20. [] Z. Liu, X. Wang, T. Lu, A. Yuan and X. Yan, *Carbon* **2022**, *187*, 78-85. [↑](#endnote-ref-20)
21. [] R. Dennington, T. A. Keith and J. M. Millam, GaussView, version 6.0. 16. *Semichem Inc. Shawnee Mission KS*, **2016**. [↑](#endnote-ref-21)
22. [] Marcus D Hanwell, Donald E Curtis, David C Lonie, Tim Vandermeersch, Eva Zurek and Geoffrey R Hutchison; “Avogadro: An advanced semantic chemical editor, visualization, and analysis platform” *Journal of Cheminformatics* **2012**, *4*, 17. [↑](#endnote-ref-22)
23. [] Mercury: <http://www.ccdc.cam.ac.uk/mercury/> [↑](#endnote-ref-23)
